# Effects of High Toxic Boron Concentration on Protein Profiles in Roots of Two Citrus Species Differing in Boron-Tolerance Revealed by a 2-DE Based MS Approach

**DOI:** 10.3389/fpls.2017.00180

**Published:** 2017-02-17

**Authors:** Wen Sang, Zeng-Rong Huang, Lin-Tong Yang, Peng Guo, Xin Ye, Li-Song Chen

**Affiliations:** ^1^Institute of Plant Nutritional Physiology and Molecular Biology, College of Resources and Environment, Fujian Agriculture and Forestry UniversityFuzhou, China; ^2^College of Horticulture, Fujian Agriculture and Forestry UniversityFuzhou, China; ^3^Agriculture, Forestry and Water Conservancy Bureau of Xinzhou DistrictShangrao, China; ^4^Fujian Provincial Key Laboratory of Soil Environmental Health and Regulation, College of Resources and Environment, Fujian Agriculture and Forestry UniversityFuzhou, China; ^5^The Higher Educational Key Laboratory of Fujian Province for Soil Ecosystem Health and Regulation, Fujian Agriculture and Forestry UniversityFuzhou, China

**Keywords:** boron-toxicity, *Citrus grandis*, *Citrus sinensis*, 2-DE, proteome, roots

## Abstract

Citrus are sensitive to boron (B)-toxicity. In China, B-toxicity occurs in some citrus orchards. So far, limited data are available on B-toxicity-responsive proteins in higher plants. Thirteen-week-old seedlings of “Sour pummelo” (*Citrus grandis*) and “Xuegan” (*Citrus sinensis*) was fertilized every other day until dripping with nutrient solution containing 10 μM (control) or 400 μM (B-toxicity) H_3_BO_3_ for 15 weeks. The typical B-toxic symptom only occurred in 400 μM B-treated *C. grandis* leaves, and that B-toxicity decreased root dry weight more in *C. grandis* seedlings than in *C. sinensis* ones, demonstrating that *C. sinensis* was more tolerant to B-toxicity than *C. grandis*. Using a 2-dimensional electrophoresis (2-DE) based MS approach, we identified 27 up- and four down-accumulated, and 28 up- and 13 down-accumulated proteins in B-toxic *C. sinensis* and *C. grandis* roots, respectively. Most of these proteins were isolated only from B-toxic *C. sinensis* or *C. grandis* roots, only nine B-toxicity-responsive proteins were shared by the two citrus species. Great differences existed in B-toxicity-induced alterations of protein profiles between *C. sinensis* and *C. grandis* roots. More proteins related to detoxification were up-accumulated in B-toxic *C. grandis* roots than in B-toxic *C. sinensis* roots to meet the increased requirement for the detoxification of the more reactive oxygen species and other toxic compounds such as aldehydes in the former. For the first time, we demonstrated that the active methyl cycle was induced and repressed in B-toxic *C. sinensis* and *C. grandis* roots, respectively, and that *C. sinensis* roots had a better capacity to keep cell wall and cytoskeleton integrity than *C. grandis* roots in response to B-toxicity, which might be responsible for the higher B-tolerance of *C. sinensis*. In addition, proteins involved in nucleic acid metabolism, biological regulation and signal transduction might play a role in the higher B-tolerance of *C. sinensis*.

## Introduction

Boron (B) is an essential micronutrient for higher plants (Warington, 1923), where its most important role is associated with cell wall formation, functioning, and strength (Blevins and Lukaszewski, 1998). However, B will become toxic to crops when present in excess (Ben-Gal and Shani, 2003; Chen et al., 2012). B-toxicity is common in areas with high B concentration in underground water mainly resulting from the over-application of B fertilizer (Smith et al., 2013). In China, B-toxicity occurs in some citrus orchards. Up to 74.8 and 22.9% of pummelo (*Citrus grandis*) orchards in Pinghe, Zhangzhou, China, are excess in leaf B and soil water-soluble B, respectively (Li et al., 2015).

Plants have developed various mechanisms to cope with B-toxicity. Usually, antioxidant system will be activated to defense oxidative damage caused by B-toxicity (Cervilla et al., 2007; Ardic et al., 2009). Antioxidant compounds such as ascorbate and reduced glutathione (GSH) and antioxidant enzymes such as ascorbate peroxidase (APX), superoxide dismutase (SOD), catalase (CAT), and glutathione reductase (GR) are involved in the scavenging of reactive oxygen species (ROS) (Han et al., 2009; Erdal et al., 2014). B-tolerant plant leaves are characterized by a lower B concentration relative to B-sensitive ones, possibly due to a decreased uptake of B into both roots and shoots (leaves) (Camacho-Cristóbal et al., 2008). Sheng et al. (2010) showed that B-tolerant Newhall navel orange trees grafted on Carrizo citrange accumulated more B in roots and leaves than B-sensitive Skagg's Bonanza naval orange trees grafted on Carrizo citrange when exposed to B-toxicity, implying that the former must possess inner mechanisms to tolerate high level of B. Huang et al. (2014) reported that under B-toxicity, total B level was similar between B-tolerant *Citrus sinensis* and B-sensitive *C. grandis* roots (leaves), while *C. sinensis* leaves had lower free B and higher bound B than *C. grandis* leaves, which might contribute to the higher B-tolerance of *C. sinensis*. Our recent work with *C. sinensis* and *C. grandis* demonstrated that miR397a played a key role in citrus B-tolerance by targeting two laccase genes involved in secondary cell-wall biosynthesis (Huang et al., 2016). Similar result has been obtained on *Poncirus trifoliata* (Jin et al., 2016). To conclude, the mechanisms for plant B-tolerance are not fully understood yet.

A comprehensive investigation of B-toxicity-responsive proteins will be useful for us to unveil the inner mechanisms of B-tolerance in specific plant species. So far, knowledge on B-toxicity-induced alterations of protein profiles in higher plants is limited. Demiray et al. (2011) used a 2-dimensional electrophoresis (2-DE) based MS approach to identify six B-toxicity-responsive proteins from carrot roots. Atik et al. (2011) used a 2-DE technique to investigate the effects of B-toxicity on protein profiles in barley leaves, suggesting that a B-toxicity-responsive vacuolar H^+^-ATPase (V-ATPase) subunit E was involved in barley B-tolerance.

In higher plants, citrus are sensitive to B-toxicity (Eaton, 1935; Papadakis et al., 2004). Since B is phloem immobile in citrus (Konsaeng et al., 2005), the typical B-toxic symptom (chlorotic and/or necrotic patches) first develops in the older leaves and extends progressively from the old leaves to the young leaves (Han et al., 2009; Guo et al., 2014; Sang et al., 2015). It was indicated that great differences existed in B-tolerance among citrus species and/or genotypes (Chen et al., 2012). For example, when *C. sinensis* and *C. grandis* seedlings were submitted to 400 μM B for 15 weeks, the typical B-toxic symptom only occurred in the latter (Guo et al., 2014; Sang et al., 2015). We previously investigated the differences in B-toxicity-induced alterations of gene expression profiles in roots and leaves and of protein profiles in leaves between B-tolerant *C. sinensis* and B-sensitive *C. grandis* and revealed some adaptive responses of citrus to B-toxicity (Guo et al., 2014, 2016; Sang et al., 2015). Thus, B-toxicity-responsive proteins in roots should be different between *C. sinensis* and *C. grandis*. In this study, we used a 2-DE based MS approach to investigate comparatively B-toxicity-induced alterations of protein profiles in B-tolerant *C. sinensis* and B-sensitive *C. grandis* seedlings roots and corroborated the above hypothesis. For the first time, we demonstrated that the active methyl cycle was upregulated and downregulated in B-toxic *C. sinensis* and *C. grandis* roots, respectively, and that *C. sinensis* roots had a better capacity to keep cell wall and cytoskeleton integrity than *C. grandis* roots when exposed to B-toxicity, which might be involved in the higher B-tolerance of *C. sinensis*.

## Materials and methods

### Plant materials and culture conditions

This study was conducted at Fujian Agriculture and Forestry University, Fuzhou, China. Seeds of “Sour pummelo” (*C. grandis*) and “Xuegan” (*C. sinensis*) were germinated in clean river sand in plastic trays. Five weeks after germination, uniform seedlings with a single stem were transplanted to 6 L pots (two seedlings per pot) filled with clean river sand. Seedlings were grown in a greenhouse under natural photoperiod. Eight weeks after transplanting, each pot was fertilized every other day until dripping with nutrient solution (ca. 500 mL) containing 10 μM (control) or 400 μM (B-toxicity) H_3_BO_3_ for 15 weeks as described previously by Guo et al. (2014) and Sang et al. (2015). Thereafter, fully expanded (ca. 7-week-old) leaves were used for all the measurements. Leaf discs (0.2826 cm^2^ in size) were punched from each leaf using a hole puncher of 0.6 cm in diameter at noon at full sun and immediately frozen in liquid nitrogen. Approximately 5-mm-long white root apices were immediately frozen in liquid nitrogen after they were collected from the same seedlings used for sampling leaves. Both root and leaf samples were stored at −80°C until RNA and protein extraction, and the assay of malondialdehyde (MDA) concentration, H_2_O_2_ production and enzyme activities. The remaining seedlings that were not sampled were used to measure root dry weight (DW) and B concentration in fibrous roots, root apices and leaves.

### Measurements of root DW, and B and MDA concentrations and H_2_O_2_ production in roots and leaves

Roots of ten seedlings per treatment from 10 pots were harvested from the remaining seedlings and their DW was measured after being dried at 70°C for 48 h.

Fibrous roots, root apices and ca. 7-week-old fully expanded leaves (midribs and petioles removed) collected from the remaining seedlings were dried at 70°C, then ground to pass a 40-mesh sieve. Root and leaf B concentration was assayed by ICP emission spectrometry after microwave digestion with HNO_3_ (Wang et al., 2006). There were four replicates per treatment.

Root and leaf MDA was extracted and assayed according to Hodges et al. (1999). There were four replicates per treatment.

Root and leaf H_2_O_2_ production was determined according to Chen et al. (2005). About 40 mg frozen roots or 15 frozen leaf discs were incubated in 2 mL reaction mixture containing 50 mM of phosphate buffer (pH 7.0), 0.05% (w/v) of guaiacol and 5 U of horseradish peroxidase (Product No. 77332, lyophilized, powder, beige, ~150 U mg^−1^, Sigma-Aldrich, Shanghai, China) for 2 h at room temperature in the dark. Then, absorbance was assayed at 470 nm. There were four replicates per treatment.

### Root protein extraction, 2-DE and image analysis

Approximately 1 g frozen roots collected equally from five seedlings (one seedling per pot) were mixed as one biological replicate. There were three biological replicates for each treatment (total of 15 seedlings from 15 pots). Proteins were independently extracted thrice from B-toxic and control samples according to You et al. (2014) using a phenol extraction procedure in order to ensure result reproducibility. Sample protein concentration was assayed according to Bradford (1976). 2-DE and image analysis were made according to Sang et al. (2015) and You et al. (2014). Gel images were obtained using Epson Scanner (Seiko Epson Corporation, Japan) at 300 dpi resolution. Image analysis was performed with PDQuest version 8.0.1 (Bio-Rad, Hercules, CA, USA). The software was used to perform background subtraction, Gaussian fitting, gel alignment, spot detection, matching and normalization. The parameters used to spot detection were as follow: sensitivity 6.05, size scale 3, min peak 600, and local regression model was selected to conduct spot normalization. The spot intensity was expressed as relatively abundant intensity that normalized by total intensities of all spots in one gel. After manual processing, the candidate spots in all triplicate gels were submitted to ANOVA analysis. A protein spot was considered differentially abundant when it had both a *P* < 0.05 and a fold change of > 1.5.

### Protein identification by MALDI-TOF/TOF-MS and bioinformatic analysis

MALDI-TOF/TOF-MS-based protein identification was performed on an AB SCIEX 5800 TOF/TOF (AB SCIEX, Shanghai, China) according to You et al. (2014) and Peng et al. (2015). Briefly, spots were excised from the colloidal Coomassie Brilliant Blue stained gels and plated into a 96-well microtiter plate. Excised spots were first destained twice with 60 μL of 50 mM NH_4_HCO_3_ and 50% (v/v) acetonitrile, and then dried twice with 60 μL of acetonitrile. Afterwards, the dried pieces of gels were incubated in ice-cold digestion solution [trypsin (sequencing-grade modified trypsin V5113, Promega, Madison, WI, USA) 12.5 ng/μL and 20 mM NH_4_HCO_3_] for 20 min, and then transferred into a 37°C incubator for digestion overnight. Peptides in the supernatant were collected after extraction twice with 60 μL extract solution [5% (v/v) formic acid in 50% (v/v) acetonitrile]. The resulting peptide solution was dried under the protection of N_2_. Before MS/MS analysis, the pellet was redissolved in 0.8 μL matrix solution [5 mg/mL α-cyano-4-hydroxy-cinnamic acid diluted in 0.1% trifluoroacetic acid (TFA), 50% (v/v) acetonitrile]. Then the mixture was spotted onto a MALDI target plate (AB SCIEX, Shanghai, China). MS analysis of peptide was performed on an AB SCIEX 5800 TOF/TOF. The UV laser was operated at a 400 Hz repetition rate with a wavelength of 355 nm. The accelerated voltage was operated at 20 kV, and mass resolution was maximized at 1,600 Da. Myoglobin digested with trypsin was used to calibrate the mass instrument with internal calibration mode. All acquired spectra of samples were processed using TOF/TOF Explorer™ Software (AB SCIEX, Shanghai, China) in a default mode. The data were searched by GPS Explorer (Version 3.6) with the search engine MASCOT (Version 2.3, Matrix Science Inc., Boston, MA). The search parameters were as follows: viridiplantae database (1,850,050 sequences; 6,42,453,415 residues), trypsin digest with one missing cleavage, MS tolerance was set at 100 ppm, MS/MS tolerance was set at 0.6 Da. At least two peptides were required to match for each protein. Protein identifications were accepted if MASCOT score was not less than 75, and the number of matched peptides was not less than five or the sequence coverage was not less than 20% (Lee et al., 2010; You et al., 2014). Searches were also performed against the *C. sinensis* databases (https://phytozome.jgi.doe.gov/pz/portal.html#!info?alias$=$Org_Csinensis).

Bioinformatics analysis of proteins was performed according to Yang et al. (2013).

### Principal components analysis (PCA) of differentially abundant proteins (DAPs)

The ratios of all the DAPs from B-toxic *C. sinensis* and *C. grandis* roots were normalized and transformed for the PCA using Princomp function in R circumstance. The first two components were selected and used to visualize two loadings against each other to investigate the relationships between the variables (Mardia et al., 1979). The PCA loading plots were carried out in triplicate.

### qRT-PCR analysis

Approximately 300 mg frozen roots collected equally from five seedlings (one seedling per pot) were pooled as one biological replicate. qRT-PCR analysis was run in three biological and two technical replicates for each treatment (total of 15 seedlings from 15 pots) according to Zhou et al. (2013). In this study, we randomly selected ten DAPs from each citrus species for qRT-PCR analysis. A total of 20 DAPs were selected from B-toxic *C. sinensis* and *C. grandis* roots. Specific primers were designed from the corresponding sequences of these selected DAPs in citrus genome (https://phytozome.jgi.doe.gov/pz/portal.html#!info?alias$=$Org_Csinensis) using Primer Primier Version 5.0 (PREMIER Biosoft International, CA, USA). The sequences of the F and R primers used were listed in Table S1. For the normalization of gene expression and reliability of quantitative analysis, two citrus genes [*C. sinensis NADP-dependent glyceraldehyde-3-phosphate dehydrogenase* (*GAPDH*; gi|985455672) and *C. sinensis DNA-directed RNA polymerase II subunit 4* (*RPII*; gi|985473508] were selected as internal standards and the roots from control seedlings were used as reference sample, which was set to 1.

### Assay of S-adenosylmethionine (SAM) synthetase (SAMS) and adenosine kinase (ADK)

Both ADK and SAMS were extracted according to Shen et al. (2002) by homogenizing ca. 100 mg of frozen roots in 1 mL extraction buffer including 100 mM of Tris (pH 7.5), 2 mM of ethylenediaminetetraacetic acid (EDTA), 20% (w/v) of glycerol, 20 mM of β-mercaptoethanol, 1 mM of dithiothreitol (DTT) at 4°C. After centrifugation at 10,000 g for 10 min, the supernatant was used immediately for enzyme assay. There were four replicates per treatment.

Total SAMS activity was assayed as described by Kim et al. (1992) and Shen et al. (2002). Briefly, 135 μL of an enzyme extract was incubated in 0.45 mL of a reaction mixture containing 100 mM of Tris (pH 8.0), 30 mM of MgSO_4_, 10 mM of KCl, 20 mM of ATP, and 5 mM of methionine. Blank contained all reagents except for methionine. Reaction was incubated for 1 h at 25°C and was terminated by adding 0.5 mL of 6% (w/v) sodium dodecyl sulfate (SDS), and the phosphate (Pi) released from the substrate was determined as described by Smith et al. (1984) by adding 0.6 mL of an assay mixture containing 6 parts of 3.6 mM ammonium molybdate in 0.5 M H_2_SO_4_, and 1 part of 10% (w/v) ascorbic acid. The sample was incubated at 37°C for 60 min and the absorbance measured at 820 nm.

Root ADK activity was assayed according to Chen and Eckert (1977) and Lindberg et al. (1967) in 1 mL of a reaction mixture containing 20 mM of Tris-maleate (pH 5.8), 0.7 mM of ATP, 0.25 mM of phosphoenolpyruvate (PEP), 0.2 mM of NADH, 0.5 mM of MgCl_2_, 50 mM of KCl, 0.05 mM of adenosine, 5 U of pyruvate kinase, 5 U of lactate dehydrogenase, and 0.1 mL of enzyme extract. The reaction mixture was always preincubated for 10 min at room temperature (25°C) with all of the regents before starting the reaction by the addition of adenosine.

### Experimental design and statistical analysis

There were 20 pots (40 seedlings) per treatment in a completely randomized design. Experiments were performed with 3–10 replicates. Significant differences among four treatments were analyzed by two (species) × two (B levels) ANOVA and four means were separated by the Duncan's new multiple range test at *P* < 0.05. Significant tests between two means (B-toxicity and control) were performed by unpaired *t*-test at *P* < 0.05 level.

## Results and discussion

### *C. sinensis* was more tolerant to B-toxicity than *C. grandis*

**Figure 1:**
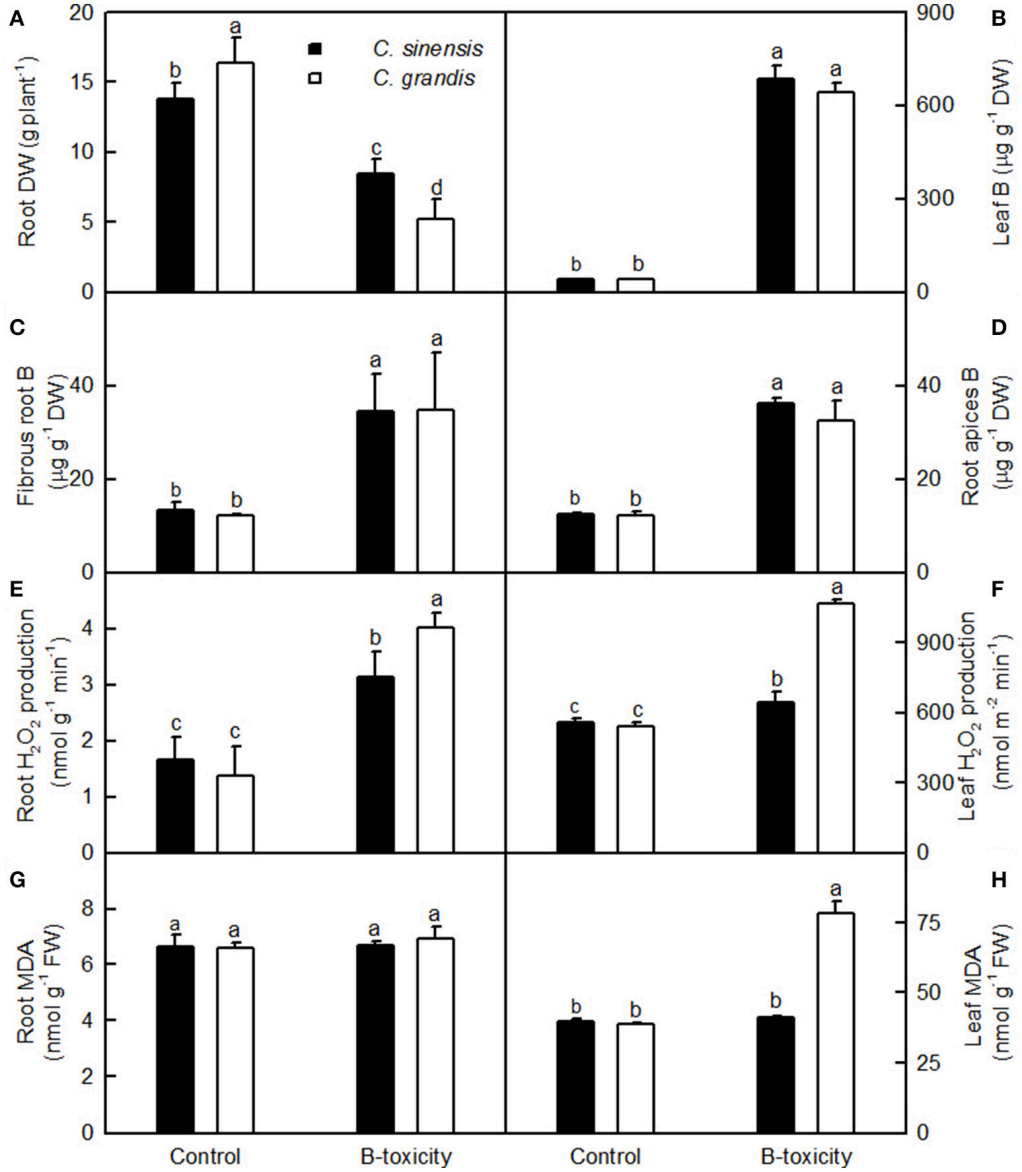
**Effects of B-toxicity on root DW (A), B concentration in leaves (B), fibrous roots (C) and root apices (D), H_2_O_2_ production in roots (E) and leaves (F), and MDA concentration in roots (G) and leaves (H)**. Bars represent means ± SD (*n* = 10 for root DW and 4 for other parameters). Differences among four treatments were analyzed by two (species) × two (B levels) ANOVA. Different letters above the bars indicate a significant difference at *P* < 0.05.

In previous studies, we showed that a concentration of 400 μM B is suitable for the comparative investigation of B-tolerance between B-tolerant *C. sinensis* and B-sensitive *C. grandis*. The typical B-toxic symptoms only occurred in *C. grandis* leaves (Guo et al., 2014; Huang et al., 2014; Sang et al., 2015). We therefore decided to use this B treatment in the present work to reveal specific root proteome signatures in tolerant and sensitive citrus species. As shown in Figures 1A–D, 400 μM B-treatment greatly decreased root DW, increased the concentration of B in leaves, fibrous roots and root apices, and the concentration of B in 400 μM B-treated leaves was far more than the sufficiency range of 30–100 mg kg^−1^ DW for citrus (Chapman, 1968). Thus, seedlings that received 10 and 400 μM B are considered as B-toxic and B-sufficient (control), respectively.

Our results showed that the B-toxicity-induced decrease in root DW (Figure 1A) and increase in H_2_O_2_ production in roots and leaves (Figures 1E,F) were greater in *C. sinensis* seedlings than in *C. grandis* ones, and that B-toxicity increased the concentration of MDA only in *C. grandis* leaves (Figure 1H). In addition, the typical visible B-toxic symptom only occurred in B-toxic *C. grandis* leaves, but was not found in B-toxic *C. sinensis* leaves except for very few seedlings (Figure S1). Previous studies showed that B-toxicity only decreased the concentrations of phosphorus (P) and total soluble proteins in *C. grandis* roots (Guo et al., 2016). Based on these results, we concluded that *C. sinensis* had higher B-tolerance than *C. grandis*.

### Protein yield and DAPs in B-toxic roots

**Figure 2:**
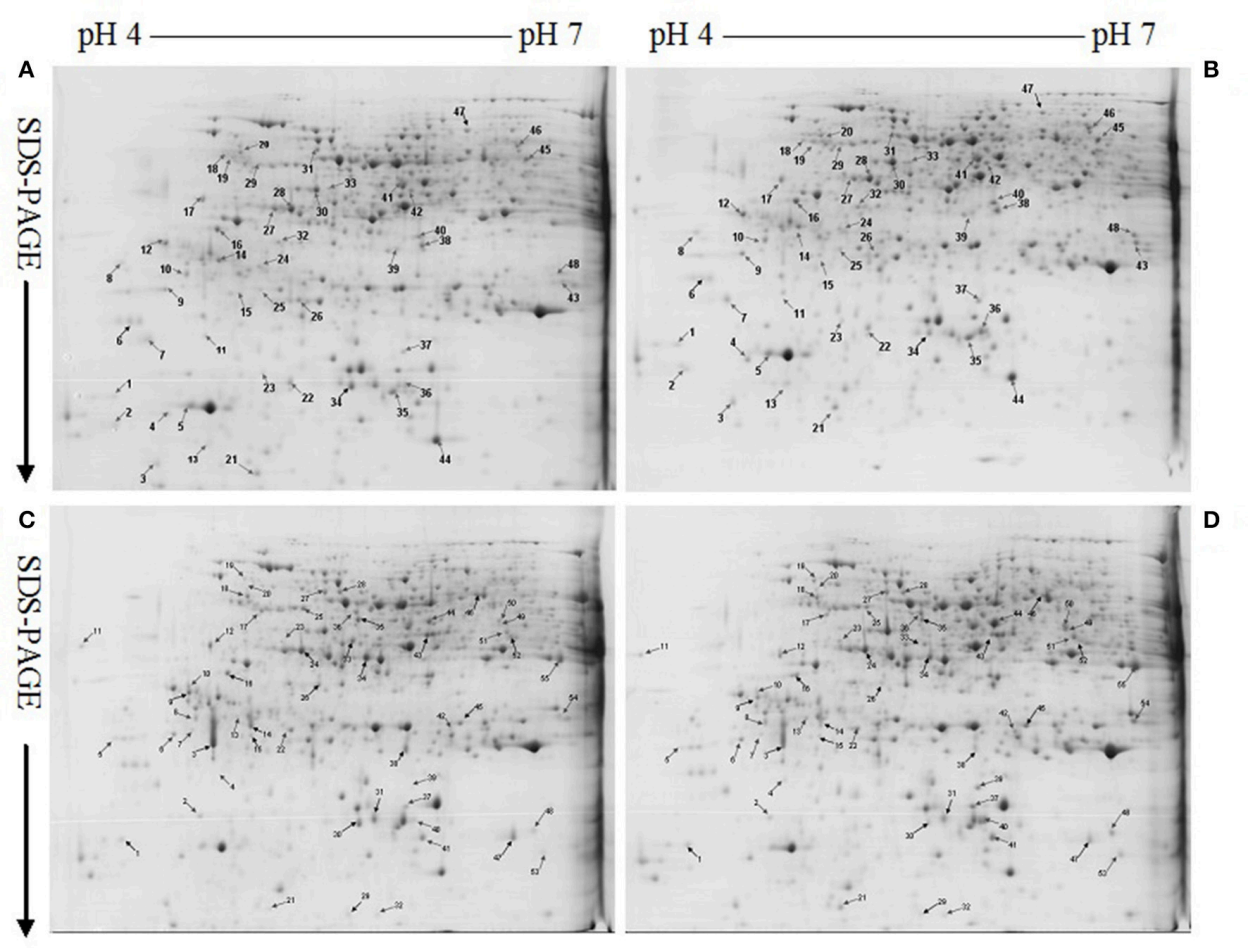
**Representative 2-DE images of proteins extracted from control (A,C) and B-toxic (B,D) roots**. **(A)** Control roots of *Citrus sinensis*, **(B)** B-toxic root of *C. sinensis*, **(C)** Control roots of *Citrus grandis*, **(D)** B-toxic roots of *C. grandis*.

Protein yield did not differ among four treatment combinations (Table 1). After Coomassie Brilliant Blue G-250 staining, more than 800 clear and reproducible protein spots were discovered on each gel. The number of protein spots per gel were similar among the four treatment combinations (Table 1 and Figure 2; Figure S2), as obtained on *C. sinensis* and *C. grandis* leaves (Sang et al., 2015).

**Table 1:** **Protein yield, number of spots, number of variable spots and number of identified differentially abundant protein spots in *Citrus sinensis* and *Citrus grandis* roots**.

	***Citrus sinensis***		***Citrus grandis***	
	**Control**	**B-toxicity**	**Control**	**B-toxicity**
Protein yield (mg g^−1^ FW)	13.30 ± 0.26a	13.25 ± 0.15a	11.26 ± 0.39a	10.52 ± 0.18a
Number of spots per gel	821 ± 41a	824 ± 31a	833 ± 40a	818 ± 27a
**NUMBER OF VARIABLE SPOTS**				
Increase in relative abundance		43		35
Decrease in relative abundance		5		20
Total		48		55
**NUMBER OF IDENTIFIED DIFFERENTIALLY ABUNDANT PROTEIN SPOTS**				
Increase in relative abundance		27		28
Decrease in relative abundance		4		13
Total		31		41

*Data for protein yield and number of spots per gel are the mean ± SD (n = 3). Different letters within a row indicate significant differences at P < 0.05*.

**Figure 3:**
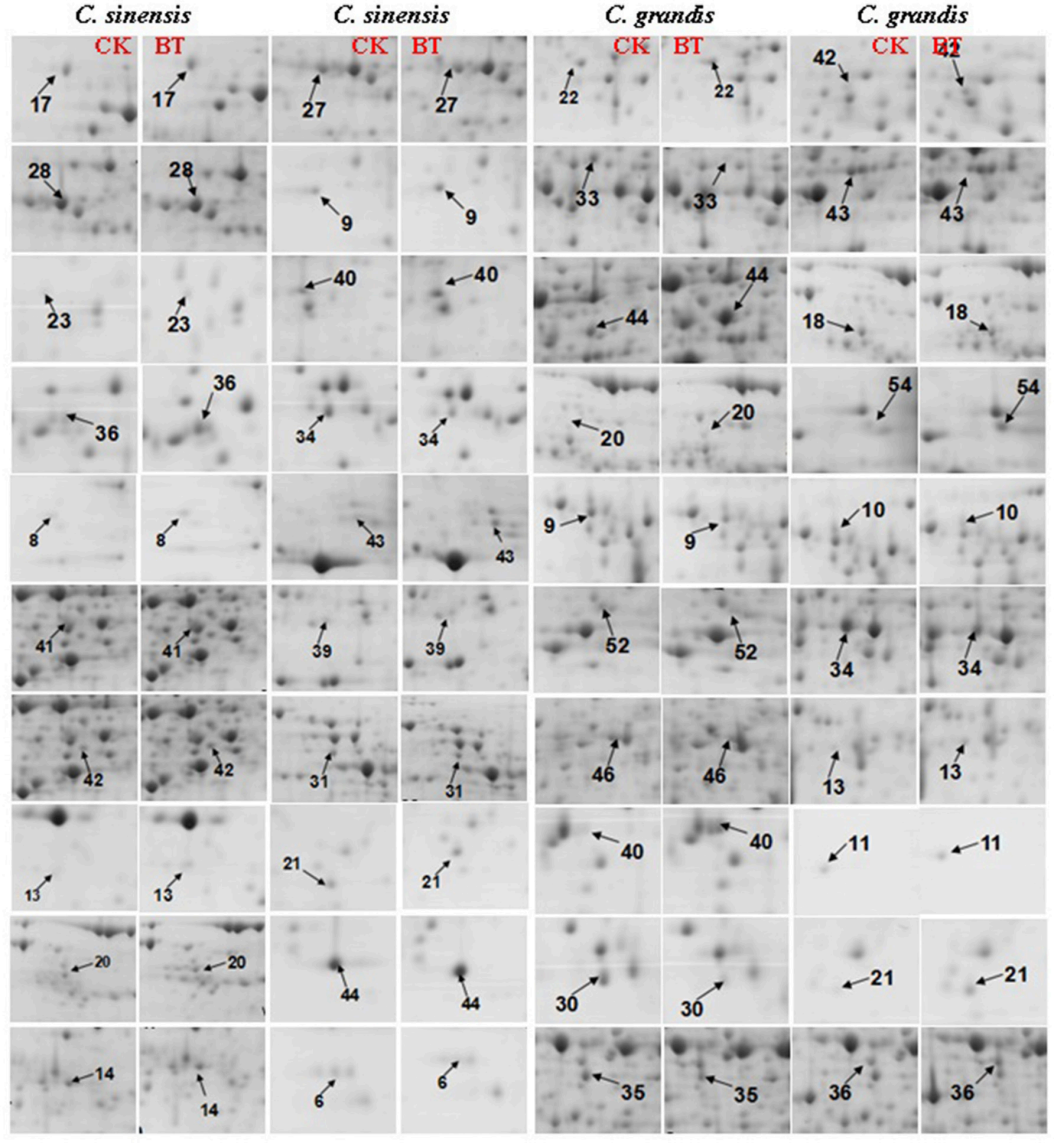
**Close-up views of the differentially abundant protein spots in control (CK) and B-toxic (BT) roots**.

**Figure 4:**
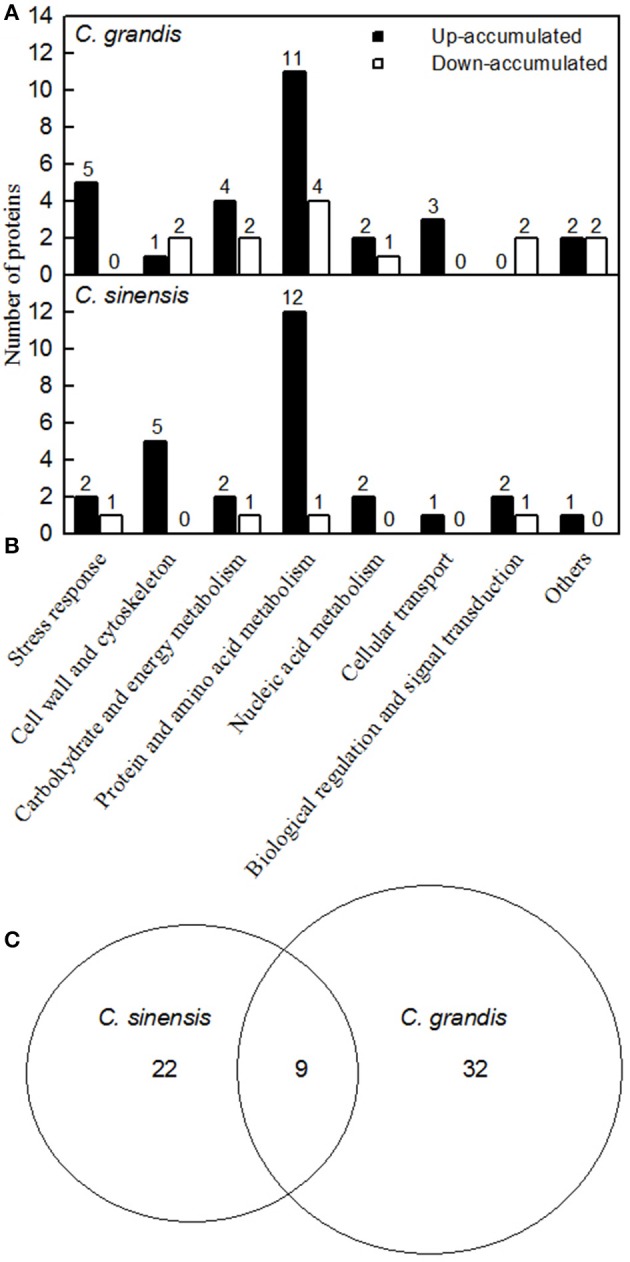
**Classification of B-toxicity-responsive protein spots in *C. grandis* (A), *C. sinensis* (B) roots, and venn diagram analysis of B-toxicity-responsive protein spots in citrus roots (C)**.

We detected 43 up- and five down-accumulated, and 35 up- and 20 down-accumulated protein spots from B-toxic *C. sinensis* and *C. grandis* roots, respectively. Twenty-seven up- and four down-accumulated, and 28 up- and 13 down-accumulated protein spots were identified from B-toxic *C. sinensis* and *C. grandis* roots, respectively after these differentially accumulated protein spots being submitted to the MALDI-TOF/TOF-MS-based identification (Table 1, Figures 2, 3 and Tables S2–S5). These DAPs were mainly involved in protein and amino acid metabolism, stress response, cell wall and cytoskeleton metabolism, carbohydrate and energy metabolism, nucleic acid metabolism, cellular transport, and biological regulation and signal transduction (Tables 2, 3 and Figures 4A,B). Most of B-toxicity-responsive proteins were isolated from B-toxic *C. sinensis* or *C. grandis* roots, only nine protein species with the same accession No. were shared by the both. Among the nine overlapping proteins, only five proteins displayed similar change trends in B-toxic *C. sinensis* and *C. grandis* roots (Tables 2, 3 and Figure 4C). These results demonstrated that B-toxicity-responsive proteins greatly differed between *C. sinensis* and *C. grandis* roots, as obtained on B-toxic *C. sinensis* and *C. grandis* leaves (Sang et al., 2015).

**Table 2:** **Differentially abundant proteins and their identification by MALDI-TOF/TOF-MS in B-toxic *Citrus sinensis* roots**.

**Spot No**.	**Protein identity**	**Accession no**.	**Mr(kDa)/pI Theor**.	**Mr (kDa)/pI Exp**.	**Reference species**	**Protein score**	**NMP**	**Ratio**	**Covered sequence (%)**	**Charge**
**STRESS RESPONSE**										
**S16**	**Heat shock protein 83**	**gi|169296**	**80.77/4.95**	**81.1/5.0**	***Ipomoea nil***	**102**	**8**	**0.42** ± **0.20**	**14**	**1**
S32	Mitochondrial chaperonin hsp60	gi|20466256	61.242/5.66	60.3/5.18	*Arabidopsis thaliana*	288	22	1.92 ± 0.31	29	1
S17	Late-embryogenesis abundant protein 2	gi|212552206	34.343/4.72	35.2/4.53	*Glycine max*	260	14	1.87 ± 0.31	36	1
**CELL WALL AND CYTOSKELETON**										
S30	Actin	gi|6103623	41.564/5.30	42.6/5.81	*Picea rubens*	637	25	1.63 ± 0.10	50	1
S29	Alpha-tubulin	gi|321437427	46.368/4.90	46.2/4.8	*Musa acuminata AAA Group*	647	21	1.70 ± 0.26	47	1
S18	Beta-tubulin 14	gi|166343835	49.98/4.76	50.4/4.6	*Gossypium hirsutum*	618	31	2.97 ± 0.20	40	1
S19	Tubulin β-1 chain	gi|332197637	50.185/4.68	51.6/4.91	*Arabidopsis thaliana*	660	27	2.19 ± 0.46	33	1
S3	Profilin	gi|12659206	14.133/4.90	13.8/4.94	*Corylus avellana*	89	9	1.57 ± 0.04	43	1
**CARBOHYDRATE AND ENERGY METABOLISM**										
S27	Adenosine kinase 2, partial	gi|149391003	26.477/4.88	25.9/5.63	*Oryza sativa Indica Group*	80	7	1.82 ± 0.15	21	1
**S28**	**Adenosine kinase isoform 1T-like protein**	**gi|82400168**	**37.549/5.01**	**38.6/5.71**	***Solanum tuberosum***	**113**	**6**	**1.81** ± 0.14	**20**	**1**
S47	Malate dehydrogenase, partial	gi|160690776	14.017/7.03	15.4/5.05	*Citrus trifoliata*	220	6	0.50 ± 0.09	38	1
**PROTEIN AND AMINO ACID METABOLISM**										
**S9**	**Proteasome subunit alpha type, putative**	**gi|255584432**	**27.0/4.73**	**27.8/4.75**	***Ricinus communis***	**191**	**11**	**1.76** ± **0.12**	**42**	**1**
S12	Elongation factor 1-delta 1	gi|226505926	24.767/4.39	23.8/4.38	*Zea mays*	219	11	2.41 ± 0.31	20	1
S15	Elongation factor 2	gi|195646972	93.862/6.00	94.2/6.51	*Zea mays*	143	8	2.90 ± 0.21	9	1
S23	Translation initiation factor	gi|197312901	16.419/4.98	16.2/5.12	*Rheum australe*	188	7	1.72 ± 0.08	26	1
S40	Eukaryotic translation initiation factor 2 beta subunit-like	gi|82621136	29.831/6.08	30.6/6.54	*Solanum tuberosum*	101	9	1.85 ± 0.15	32	1
**S36**	**Eukaryotic translation initiation factor 5A1**	**gi|217038830**	**17.387/5.60**	**17.2/5.7**	***Glycine max***	**153**	**10**	**3.13** ± 0.72	**28**	**1**
**S34**	**Eukaryotic initiation factor 5A (2)**	**gi|19702**	**17.353/5.60**	**18.5/6.4**	***Nicotiana plumbaginifolia***	**120**	**10**	**0.48** ± 0.09	**58**	**1**
S8	Alpha chain of nascent polypeptide associated complex	gi|124484511	21.911/4.32	22.1/4.52	*Nicotiana benthamiana*	242	8	2.03 ± 0.37	20	1
S43	Alanine aminotransferase 2	gi|332197185	47.68/6.32	48.2/6.61	*Arabidopsis thaliana*	138	5	2.28 ± 0.30	11	1
S41	S-adenosylmethionine synthase 2	gi|1655578	42.977/5.51	43.3/5.93	*Catharanthus roseus*	241	19	1.89 ± 0.14	47	1
S39	5-methyltetrahy dropteroyltriglutamate-homocysteine methyltransferase, putative	gi|255569484	84.668/6.09	85.6/6.52	*Ricinus communis*	373	19	1.97 ± 0.16	27	1
S42	Transaminase mtnE, putative	gi|255562088	50.396/6.95	51.8/6.05	*Ricinus communis*	244	10	1.74 ± 0.16	22	1
**S31**	**Ketol-acid reductoisomerase**	**gi|295291644**	**63.583/6.49**	**64.6/6.62**	***Catharanthus roseus***	**286**	**16**	**2.07** ± 0.38	**21**	**1**
**NUCLEIC ACID METABOLISM**										
S13	Glycine-rich RNA-binding protein 4	gi|332643299	14.12/5.03	15.6/8.68	*Arabidopsis thaliana*	133	4	1.85 ± 0.13	33	1
**S21**	**Glycine-rich RNA-binding protein**	**gi|7024451**	**16.839/4.98**	**17.4/7.85**	***Citrus unshiu***	**227**	**10**	**1.92** ± **0.31**	**28**	**1**
**CELLULAR TRANSPORT**										
**S20**	**Vacuolar H**^+^**-ATPase B subunit**	**gi|4519264**	**54.329/4.91**	**55.6/4.91**	***Citrus unshiu***	**641**	**35**	**1.67** ± **0.15**	**53**	**1**
**BIOLOGICAL REGULATION AND SIGNAL TRANSDUCTION**										
S44	Nucleoside diphosphate kinase 1	gi|255571035	176.41/5.09	179.1/6.3	*Ricinus communis*	136	4	1.51 ± 0.16	20	1
S14	14-3-3 family protein	gi|291162645	29.404/4.74	30.5/4.71	*Dimocarpus longan*	241	7	1.52 ± 0.14	33	1
S6	Translationally controlled tumor-like protein	gi|115187479	19.116/4.54	20.0/4.7	*Arachis hypogaea*	286	9	0.44 ± 0.14	31	1
**OTHER**										
**S37**	**Unnamed protein product, partial**	**gi|296088008**	**17.163/5.21**	**18.2/7.93**	***Vitis vinifera***	**709**	**17**	**1.84** ± **0.27**	**51**	**1**

*Spot number corresponds to the 2-DE gel imagines in Figures 2A,B. NMP means the number of peptides. Ratio means the ratio of B-toxic roots to controls and the values were the means ± SE of three replicates. Covered sequence (%) means the ratio of the number of amino acids of the matched peptides to the number of amino acids of the full-length protein. Proteins shared by C. sinensis and C. grandis roots were marked in bold*.

**Table 3:** **Differentially abundant proteins and their identification by MALDI-TOF/TOF-MS in B-toxic *Citrus grandis* roots**.

**Spot No**.	**Protein identity**	**Accession No**.	**Mr(kDa)/pI Theor**.	**Mr (kDa)/pI Exp**.	**Reference species**	**Protein score**	**NMP**	**Ratio**	**Covered sequence (%)**	**Charge**
**STRESS RESPONSE**										
G32	Cu/Zn superoxide dismutase, partial	gi|2274917	12.784/5.82	13.5/5.61	*Citrus sinensis*	179	7	1.91 ± 0.11	52	1
G26	Lactoylglutathione lyase, putative	gi|255554865	31.547/5.11	31.8/7.63	*Ricinus communis*	219	12	1.85 ± 0.18	40	1
**G16**	**Heat shock protein 83**	**gi|169296**	**80.820/4.95**	**81.4/5.12**	***Ipomoea nil***	**102**	**8**	**1.60** ± **0.17**	**14**	**1**
G19	60-kDa chaperonin-60 alpha -polypeptide precursor, partial	gi|289365	57.692/4.84	58.4/4.58	*Brassica napus*	435	26	1.92 ± 0.15	41	1
G12	Chilling-responsive protein	gi|153793260	35.739/4.85	36.4/4.6	*Nicotiana tabacum*	284	9	1.88 ± 0.27	12	1
**CELL WALL AND CYTOSKELETON**										
G52	Alpha-1,4-glucan-protein synthase 1	gi|195623832	40.905/6.60	40.2/6.21	*Zea mays*	422	20	0.48 ± 0.12	49	1
G34	Actin 1	gi|255115691	41.665/5.31	41.9/5.14	*Boehmeria nivea*	169	12	0.40 ± 0.10	30	1
G25	Alpha-tubulin	gi|334261583	49.446/4.99	50.3/5.0	*Pellia endiviifolia*	297	12	2.02 ± 0.30	26	1
**CARBOHYDRATE AND ENERGY METABOLISM**										
G46	ATP synthase subunit α	gi|222356608	40.289/8.59	41.6/8.9	*Afrothismia hydra*	323	13	0.47 ± 0.15	29	1
**G24**	**Adenosine kinase isoform 1T-like protein**	**gi|82400168**	**37.572/5.01**	**37.2/5.16**	***Solanum tuberosum***	**113**	**6**	**0.49** ± **0.10**	**20**	**1**
G22	Triosephosphate isomerase	gi|295687231	33.119/6.66	33.6/5.94	*Gossypium hirsutum*	316	12	2.17 ± 0.12	25	1
G42	Triosphosphate isomerase-like protein	gi|76573375	27.711/5.88	28.3/5.9	*Solanum tuberosum*	215	8	2.02 ± 0.17	23	1
G51	Phosphoglycerate kinase	gi|332198142	42.131/5.49	43.2/5.61	*Arabidopsis thaliana*	120	9	1.74 ± 0.24	15	1
G50	Dihydrolipoyllysine-residue succinyltransferase component of 2-oxoglutarate dehydrogenase complex	gi|226509380	48.749/5.11	49.4/5.64	*Zea mays*	92	12	1.56 ± 0.16	51	1
**PROTEIN AND AMINO ACID METABOLISM**										
**G6**	**Proteasome subunit alpha type, putative**	**gi|255584432**	**27.017/4.73**	**27.8/4.75**	***Ricinus communis***	**191**	**11**	**1.77** ± 0.16	**42**	**1**
G13	26S proteasome subunit RPN12	gi|32700048	30.701/4.81	31.5/4.66	*Arabidopsis thaliana*	218	9	1.91 ± 0.18	29	1
G48	Ubiquitin-conjugating enzyme variant	gi|257196367	16.630/6.20	17.6/6.6	*Citrus sinensis*	438	22	1.63 ± 0.32	80	1
G53	Ubiquitin-conjugating enzyme E2 35	gi|332198044	17.191/6.74	18.3/6.41	*Arabidopsis thaliana*	387	14	2.97 ± 0.70	47	1
G29	Polyubiquitin, partial	gi|284927592	11.992/8.2	12.3/5.12	*Citrus sinensis*	389	11	0.31 ± 0.05	66	1
G7	Translation initiation factor IF6	gi|332645889	26.482/4.63	27.8/4.52	*Arabidopsis thaliana*	246	6	1.74 ± 0.07	16	1
G40	Eukaryotic translation initiation factor 5A isoform VII	gi|33325129	17.471/5.60	18.2/5.9	*Hevea brasiliensis*	121	10	4.75 ± 1.19	40	1
**G11**	**Eukaryotic translation initiation factor 5A1**	**gi|217038830**	**17.397/5.60**	**17.9/5.58**	***Glycine max***	**239**	**13**	**2.27** ± 0.44	**32**	**1**
**G41**	**Eukaryotic translation initiation factor 5A1**	**gi|217038830**	**17.397/5.60**	**18.4/5.6**	***Glycine max***	**236**	**10**	**1.55** ± **0.12**	**65**	**1**
**G30**	**Eukaryotic initiation factor 5A (2)**	**gi|19702**	**17.363/5.60**	**18.7/5.52**	***Nicotiana plumbaginifolia***	**120**	**10**	**0.36** ± **0.06**	**58**	**1**
G33	S-adenosylmethionine synthetase 1 family protein	gi|222861722	43.213/5.68	43.5/5.82	*Populus trichocarpa*	598	18	0.43 ± 0.06	39	1
G43	S-adenosylmethionine synthetase 1 family protein	gi|222861722	43.213/5.68	44.1/5.73	*Populus trichocarpa*	677	17	0.44 ± 0.04	45	1
G44	S-adenosylmethionine synthetase	gi|14600072	43.184/5.67	43.8/5.52	*Brassica juncea*	490	23	2.01 ± 0.26	33	1
**G27**	**Ketol-acid reductoisomerase**	**gi|295291644**	**63.623/6.49**	**64.2/6.81**	***Catharanthus roseus***	**286**	**16**	**1.68** ± **0.04**	**21**	**1**
**G28**	**Ketol-acid reductoisomerase**	**gi|295291644**	**63.623/6.49**	**65.1/6.9**	***Catharanthus roseus***	**332**	**16**	**1.81** ± **0.02**	**16**	**1**
**NUCLEIC ACID METABOLISM**										
**G21**	**Glycine-rich RNA-binding protein**	**gi|7024451**	**16.848/7.85**	**17.3/4.98**	***Citrus unshiu***	**227**	**10**	**2.64** ± **0.16**	**28**	**1**
G35	DEAD-box RNA helicase-like protein	gi|283049402	46.935/5.48	47.2/5.6	*Prunus persica*	735	33	0.42 ± 0.17	50	1
G36	Spliceosome RNA helicase BAT1	gi|226528292	45.146/6.03	48.1/6.52	*Zea mays*	434	24	1.97 ± 0.33	41	1
**CELLULAR TRANSPORT**										
**G18**	**Vacuolar H**^+^**-ATPase B subunit**	**gi|4519264**	**54.362/4.91**	**55.2/5.02**	***Citrus unshiu***	**641**	**35**	**1.80** ± **0.20**	**53**	**1**
**G20**	**Vacuolar H**^+^**-ATPase B subunit**	**gi|4519264**	**54.362/4.91**	**54.9/5.1**	***Citrus unshiu***	**121**	**13**	**1.80** ± **0.16**	**15**	**1**
G54	GTP-binding nuclear protein Ran-A1	gi|192913008	24.9/6.38	25.8/6.51	*Elaeis guineensis*	86	8	2.24 ± 0.03	28	1
**BIOLOGICAL REGULATION AND SIGNAL TRANSDUCTION**										
G9	14-3-3-like protein GF14 phi	gi|332193639	30.193/4.79	31.5/4.63	*Arabidopsis thaliana*	483	22	0.48 ± 0.19	53	1
G10	14-3-3-like protein GF14 phi	gi|332193639	30.193/4.79	29.8/5.12	*Arabidopsis thaliana*	436	21	0.41 ± 0.16	54	1
**OTHERS**										
G15	12-oxo-phytodienoic acid reductase2	gi|162459589	41.665/6.08	42.4/6.31	*Zea mays*	110	11	0.45 ± 0.04	31	1
G49	12-oxo-phytodienoic acid reductase2	gi|162459589	41.665/6.08	42.8/6.25	*Zea mays*	98	11	0.49 ± 0.14	31	1
**G2**	**Unnamed protein product, partial**	**gi|296088008**	**17.173/7.93**	**17.8/5.16**	***Vitis vinifera***	**503**	**13**	**1.69** ± **0.09**	**35**	**1**
**G39**	**Unnamed protein product, partial**	**gi|296088008**	**17.173/7.93**	**17.2/5.21**	***Vitis vinifera***	**709**	**17**	**3.63** ± **0.72**	**56**	**1**

*Spot number corresponds to the 2-DE imagines in Figures 2C,D. NMP means the number of peptides. Ratio means the ratio of B-toxic roots to controls and the values were the means ± SE of three replicates. Covered sequence (%) means the ratio of the number of amino acids of the matched peptides to the number of amino acids of the full-length protein. Proteins shared by C. sinensis and C. grandis roots were marked in bold*.

### Principal component analysis loading plots of DAPs

**Figure 5:**
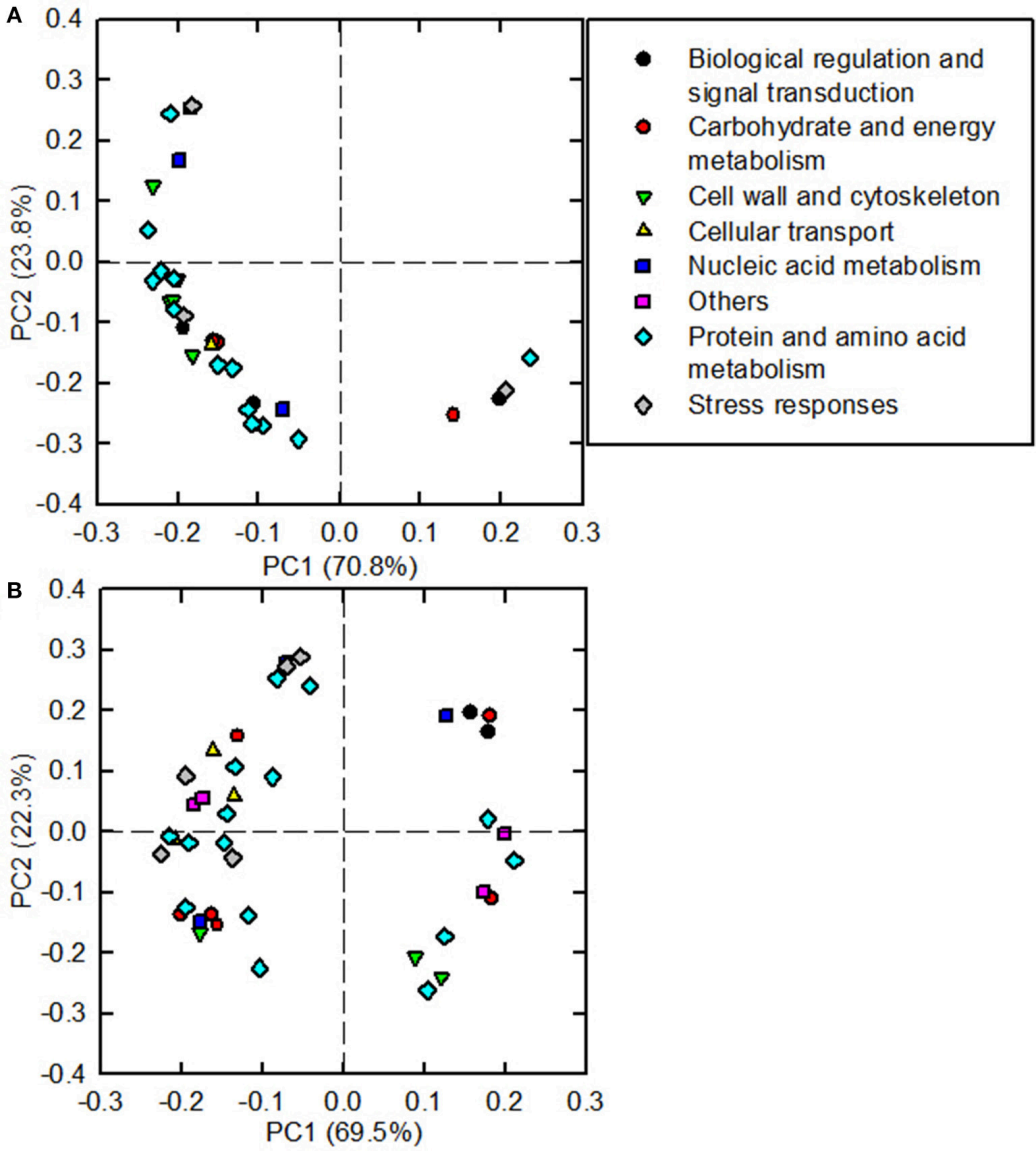
**Principal component analysis (PCA) loading plots of differentially abundant proteins in B-toxic *C. sinensis* (A) and *C. grandis* (B) roots**.

As shown in Figure 5, 31 and 41 B-toxicity-responsive proteins identified in *C. sinensis* and *C. grandis* roots were submitted to PCA procedure. The first two components accounted for 94.6% (70.8% for PC1 and 23.8% for PC2) and 91.8% (69.5% for PC1 and 22.3% for PC2) of total variation in *C. sinensis* and *C. grandis* roots, respectively. The DAPs involved in protein and amino acid metabolism and cell wall and cytoskeleton were highly clustered in *C. sinensis* roots. In contrast, no obvious clustered proteins were observed in *C. grandis* roots.

### qRT-PCR analysis of genes for DAPs

**Figure 6:**
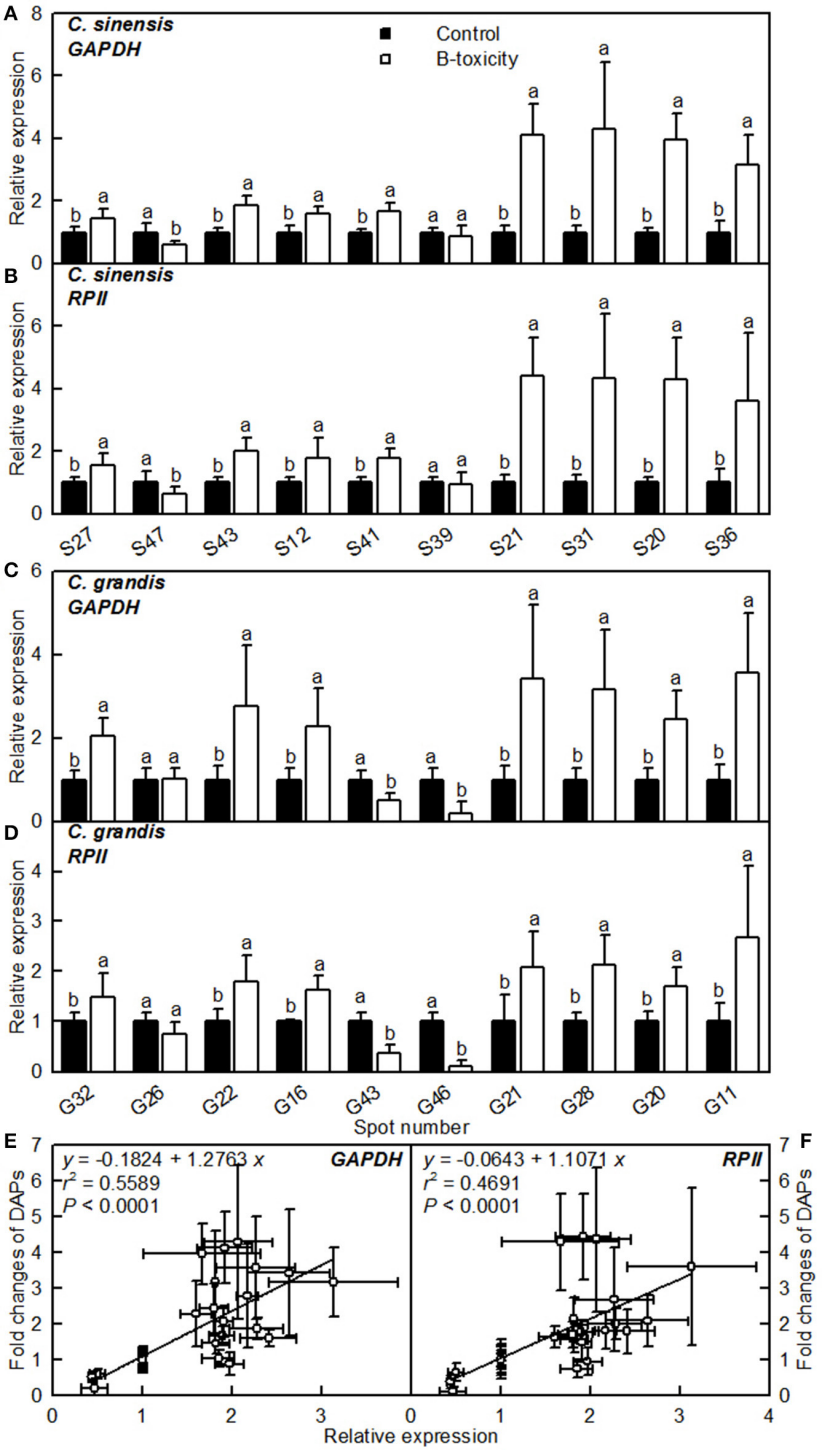
**Relative expression levels of genes encoding 20 B-toxicity-responsive proteins from *C. sinensis* (A,B) and *C. grandis* (C,D) roots using *GAPDH* (A,C) and *RPII* (B,D) as internal standards, and the correlation analysis of qRT-PCR results and 2-DE data (E,F)**. For **(A–D)**, bars represent means ± SD (*n* = 3); Significant tests between two means were performed by unpaired *t*-test; Different letters above the bars indicate a significant difference at *P* < 0.05. For **(E,F)**, 2-DA data from Tables 2, 3.

The mRNA levels of genes encoding 20 B-toxicity-responsive proteins from *C. sinensis* (S27, 47, 43, 12, 41, 39, 13, 31, 20, and 36) and *C. grandis* (G32, 26, 22, 16, 43, 46, 21, 28, 20, and 11) roots (Figures 6A–D) were assayed in order to examine the relationship between the abundances of proteins and the expression levels of genes. The expression levels of all genes except for S39 and G26 matched well with our 2-DE data (Tables 2, 3) regardless of which internal standard was used to calculate the relative expression levels, suggesting that most of B-toxicity-responsive proteins were regulated at the transcriptional level. This is also supported by our analysis that the qRT-PCR data and the 2-DE results were significantly and positively correlated (Figures 6E,F).

### Proteins related to carbohydrate and energy metabolism

**Figure 7:**
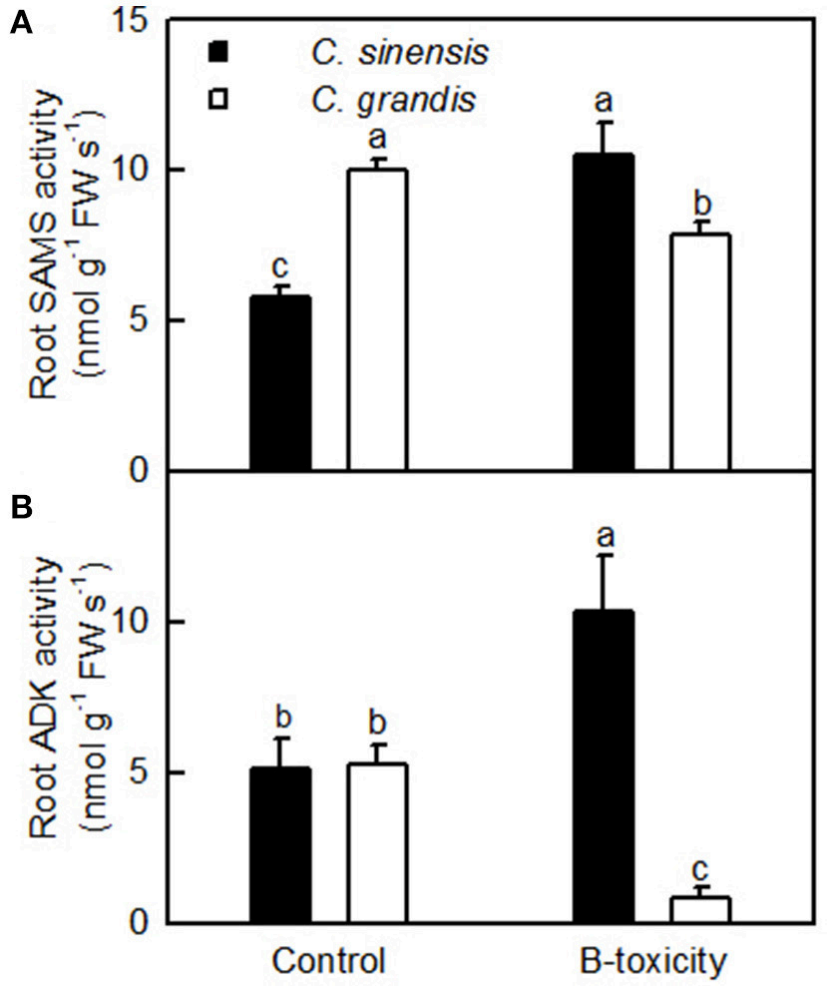
**Effects of B-toxicity on the activities of SAMS (A)** and ADK **(B)** in *C. sinensis* and *C. grandis* roots.Bars represent means ± SD (*n* = 4). Differences among four treatments were analyzed by two (species) × two (B levels) ANOVA. Different letters above the bars indicate a significant difference at *P* < 0.05.

ADK plays key roles in the maintenance of purine nucleotide pools and in the active methyl cycle (Moffatt et al., 2002; Kettles et al., 2014; Chen et al., 2015). Schoor et al. (2011) observed that silencing of *ADK* in *Arabidopsis* caused impaired root growth, small, crinkled rosette leaves, and decreased apical dominance accompanied by an increased concentration of active cytokinin (CK) ribosides, concluding that ADK was responsible for CK homeostasis *in vivo*. We found that the abundances of ADK2 (S27) and ADK isoform 1T-like protein (S28) were increased in B-toxic *C. sinensis* roots, while only one down-accumulated ADK isoform 1T-like protein (G24) was identified in B-toxic *C. grandis* roots (Tables 2, 3). Similarly, B-toxicity increased the abundances of S-adenosylmethionine synthetase 2 [also known as S-adenosyl-L-methionine synthetase 2 (SAMS2); S41] involved in the formation of SAM from methionine and ATP, and 5-methyltetrahydropteroyltriglutamate-homocysteine methyltransferase (also known as methionine synthase; S39) involved in the biosynthesis of methionine in *C. sinensis* roots and SAMS (G44) in *C. grandis* roots, and decreased the abundances of two SAMS1 family protein (G33 and 43) in *C. grandis* roots (Tables 2, 3). In addition, the activities of both SAMS and ADK were increased in B-toxic *C. sinensis* roots, but decreased in B-toxic *C. grandis* roots (Figure 7). Therefore, the active methyl cycle might be upregulated in B-toxic *C. sinensis* roots, but downregulated in B-toxic *C. grandis* roots. This agrees with the report that the active methyl cycle was induced by drought in drought-resistant rice leaves, but inhibited in drought sensitive rice leaves, and that the cycle played a role in rice drought resistance (Zhang et al., 2012). It is known that SAM not only plays a role in the active methyl cycle but also serves as an intermediate in the biosynthesis of polyamines (PAs) and ethylene (Ravanel et al., 1998). Hassan et al. (2010) reported that the expression of genes encoding SAM decarboxylase [SAMDC, a key enzyme involved in the biosynthesis of PAs (spermidine and spermine)], methinnine synthase 1 and SAMS2 was upregulated in B-tolerant Sahara barley roots, and that an antioxidant mechanisms involving PAs and water-water cycle in Sahara barley might play a role in tolerating high level of soil B. Hassan et al. (2010) also suggested that increased activity of SAMDC on SAM might inhibit ethylene production, hence reducing leaf senescence in Sahara barley. Evidence shows that transgenic plants with elevated levels of PAs have enhanced tolerance to different abiotic stresses (Alcázar et al., 2006). Recently, Tanou et al. (2014) observed that exogenous PAs partially alleviated the NaCl-induced phenotypic and physiological impairments in citrus plants, and systematically upregulated the expression of genes involved in PA biosynthesis (*arginine decarboxylase, SAMDC, spermidine synthase*, and *spermine synthase*) and catabolism (*diamine oxidase and polyamine oxidase*). Also, PAs reprogrammed the oxidative status in salt-stressed citrus plants. Based on these results, we concluded that the B-toxicity-induced upregulation of the active methyl cycle might play a role in the B-tolerance of *C. sinensis via* enhancing the biosynthesis of PAs.

We found that the abundances of all the four B-toxicity-responsive proteins involved in glycolysis (G22, 42, and 51) and tricarboxylic acid (TCA) cycle (G50) were increased in *C. grandis* roots and that ATP synthase subunit α (G46) was down-accumulated in B-toxic *C. grandis* roots (Table 3). Thus, ATP synthase-mediated ATP biosynthesis might be decreased in these roots. This might contribute to the maintenance of ATP balance, when the production of ATP was increased due to upregulated glycolysis and TCA cycle and the consumption of ATP was decreased due to decreased activities of ADK and SAMS. However, the abundance of malate dehydrogenase (MDH, S47) involved in TCA cycle was decreased in B-toxic *C. sinensis* roots (Table 2).

### Stress response-related proteins

Because the production of ROS (H_2_O_2_) was increased in B-toxic *C. grandis* and *C. sinensis* roots, especially in the former (Figure 1E), antioxidant enzymes might be induced in these roots. As expected, the abundance of Cu/Zn-SOD (G32) was increased in B-toxic *C. grandis* roots (Table 3). Besides antioxidant enzymes, the abundance of lactoylglutathione lyase (LGL; G26) was augmented in B-toxic *C. grandis* roots. In addition to the detoxification of methylglyoxal, a cytotoxic compound formed spontaneously from the glycolysis and photosynthesis intermediates glyceraldehyde-3-phosphate and dihydroxyacetone phosphate, LGL also play a role in oxidative stress tolerance (Yadav et al., 2005). The increased abundance of LGL agrees with our data that the abundances of four protein species involved in glycolysis (G22, 42, and 51) and TCA cycle (G50) were elevated in B-toxic *C. grandis* roots. By contrast, we only obtained one up-accumulated late-embryogenesis abundant protein 2 (LEA-2; S17) from B-toxic *C. sinensis* roots (Tables 2, 3). To conclude, more proteins related to detoxification were up-accumulated in B-toxic *C. grandis* roots than in B-toxic *C. sinensis* roots, which agrees with the increased requirement for detoxification of the more ROS and other toxic compounds such as aldehydes in the former because the production of ROS was higher in B-toxic *C. grandis* than in B-toxic *C. sinensis* roots (Figure 1E). We found that the level of MDA did not differ between B-toxic roots and controls (Figure 1C), demonstrating that the upregulation of antioxidant system provided sufficient protection to B-toxic roots against oxidative damage.

### Proteins related to cell wall and cytoskeleton

All of the identified DAPs in cytokeleton were up-accumulated in B-toxic *C. sinensis* roots, while we isolated two down-accumulated proteins in cytokeleton (actin 1, G34) and polysaccharide biosynthesis (α-1,4-glucan-protein synthase 1; G52), and one up-accumulated α-tubulin in cytokeleton (G25) from B-toxic *C. grandis* roots (Tables 2, 3). Thus, *C. sinensis* roots might have a better capacity to keep cytoskeleton and cell wall integrity than *C. grandis* roots under B-toxicity, which might be responsible for the higher B-tolerance of the former. Similar results have been obtained on B-toxic *C. sinensis* and *C. grandis* leaves (Sang et al., 2015).

### Proteins related to protein, amino acid, and nucleic acid metabolisms

Proteasomes are responsible for the degradation of the inactive and futile proteins. Most of proteins degraded by proteasomes are first tagged by ubiquitin (Kurepa and Smalle, 2008). We obtained two up-accumulated proteasomes (G6 and 13) and two up-accumulated ubiquitin-conjugating enzymes (G48 and 54) from B-toxic C. *grandis* roots, but only one up-accumulated proteasome (S9) from B-toxic *C. sinensis* (Tables 2, 3), demonstrating that B-toxicity accelerated proteolysis, especially in the former. This agrees with our data that B-toxicity only decreased total soluble protein concentration in B-toxic *C. grandis* roots (Guo et al., 2016). B-toxicity-induced increase in protein degradation implies that misfolded and damaged proteins were increased in B-toxic *C. sinensis* and *C. grandis* roots, especially in the latter. In addition, we identified one up-accumulated α chain of nascent polypeptide associated complex (α-NAC, S8) from B-toxic *C. sinensis* roots. NAC, including α and β subunits, plays a role in protecting newly synthesized polypeptides on ribosome from proteolysis and in facilitating its folding (Karan and Subudhi, 2012; Kogan and Gvozdev, 2014). Thus, the up-accumulation of α-NAC in B-toxic *C. sinensis* might alleviate B-toxicity induced protein degradation and misfolding, hence preventing the reduction of proteins. All B-toxicity-responsive proteins (S23, 40, and 36, and G7, 40, 11, and 41) in protein biosynthesis were up-accumulated in *C. sinensis* and *C. grandis* roots except for eukaryotic initiation factor 5A (S34 and G30) (Tables 2, 3). Therefore, the lower level of total soluble proteins in B-toxic *C. grandis* roots might be mainly caused by increased proteolysis rather than by decreased biosynthesis.

As shown in Table 2 and Figure 7A, the abundances of the five DAPs (S43, 41, 39, 42, and 31) involved in amino acid metabolism and the activity of SAMS were increased in B-toxic *C. sinensis* roots, suggesting that the biosynthesis of some amino acids might be enhanced in these roots. SAM is an allosteric activator of threonine synthase (TS), which is involved in the biosynthesis of branched chain amino acids (BCAAs, valine, leucine and isoleucine; Curien et al., 1998; Ravanel et al., 1998). Thus, TS might be activated in B-toxic *C. sinensis* roots due to increased SAM biosynthesis resulting from enhanced SAMS activity. Zeh et al. (2001) found that antisense inhibition of *TS* led to increased level of methionine in transgenic potato plants because of the redirection of carbon flow from the threonine to the methionine branch. Ketol-acid reductoisomerase (KARI) is involved in BCAA biosynthesis. Kochevenko and Fernie (2011) reported that the concentrations of BCAAs in the leaves of transgenic tomato line 7, in which the *KARI* transcript level was remained at about 70% of the wildtype level, were not lower than in the wildtype leaves, whereas BCAA levels in the leaves of transgenic lines 3 and 6, in which the expression level of *KARI* was decreased to 27% of the wild-type level, were only 49–79% of the wildtype leaves. Thus, the levels of BCAAs might be enhanced in B-toxic *C. sinensis* roots due to the activation of TS resulting from increased SAMS activity (Figure 7A) and the increased abundances of KARI (Table 2). However, we obtained two down-accumulated SAMS1 (G33 and 43), one up-accumulated SAMS (G44), and two up-accumulated KARI spots (G27 and 28) from B-toxic *C. grandis* roots. In addition, SAMS activity was reduced in B-toxic *C. grandis* roots (Figure 7A). Based on these results, we concluded that BCAA biosynthesis might be disturbed in these roots.

We observed that the abundances of glycine-rich RNA-binding protein (GR-RBP) in *C. sinensis* (S21) and *C. grandis* (G21) roots and GR-RBP4 in *C. sinensis* roots (S13) increased when exposed to B-toxicity (Tables 2, 3), which agrees with the reports that the transcript levels of the genes encoding GR-RBPs were increased in higher plants following exposure to various abiotic stresses (Sachetto-Martins et al., 2000; Kwak et al., 2005). DEAD box RNA helicases, which may actively disrupt misfolded RNA structures by utilizing energy produced from ATP hydrolysis so that correct folding can occur, play key roles in plant response to various stresses (Li et al., 2008; Zhu et al., 2015). Thus, the down-accumulation of DEAD-box RNA helicase-like protein (G35) in B-toxic *C. grandis* roots might decrease *C. grandis* stress-tolerance, hence impairing its B-tolerance. However, the abundance of spliceosome RNA helicase BAT1 (G36) was increased in B-toxic *C. grandis* roots (Table 3).

### Proteins related to cellular transport

The abundances of four protein spots involved in cellular transport were increased in B-toxic *C. sinensis* (S20) and *C. grandis* (G18, 20, and 54) roots (Tables 2, 3), as reported on B-toxic leaves of barley (Atik et al., 2011), *C. sinensis* and *C. grandis* (Sang et al., 2015). However, the mRNA levels of all 13 B-toxicity-responsive genes involved in cellular transport were downregulated in *C. grandis* and *C. sinensis* roots except for one upregulated *H*^+^*-ATPase 4* (Guo et al., 2016). The difference between protein abundances and gene expression levels implies that post-translational modifications (PTMs) might affect protein levels. Atik et al. (2011) observed that heterologous expression of a gene encoding V-ATPase subunit E, a protein induced by B-toxicity in barley leaves, conferred yeast B-tolerance. The up-accumulation of V-ATPase B subunit (S20, and G18 and 20) in the two citrus species might be an adaptive response to B-toxicity by providing energy for compartmentation of excess B in vacuoles (Alemzadeh et al., 2006; Wang et al., 2011). Klychnikov et al. (2007) showed that plant V-ATPase could interact with 14-3-3 proteins. The up-accumulation of V-ATPase B subunit in B-toxic *C. sinensis* roots agrees with our data that the abundance of 14-3-3 family protein (S14) was increased in these roots. However, the abundance of 14-3-3-like protein GF14 phi (G9 and 10) was decreased in B-toxic *C. grandis* roots, implying that other factors was involved in the regulation of V-ATPase B subunit.

### Proteins related to biological regulation and signal transduction

B-toxicity increased the abundance of nucleoside diphosphate kinase 1 (NDPK1; S44) and 14-3-3 family protein (S14) in *C. sinensis* roots (Table 2), as observed on B-toxic *C. sinensis* leaves (Sang et al., 2015). However, the abundances of 14-3-3-like protein GF14 phi (G9 and 10) were reduced in B-toxic *C. grandis* roots (Table 3). Fukamatsu et al. (2003) demonstrated that *Arabidopsis* NDPK1 played a role in ROS response by interacting with three CATs. Overexpression of *NDPKs* conferred enhanced tolerance to multiple abiotic stresses in potato, alfalfa and poplar (Fukamatsu et al., 2003; Tang et al., 2008; Wang et al., 2014). 14-3-3 proteins, the master regulators of many signal transduction cascades, have a key role in stress-tolerance in higher plants (Chen et al., 2006). Transgenic potato plants overexpressing 14-3-3 protein genes displayed delayed leaf senescence and enhanced antioxidant activity, while transgenic potato plants with antisense 14-3-3 protein genes displayed early leaf senescence (Wilczynski et al., 1998; Lukaszewicz et al., 2002). Thus, the B-toxicity-induced up-accumulation of NDPK1 and 14-3-3 might contribute to the higher B-tolerance of *C. sinensis*.

### Comparison of B-toxicity-responsive proteins between roots and leaves

More B-toxicity-responsive proteins were identified in *C. grandis* (41) roots than in *C. sinensis* (31) roots (Tables 2, 3), while Sang et al. (2015) identified 45 and 55 DAPs from B-toxic *C. grandis* and *C. sinensis* leaves, respectively. As shown in Tables 2, 3 and Figures 4A,B, we identified more up-accumulated proteins than down-accumulated proteins in B-toxic *C. sinensis* and *C. grandis* roots, especially in B-toxic *C. sinensis* roots, but the reverse was the case in B-toxic *C. grandis* leaves although the number of up-accumulated proteins (27) in B-toxic *C. sinensis* leaves was slightly higher than that of down-accumulated proteins (23) (Sang et al., 2015). Furthermore, the vast majority of B-toxicity-responsive proteins were identified only in *C. sinensis* and *C. grandis* roots or leaves, only three proteins with the same accession No. were shared by *C. grandis* roots and leaves (Table 4). In addition, many other differences existed in B-toxicity-responsive proteins between roots and leaves of the two citrus species. For examples, the carbohydrate and energy metabolism-related proteins was the largest category of the B-toxicity-responsive proteins in *C. sinensis* and *C. grandis* leaves (Sang et al., 2015). Similar result has been obtained on NaCl-stressed *Citrus aurantium* leaves (Tanou et al., 2009). However, the protein and amino acid metabolism-related proteins was the most abundant category of B-toxicity-responsive proteins in *C. sinensis* and *C. grandis* roots (Tables 2, 3). In the previous study, we isolated similar up- (11) and down-accumulated (9) carbohydrate and energy metabolism-related proteins in B-toxic *C. sinensis* leaves, and more down- (16) than up-accumulated (9) proteins in B-toxic *C. grandis* leaves (Sang et al., 2015). However, more up- than down-accumulated proteins involved in carbohydrate and energy metabolism were identified in B-toxic *C. sinensis* (two up- and one down-accumulated) and *C. grandis* (four up- and two down-accumulated) roots (Tables 2, 3). As shown in Table 3, all four DAPs related to glycolysis (G22, 42, and 51) and tricarboxylic acid (TCA) cycle (G50) were up-accumulated, and ATP synthase subunit α (G46) involved in ATP was decreased in B-toxic *C. grandis* roots. By contrast, we isolated four down- and three up-accumulated proteins in glycolysis and TCA cycle and one up-accumulated mitochondrial ATP synthase in B-toxic *C. grandis* leaves (Sang et al., 2015). In B-toxic *C. sinensis* leaves, we obtained three up-accumulated malate dehydrogenases (MDHs), while only one down-accumulated MDH (S47) was detected in B-toxic *C. sinensis* roots (Table 2). Thus, the adaptive responses of carbohydrate and energy metabolism-related proteins to B-toxicity differed between roots and leaves of the two citrus species.

**Table 4:** **B-toxicity-responsive proteins shared by roots and leaves**.

**Protein identity**	**Accession No**.	**Fold change**			
		***C. grandis***		***C. sinensis***	
		**Roots**	**Leaves**	**Roots**	**Leaves**
Proteasome subunit α type, putative	gi|255584432	1.77	1.98	1.76	
V-ATPase B subunit	gi|4519264	1.80 (G18) 1.80 (G20)	1.67	2.85	
Triosphosphate isomerase-like protein	gi|76573375	2.02	0.14		
Cu/Zn-SOD	gi|2274917	1.91			2.99
S-adenosylmethionine synthetase 4	gi|222861722	0.43 (G33) 0.44 (G43)			1.63

*Data from Tables 2, 3 and Sang et al. (2015)*.

B-toxicity increased the abundances of proteins involved in protein degradation, and decreased the abundances of proteins related to protein biosynthesis in *C. grandis* and *C. sinensis* leaves (Sang et al., 2015). By contrast, the abundances of the two kinds of proteins were enhanced in B-toxic *C. grandis* and *C. sinensis* roots (Tables 2, 3). Interestingly, total soluble protein level was reduced only in B-toxic *C. grandis* roots and leaves (Sang et al., 2015; Guo et al., 2016). Thus, the causes for the decrease of total soluble proteins in B-toxic *C. grandis* roots and leaves were different.

We isolated seven up-accumulated, and one down- and three up-accumulated proteins involved in antioxidation and detoxification from B-toxic *C. grandis* and *C. sinensis* leaves, respectively (Sang et al., 2015), and two up-accumulated (G32 and 26) and one up-accumulated (S17) from B-toxic *C. grandis* and *C. sinensis* roots, respectively (Tables 2, 3). However, MDA concentration was increased only in B-toxic *C. grandis* leaves (Figures 1G,H). This might be related to the findings that B mainly accumulated in B-toxic *C. grandis* and *C. sinensis* leaves (Figures 1G,H; Jiang et al., 2009; Guo et al., 2014), and that the increased requirement for the detoxification of ROS and other toxic compounds such as reactive aldehtdes was greater in B-toxic *C. grandis* leaves than in B-toxic *C. sinensis* leaves (Sang et al., 2015).

To conclude, B-toxicity-induced alterations of protein profiles greatly differed between roots and leaves of the two citrus species.

## Conclusions

**Figure 8:**
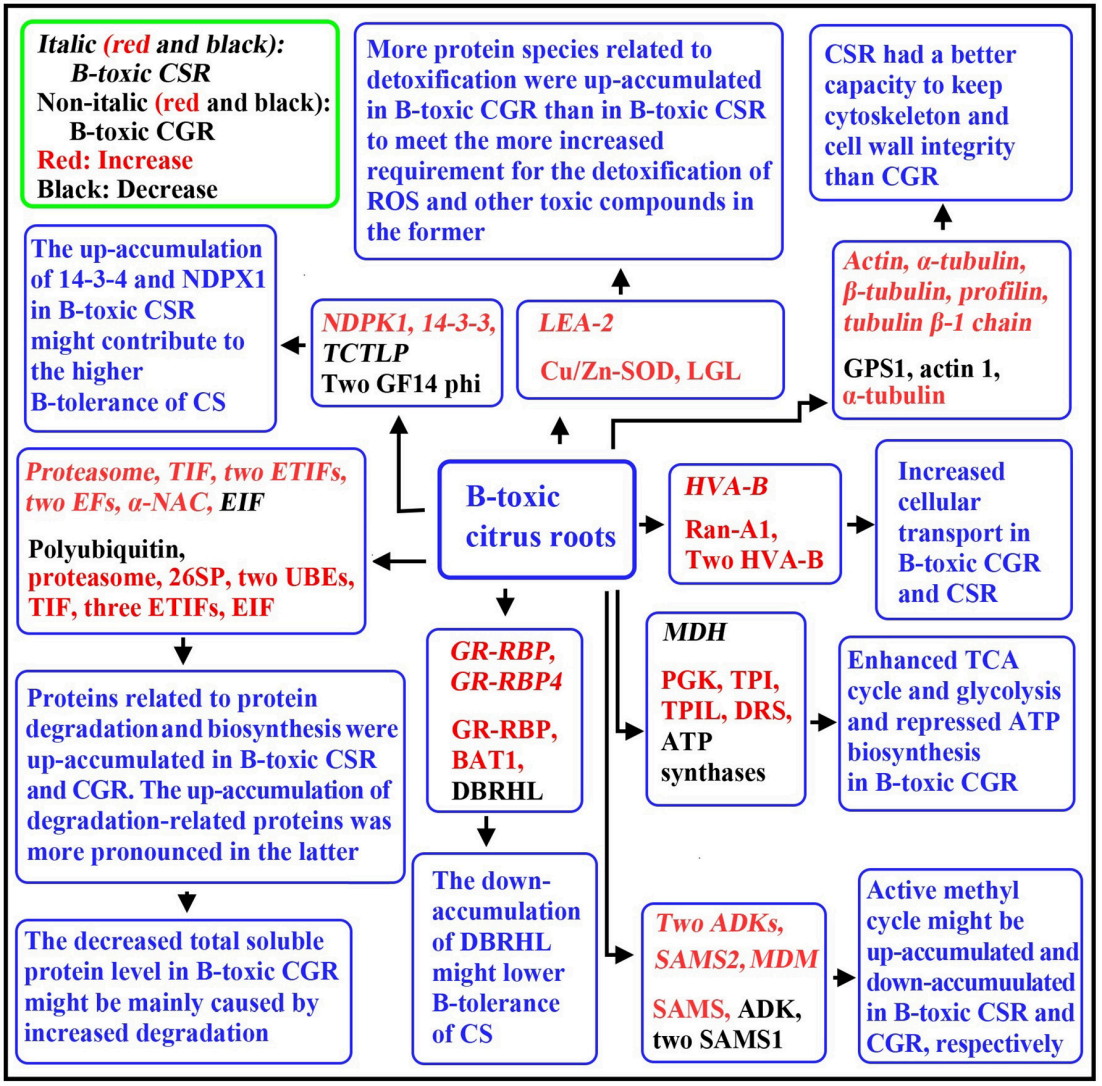
**A diagram for the responses of *C. grandis* and *C. sinensis* roots to B-toxicity**. CG, *C. grandis*; CGR, *C. grandis* roots; CS, *C. sinensis*; CSR, *C. sinensis* roots; DBRHL, DEAD-box RNA helicase-like protein; DRS, dihydrolipoyllysine-residue succinyltransferase component of 2-oxoglutarate dehydrogenase complex; EF, elongation factor; EIF, eukaryotic initiation factor; ETIF, eukaryotic translation initiation factor; GPS1, α-1,4-glucan-protein synthase 1; MDM, 5-methyltetrahy dropteroyltriglutamate-homocysteine methyltransferase; PGK, Phosphoglycerate kinase; 26SP, 26S proteasome subunit RPN12; TIF, translation initiation factor; TPI, triosephosphate isomerase; TPIL, triosphosphate isomerase-like protein; UBE, ubiquitin-conjugating enzyme; VHA-B, Vacuolar H^+^-ATPase B subunit.

Using a 2-DE based MS approach, we comparatively investigated the effects of B-toxicity on DAPs in roots of two citrus species with different B-tolerance and obtained 27 up- and four down-accumulated, and 28 up- and 13 down-accumulated proteins from B-toxic *C. sinensis* and *C. grandis*) roots, respectively. Most of B-toxicity-responsive proteins only were isolated from *C. sinensis* or *C. grandis* roots, only nine proteins were shared by the both. Great differences existed in B-toxicity-induced alterations of protein profiles between *C. sinensis* or *C. grandis* roots. Based on our findings, a diagram for the responses of *C. grandis* and *C. sinensis* roots to B-toxicity was presented in Figure 8. The higher B-tolerance of *C. sinensis* might be associated with (*a*) the B-toxicity-induced upregulation of the active methyl cycle and (*b*) the better performance in maintaining cell wall and cytoskeleton integrity. In addition, proteins related to nucleic acid metabolism, biological regulation and signal transduction might play a role in the higher B-tolerance of *C. sinensis*. To conclude, our findings provided some novel cues on the molecular mechanisms of citrus B-toxicity and B-tolerance.

## Data access

The mass spectrometry proteomics data have been deposited to the ProteomeXchange Consortium *via* the PRIDE partner repository with the dataset identifier PXD004050.

## Author contributions

WS and ZH contributed equally to this works. WS carried most of the experiment and analyzed the data; ZH drafted the manuscript; LY participated in the direction of this study; PG performed the qRT-PCR analysis; XY participated in the analysis of B; LC designed and directed the study and revised the manuscript. All authors have read and approved the final manuscript.

## Funding

This work was financially supported by the National Natural Science Grant of China (No. 31301740), the earmarked fund for China Agriculture Research System (No. CARS27) and the Provincial Natural Science Grant of Fujian, China (No. 2014J05033).

### Conflict of interest statement

The authors declare that the research was conducted in the absence of any commercial or financial relationships that could be construed as a potential conflict of interest.
