# Supplementary material for: Effects of High Toxic Boron Concentration on Protein Profiles in Roots of Two Citrus Species Differing in Boron-Tolerance Revealed by a 2-DE Based MS Approach
Source: Front Plant Sci. 2017 Feb 17;8:180. doi: 10.3389/fpls.2017.00180 (PMC5313502; doi:10.3389/fpls.2017.00180)
Supplement: Supplementary file 1 [file DataSheet1.DOC]

**Supplementary Materials**

**Table S1 | Differentially abundant proteins and their identification by MALDI-TOF/TOF-MS in B-toxic *Citrus sinensis*** roots

| **Spot No.** | **Protein identity** | **Accession No.** | **Mr(kDa)/pI Theor.** | **Mr (kDa)/**  **pI Exp.** | **Reference species** | **Protein score** | **Peptide score** | **High scoring peptide sequence** | **NMP** | **Ratio** | **Covered** sequence  (%) | **Charge** |
| --- | --- | --- | --- | --- | --- | --- | --- | --- | --- | --- | --- | --- |
| **Stress response** | | |  |  |  |  |  |  |  |  |  |  |
| **S16** | **Heat shock protein 83** | **gi|169296** | **80.77/4.95** | **81.1/5.0** | ***Ipomoea nil*** | **102** | **91** | **K.HNDDEQYIWESQAGGSFTVTR.D** | **8** | **0.42** | **14** | **1** |
| S32 | Mitochondrial chaperonin hsp60 | gi|20466256 | 61.242/5.66 | 60.3/5.18 | *Arabidopsis thaliana* | 288 | 148 | R.MISTSEEIAQVGTISANGER.E | 22 | 1.92 | 29 | 1 |
| S17 | Late-embryogenesis abundant protein 2 | gi|212552206 | 34.343/4.72 | 35.2/4.53 | *Glycine max* | 260 | 124 | K.LVSGLIPDSGTIHAHGEETVK.I | 14 | 1.87 | 36 | 1 |
| **Cell wall and cytoskeleton** | | |  |  |  |  |  |  |  |  |  |  |
| S30 | Actin | gi|6103623 | 41.564/5.30 | 42.6/5.81 | *Picea rubens* | 637 | 164 | K.DLYGNIVLSGGSTMFPGIADR.M | 25 | 1.63 | 50 | 1 |
| S29 | Alpha-tubulin | gi|321437427 | 46.368/4.90 | 46.2/4.8 | *Musa acuminata AAA Group* | 647 | 121 | R.LVSQVISSLTASLR.F | 21 | 1.70 | 47 | 1 |
| S18 | Beta-tubulin 14 | gi|166343835 | 49.98/4.76 | 50.4/4.6 | *Gossypium hirsutum* | 618 | 154 | K.GHYTEGAELIDSVLDVVR.K | 31 | 2.97 | 40 | 1 |
| S19 | Tubulin β-1 chain | gi|332197637 | 50.185/4.68 | 51.6/4.91 | *Arabidopsis thaliana* | 660 | 161 | K.GHYTEGAELIDAVLDVVR.K | 27 | 2.19 | 33 | 1 |
| S3 | Profilin | gi|12659206 | 14.133/4.90 | 13.8/4.94 | *Corylus avellana* | 89 | 71 | K.YMVIQGEPGAVIR.R | 9 | 1.57 | 43 | 1 |
| **Carbohydrate and energy metabolism** | | |  |  |  |  |  |  |  |  |  |  |
| S27 | Adenosine kinase 2, partial | gi|149391003 | 26.477/4.88 | 25.9/5.63 | *Oryza sativa Indica Group* | 80 | 66 | K.VLPFVDYIFGNETEAR.I | 7 | 1.82 | 21 | 1 |
| **S28** | **Adenosine kinase isoform 1T-like protein** | **gi|82400168** | **37.549/5.01** | **38.6/5.71** | ***Solanum tuberosum*** | **113** | **96** | **K.ALPYMDFVFGNETEAR.I + Oxidation (M)** | **6** | **1.81** | **20** | **1** |
| S47 | Malate dehydrogenase , partial | gi|160690776 | 14.017/7.03 | 15.4/5.05 | *Citrus trifoliata* | 220 | 105 | K.KPWAHEHEPVNNLLDAVK.V | 6 | 0.50 | 38 | 1 |
| **Protein and amino acid metabolism** | | |  |  |  |  |  |  |  |  |  |  |
| **S9** | **Proteasome subunit alpha type, putative** | **gi|255584432** | **27.0/4.73** | **27.8/4.75** | ***Ricinus communis*** | **191** | **83** | **R.LFQVEYAIEAIK.L** | **11** | **1.76** | **42** | **1** |
| S12 | Elongation factor 1-delta 1 | gi|226505926 | 24.767/4.39 | 23.8/4.38 | *Zea mays* | 219 | 70 | K.KLDEYLLTR.S | 11 | 2.41 | 20 | 1 |
| S15 | Elongation factor 2 | gi|195646972 | 93.862/6.00 | 94.2/6.51 | *Zea mays* | 143 | 133 | K.STLTDSLVAAAGIIAQEVAGDVR.M | 8 | 2.90 | 9 | 1 |
| S23 | Translation initiation factor | gi|197312901 | 16.419/4.98 | 16.2/5.12 | *Rheum australe* | 188 | 91 | R.ELVFKEDGQEYAQVLR.M | 7 | 1.72 | 26 | 1 |
| S40 | Eukaryotic translation initiation factor 2 beta subunit-like | gi|82621136 | 29.831/6.08 | 30.6/6.54 | *Solanum tuberosum* | 101 | 78 | R.DYEYEELLGR.V | 9 | 1.85 | 32 | 1 |
| **S36** | **Eukaryotic translation initiation factor 5A1** | **gi|217038830** | **17.387/5.60** | **17.2/5.7** | ***Glycine max*** | **153** | **60** | **K.DDLRLPTDENLLSQIK.D** | **10** | **3.13** | **28** | **1** |
| **S34** | **Eukaryotic initiation factor 5A (2)** | **gi|19702** | **17.353/5.60** | **18.5/6.4** | ***Nicotiana plumbaginifolia*** | **120** | **54** | **K.TYPQQAGTIR.K** | **10** | **0.48** | **58** | **1** |
| S8 | Alpha chain of nascent polypeptide associated complex | gi|124484511 | 21.911/4.32 | 22.1/4.52 | *Nicotiana benthamiana* | 242 | 137 | K.IEDLSSQLQTQAAEQFK.A | 8 | 2.03 | 20 | 1 |
| S43 | Alanine aminotransferase 2 | gi|332197185 | 47.68/6.32 | 48.2/6.61 | *Arabidopsis thaliana* | 138 | 131 | R.ALVVINPGNPTGQVLAEENQR.D | 5 | 2.28 | 11 | 1 |
| S41 | S-adenosylmethionine synthase 2 | gi|1655578 | 42.977/5.51 | 43.3/5.93 | *Catharanthus roseus* | 241 | 113 | R.FVIGGPHGDAGLTGR.K | 19 | 1.89 | 47 | 1 |
| S39 | 5-methyltetrahy dropteroyltriglutamate-homocysteine methyltransferase, putative | gi|255569484 | 84.668/6.09 | 85.6/6.52 | *Ricinus communis* | 373 | 113 | K.LQEELDIDVLVHGEPER.N | 19 | 1.97 | 27 | 1 |
| S42 | Transaminase mtnE, putative | gi|255562088 | 50.396/6.95 | 51.8/6.05 | *Ricinus communis* | 244 | 136 | K.THVVTTPGSGFGPGGEGFIR.V | 10 | 1.74 | 22 | 1 |
| **S31** | **Ketol-acid reductoisomerase** | **gi|295291644** | **63.583/6.49** | **64.6/6.62** | ***Catharanthus roseus*** | **286** | **159** | **K.EINGAGINSSFAVHQDVDGR.A** | **16** | **2.07** | **21** | **1** |
| **Nucleic acid metabolism** | | |  |  |  |  |  |  |  |  |  |  |
| S13 | Glycine-rich RNA-binding protein 4 | gi|332643299 | 14.12/5.03 | 15.6/8.68 | *Arabidopsis thaliana* | 133 | 118 | K.LFVGGLSWGTDDSSLK.Q | 4 | 1.85 | 33 | 1 |
| **S21** | **Glycine-rich RNA-binding protein** | **gi|7024451** | **16.839/4.98** | **17.4/7.85** | ***Citrus unshiu*** | **227** | **57** | **R.GFGFVTFR.D** | **10** | **1.97** | **28** | **1** |
| **Cellular transport** | |  |  |  |  |  |  |  |  |  |  |  |
| **S20** | **Vacuolar H^+^-ATPase B subunit** | **gi|4519264** | **54.329/4.91** | **55.6/4.91** | ***Citrus unshiu*** | **641** | **93** | **R.VTLFLNLANDPTIER.I** | **35** | **1.65** | **53** | **1** |
| **Biological regulation and signal transduction** | | | |  |  |  |  |  |  |  |  |  |
| S44 | Nucleoside diphosphate kinase 1 | gi|255571035 | 176.41/5.09 | 179.1/6.3 | *Ricinus communis* | 136 | 127 | R.TIIGATNPAQSAPGTIR.G | 4 | 1.51 | 20 | 1 |
| S14 | 14-3-3 family protein | gi|291162645 | 29.404/4.74 | 30.5/4.71 | *Dimocarpus longan* | 241 | 128 | R.LGLALNFSVFYYEILNSPDR.A | 7 | 1.52 | 33 | 1 |
| S6 | Translationally controlled tumor-like protein | gi|115187479 | 19.116/4.54 | 20.0/4.7 | *Arachis hypogaea* | 286 | 69 | K.VVDIVDTFR.L | 9 | 0.44 | 31 | 1 |
| **Others** | | |  |  |  |  |  |  |  |  |  |  |
| **S37** | **Unnamed protein product, partial** | **gi|296088008** | **17.163/5.21** | **18.2/7.93** | ***Vitis vinifera*** | **709** | **172** | **K.LQDILPGIINQLGPDNLDNLR.K** | **17** | **1.84** | **51** | **1** |
| **Unidentified protein spots** | | | |  |  |  |  |  |  |  |  |  |
| S25 | Predicted protein | gi|168013983 | 27.2/5.53 | 26.8/5.62 | *Physcomitrella patens subsp. patens* | 80 |  |  | 3 | 1.84 | 11 |  |
| S 48 | Indole-3-acetic acid inducible 9 | gi|304308539 | 32.573/5.17 | 33.4/5.58 | *Arabidopsis thaliana* | 35 |  |  | 9 | 4.886 |  |  |
| S 7 | Late embryogenesis abundant  domain-containing protein | gi|332642191 | 24.172/5.13 | 25.3/4.73 | *Arabidopsis thaliana* | 36 |  |  | 8 | 2.169 |  |  |
| S 11 | AFB1 | gi|170677210 | 17.737/5.09 | 17.2/6.0 | *Arabidopsis thaliana* | 34 |  |  | 10 | 1.579 |  |  |
| S 46 | Nbs-lrr resistance protein | gi|222862576 | 11.6251/5.65 | 12.8/6.22 | *Populus trichocarpa* | 28 |  |  | 7 | 1.794 |  |  |
| S 1 | Alpha subunit of F-actin capping protein | gi|297829078 | 35.087/4.61 | 33.7/4.2 | *Arabidopsis lyrata subsp. lyrata* | 47 |  |  | 9 | 1.65 |  |  |
| S 4 | Actin depolymerizing factor 2 | gi|332644578 | 15.735/5.24 | 16.3/5.91 | *Arabidopsis thaliana* | 41 |  |  | 9 | 2.133 |  |  |
| S 22 | ATP synthase d chain | gi|307136395 | 19.818/5.12 | 18.6/5.73 | *Cucumis melo subsp. melo* | 49 |  |  | 4 | 0.549 |  |  |
| S 5 | UDP-galactose 4-epimerase-like protein | gi|27529690 | 44.799/5.11 | 45.3/5.91 | *Oryza sativa Japonica Group* | 58 |  |  | 15 | 1.502 |  |  |
| S 26 | Calcium-dependent protein kinase 34 | gi|297812119 | 58.235/5.76 | 58.1/6.02 | *Arabidopsis lyrata subsp. lyrata* | 54 |  |  | 15 | 2.221 |  |  |
| S 2 | B3 DNA binding domain containing protein | gi|31433278 | 33.105/5.02 | 33.5/5.78 | *Oryza sativa Japonica Group* | 48 |  |  | 11 | 1.917 |  |  |
| S 10 | Homeobox-leucine zipper protein ATHB-7 | gi|330255646 | 30.250/5.52 | 28.9/5.37 | *Arabidopsis thaliana* | 27 |  |  | 6 | 1.632 |  |  |
| S 24 | Glycine-rich RNA-binding protein RGP-1c | gi|45533923 | 13.235/5.14 | 14.8/6.12 | *Nicotiana sylvestris* | 32 |  |  | 5 | 1.862 |  |  |
| S 35 | Glycine rich protein 17 | gi|332003788 | 53.159/5.36 | 55.6/5.92 | *Arabidopsis thaliana* | 37 |  |  | 11 | 1.566 |  |  |
| S33 | Hypothetical protein OsJ_03809 | gi|125572367 | 10.784/9.2 | 79.66/5.79 | *Oryza sativa Japonica Group* | 42 |  |  | 5 | 1.901 |  |  |
| S38 | Elongation factor Tu | gi|291586303 | 26.112/5.06 | 40.15/6.25 | *Flabellia petiolata* | 64 |  |  | 12 | 2.634 |  |  |
| S45 | Predicted protein | gi|326530894 | 21.718/4.85 | 72.08/6.52 | *Hordeum vulgare subsp. vulgare* | 40 |  |  | 3 | 1.814 |  |  |

*Spot number corresponds to the 2-DE gel imagines in* ***Figure 2A-B.*** *NMP means the number of peptides. Ratio means the ratio of B-toxic roots to controls. Covered sequence (%) means the ratio of the number of amino acids of the matched peptides to the number of amino acids of the full-length protein. Proteins shared by* C. sinensis *and* C. grandis *roots were marked in bold.*

**Table S2 | Differentially abundant proteins and their identification by MALDI-TOF/TOF-MS in B-toxic *Citrus grandis*** roots

| **Spot No.** | **Protein identity** | **Accession No.** | **Mr(kDa)/pI Theor.** | **Mr (kDa)/pI Exp.** | **Reference species** | **Protein score** | **Peptide score** | **High scoring peptide sequence** | **NMP** | **Ratio** | **Covered sequence**  **(%)** | **Charge** |
| --- | --- | --- | --- | --- | --- | --- | --- | --- | --- | --- | --- | --- |
| **Stress response** | | |  |  |  |  |  |  |  |  |  |  |
| G32 | Cu/Zn superoxide dismutase, partial | gi|2274917 | 12.784/5.82 | 13.5/5.61 | *Citrus sinensis* | 179 | 85 | K.TIPLSGTNSVIGR.G | 7 | 1.91 | 52 | 1 |
| G26 | Lactoylglutathione lyase, putative | gi|255554865 | 31.547/5.11 | 31.8/7.63 | *Ricinus communis* | 219 | 79 | K.DPDGYIFELIQR.G | 12 | 1.85 | 40 | 1 |
| **G16** | **Heat shock protein 83** | **gi|169296** | **80.820/4.95** | **81.4/5.12** | ***Ipomoea nil*** | **102** | **91** | **K.HNDDEQYIWESQAGGSFTVTR.D** | **8** | **1.60** | **14** | **1** |
| G19 | 60-kDa chaperonin-60 alpha -polypeptide precursor, partial | gi|289365 | 57.692/4.84 | 58.4/4.58 | *Brassica napus* | 435 | 118 | K.DSTTLIADAASKDELQAR.I | 26 | 1.92 | 41 | 1 |
|  |  |  |  |  |  |  |  |  |  |  |  |  |
| G12 | Chilling-responsive protein | gi|153793260 | 35.739/4.85 | 36.4/4.6 | *Nicotiana tabacum* | 284 | 110 | R.IKVDLIVDVPVFGR.L | 9 | 1.88 | 12 | 1 |
| **Cell wall and cytoskeleton** | | |  |  |  |  |  |  |  |  |  |  |
| G52 | Alpha-1,4-glucan-protein synthase 1 | gi|195623832 | 40.905/6.60 | 40.2/6.21 | *Zea mays* | 422 | 120 | R.ELIGPAMYFGLMGDGQPIGR.Y | 20 | 0.48 | 49 | 1 |
| G34 | Actin 1 | gi|255115691 | 41.665/5.31 | 41.9/5.14 | *Boehmeria nivea* | 169 | 98 | K.NYELPDGQIITIGAER.F | 12 | 040 | 30 | 1 |
| G25 | Alpha-tubulin | gi|334261583 | 49.446/4.99 | 50.3/5.0 | *Pellia endiviifolia* | 297 | 122 | R.AFVHWYVGEGMEEGEFSEAR.E | 12 | 2.02 | 26 | 1 |
| **Carbohydrate and energy metabolism** | | | |  |  |  |  |  |  |  |  |  |
| G46 | ATP synthase subunit α | gi|222356608 | 40.289/8.59 | 41.6/8.9 | *Afrothismia hydra* | 323 | 173 | R.EVAAFAQFGSDLDAATQALLNR.G | 13 | 0.47 | 29 | 1 |
| **G24** | **Adenosine kinase isoform 1T-like protein** | **gi|82400168** | **37.572/5.01** | **37.2/5.16** | ***Solanum tuberosum*** | **113** | **96** | **K.ALPYMDFVFGNETEAR.I + Oxidation (M)** | **6** | **0.49** | **20** | **1** |
| G22 | Triosephosphate isomerase | gi|295687231 | 33.119/6.66 | 33.6/5.94 | *Gossypium hirsutum* | 316 | 93 | K.QEDIDGFLVGGASLK.G | 12 | 2.17 | 25 | 1 |
| G42 | Triosphosphate isomerase-like protein | gi|76573375 | 27.711/5.88 | 28.3/5.9 | *Solanum tuberosum* | 215 | 99 | K.VATPAQAQEVHFELR.K | 8 | 2.02 | 23 | 1 |
| G51 | Phosphoglycerate kinase | gi|332198142 | 42.131/5.49 | 43.2/5.61 | *Arabidopsis thaliana* | 120 | 53 | K.FLKPSVAGFLMQK.E + Oxidation (M) | 9 | 1.74 | 15 | 1 |
| G50 | Dihydrolipoyllysine-residue succinyltransferase component of 2-oxoglutarate dehydrogenase complex | gi|226509380 | 48.749/5.11 | 49.4/5.64 | *Zea mays* | 92 | 60 | K.AAVSALQNQPIVNAVIDGDDIIYR.D | 12 | 1.56 | 51 | 1 |
| **Protein and amino acid metabolism** | | |  |  |  |  |  |  |  |  |  |  |
| **G6** | **Proteasome subunit alpha type, putative** | **gi|255584432** | **27.017/4.73** | **27.8/4.75** | ***Ricinus communis*** | **191** | **83** | **R.LFQVEYAIEAIK.L** | **11** | **1.77** | **42** | **1** |
| G13 | 26S proteasome subunit RPN12 | gi|32700048 | 30.701/4.81 | 31.5/4.66 | *Arabidopsis thaliana* | 218 | 102 | K.HAVELEQSFMEGAYNR.V | 9 | 1.91 | 29 | 1 |
| G48 | Ubiquitin-conjugating enzyme variant | gi|257196367 | 16.630/6.20 | 17.6/6.6 | *Citrus sinensis* | 438 | 76 | M.TLGSGGSSVVVPR.N | 22 | 1.63 | 80 | 1 |
| G53 | Ubiquitin-conjugating enzyme E2 35 | gi|332198044 | 17.191/6.74 | 18.3/6.41 | *Arabidopsis thaliana* | 387 | 115 | R.LLSEPAPGISASPSEDNMR.Y | 14 | 2.97 | 47 | 1 |
| G29 | Polyubiquitin, partial | gi|284927592 | 11.992/8.2 | 12.3/5.12 | *Citrus sinensis* | 389 | 95 | K.IQDKEGIPPDQQR.L | 11 | 0.31 | 66 | 1 |
| G7 | Translation initiation factor IF6 | gi|332645889 | 26.482/4.63 | 27.8/4.52 | *Arabidopsis thaliana* | 246 | 143 | K.ETEEIIADVLGVEVFR.Q | 6 | 1.74 | 16 | 1 |
| G40 | Eukaryotic translation initiation factor 5A isoform VII | gi|33325129 | 17.471/5.60 | 18.2/5.9 | *Hevea brasiliensis* | 121 | 55 | K.TYPQQAGTIR.K | 10 | 4.75 | 40 | 1 |
| **G11** | **Eukaryotic translation initiation factor 5A1** | **gi|217038830** | **17.397/5.60** | **17.9/5.58** | ***Glycine max*** | **239** | **73** | **R.LPTDENLLSQIK.D** | **13** | **2.27** | **32** | **1** |
| **G41** | **Eukaryotic translation initiation factor 5A1** | **gi|217038830** | **17.397/5.60** | **18.4/5.6** | ***Glycine max*** | **236** | **82** | **K.DDLRLPTDENLLSQIK.D** | **10** | **1.55** | **65** | **1** |
| **G30** | **Eukaryotic initiation factor 5A (2)** | **gi|19702** | **17.363/5.60** | **18.7/5.52** | ***Nicotiana plumbaginifolia*** | **120** | **54** | **K.TYPQQAGTIR.K** | **10** | **0.36** | **58** | **1** |
| G33 | S-adenosylmethionine synthetase 1 family protein | gi|222861722 | 43.213/5.68 | 43.5/5.82 | *Populus trichocarpa* | 598 | 141 | K.VLVNIEQQSPDIAQGVHGHFTK.R | 18 | 0.43 | 39 | 1 |
| G43 | S-adenosylmethionine synthetase 1 family protein | gi|222861722 | 43.213/5.68 | 44.1/5.73 | *Populus trichocarpa* | 677 | 152 | R.VHTVLISTQHDETVTNDEIAADLK.E | 17 | 0.44 | 45 | 1 |
| G44 | S-adenosylmethionine synthetase | gi|14600072 | 43.184/5.67 | 43.8/5.52 | *Brassica juncea* | 490 | 131 | K.VLVNIEQQSPDIAQGVHGHFTK.R | 23 | 2.01 | 33 | 1 |
| **G27** | **Ketol-acid reductoisomerase** | **gi|295291644** | **63.623/6.49** | **64.2/6.81** | ***Catharanthus roseus*** | **286** | **159** | **K.EINGAGINSSFAVHQDVDGR.A** | **16** | **1.68** | **21** | **1** |
| **G28** | **Ketol-acid reductoisomerase** | **gi|295291644** | **63.623/6.49** | **65.1/6.9** | ***Catharanthus roseus*** | **332** | **159** | **K.EINGAGINSSFAVHQDVDGR.A** | **16** | **1.81** | **16** | **1** |
| **Nucleic acid metabolism** | | |  |  |  |  |  |  |  |  |  |  |
| **G21** | **Glycine-rich RNA-binding protein** | **gi|7024451** | **16.848/7.85** | **17.3/4.98** | ***Citrus unshiu*** | **227** | **57** | **R.GFGFVTFR.D** | **10** | **2.64** | **28** | **1** |
| G35 | DEAD-box RNA helicase-like protein | gi|283049402 | 46.935/5.48 | 47.2/5.6 | *Prunus persica* | 735 | 191 | R.GIDVQQVSLVINYDLPTQPENYLHR.I | 33 | 0.42 | 50 | 1 |
| G36 | Spliceosome RNA helicase BAT1 | gi|226528292 | 45.146/6.03 | 48.1/6.52 | *Zea mays* | 434 | 150 | R.VNIVINYDMPDSADTYLHR.V | 24 | 1.97 | 41 | 1 |
| **Cellular transport** | |  |  |  |  |  |  |  |  |  |  |  |
| **G18** | **Vacuolar H^+^-ATPase B subunit** | **gi|4519264** | **54.362/4.91** | **55.2/5.02** | ***Citrus unshiu*** | **641** | **93** | **R.VTLFLNLANDPTIER.I** | **35** | **1.80** | **53** | **1** |
| **G20** | **Vacuolar H^+^-ATPase B subunit** | **gi|4519264** | **54.362/4.91** | **54.9/5.1** | ***Citrus unshiu*** | **121** | **80** | **R.QIYPPINVLPSLSR.L** | **13** | **1.80** | **15** | **1** |
| G54 | GTP-binding nuclear protein Ran-A1 | gi|192913008 | 24.9/6.38 | 25.8/6.51 | *Elaeis guineensis* | 86 | 57 | K.SNYNFEKPFLYLAR.K | 8 | 2.24 | 28 | 1 |
| **Biological regulation and signal transduction** | | | | |  |  |  |  |  |  |  |  |
| G9 | 14-3-3-like protein GF14 phi | gi|332193639 | 30.193/4.79 | 31.5/4.63 | *Arabidopsis thaliana* | 483 | 190 | R.LGLALNFSVFYYEILNSPDR.A | 22 | 0.48 | 53 | 1 |
| G10 | 14-3-3-like protein GF14 phi | gi|332193639 | 30.193/4.79 | 29.8/5.12 | *Arabidopsis thaliana* | 436 | 155 | R.LGLALNFSVFYYEILNSPDR.A | 21 | 0.41 | 54 | 1 |
| **Others** | | |  |  |  |  |  |  |  |  |  |  |
| G15 | 12-oxo-phytodienoic acid reductase2 | gi|162459589 | 41.665/6.08 | 42.4/6.31 | *Zea mays* | 110 | 64 | K.LFLANPDLPR.R | 11 | 0.45 | 31 | 1 |
| G49 | 12-oxo-phytodienoic acid reductase2 | gi|162459589 | 41.665/6.08 | 42.8/6.25 | *Zea mays* | 98 | 49 | K.LFLANPDLPR.R | 11 | 0.49 | 31 | 1 |
| **G2** | **Unnamed protein product, partial** | **gi|296088008** | **17.173/7.93** | **17.8/5.16** | ***Vitis vinifera*** | **503** | **158** | **K.LQDILPGIINQLGPDNLDNLR.K** | **13** | **1.69** | **35** | **1** |
| **G39** | **Unnamed protein product, partial** | **gi|296088008** | **17.173/7.93** | **17.2/5.21** | ***Vitis vinifera*** | **709** | **172** | **K.LQDILPGIINQLGPDNLDNLR.K** | **17** | **3.63** | **56** | **1** |
| **Unidentified protein spots (Score < 75)** | | | |  |  |  |  |  |  |  |  |  |
| G45 | Germin-like protein 6 | gi|332007011 | 14.128/5.09 | 14.3/5.61 | *Arabidopsis thaliana* | 106 |  |  | 2 | 1.55 | 12 |  |
| G37 | Glycine rich protein 2 | gi|332661564 | 19.153/5.62 | 20.3/5.46 | *Arabidopsis thaliana* | 124 |  |  | 2 | 3.36 | 10 |  |
| G23 | Sophorol reductase | gi|4336887 | 35.696/8.34 | 36.6/5.05 | *Pisum sativum* | 96 |  |  | 4 | 1.94 | 10 |  |
| G 5 | Lti65(low-temperature-induced 65 kD protein ) | gi|16388 | 64.510/5.14 | 66.2/5.87 | *Arabidopsis thaliana* | 46 |  |  | 1 | 2.611 |  |  |
| G 47 | Late embryogenesis abundant protein group 8 protein | gi|312599843 | 10.216/4.83 | 10.4/5.01 | *Arachis hypogaea* | 39 |  |  | 6 | 0.503 |  |  |
| G 55 | Protein kinase | gi|13561063 | 60.930/5.77 | 60.6/6.02 | *Medicago sativa* | 50 |  |  | 16 | 1.796 |  |  |
| G 14 | Serine-threonine protein kinase, plant-type, putative | gi|223535154 | 80.692/5.02 | 82.4/5.94 | *Ricinus communis* | 53 |  |  | 1 | 0.502 |  |  |
| G 8 | Homeobox-leucine zipper protein ATHB-7 | gi|330255646 | 30.269/5.52 | 28.9/5.88 | *Arabidopsis thaliana* | 27 |  |  | 6 | 1.625 |  |  |
| G 1 | Telomerase reverse transcriptase catalytic subunit | gi|60476126 | 77.262/5.07 | 76.4/6/01 | *Scilla peruvian a* | 43 |  |  | 15 | 0.5 |  |  |
| G 17 | Acyl-CoA binding protein 5, acyl-CoA-binding domain 5 | gi|297812953 | 71.752/5.89 | 73.5/6.0 | *Arabidopsis lyrata subsp. lyrata* | 47 |  |  | 13 | 0.302 |  |  |
| G 31 | F-box family protein | gi|297834198 | 45.725/5.09 | 44.7/5.83 | *Arabidopsis lyrata subsp. lyrata* | 39 |  |  | 9 | 0.476 |  |  |
| G 3 | Predicted protein | gi|162664923 | 45.905/6.25 | 34.46/5.02 | *Physcomitrella patens subsp. patens* | 50 |  |  | 1 | 0.446 |  |  |
| G 4 | Heat shock protein 90 (ISS) | gi|116055649 | 89.636/6.12 | 29.24/5.03 | *Ostreococcus tauri* | 56 |  |  | 2 | 1.642 |  |  |
| G 38 | Hypothetical protein ARALYDRAFT_899412 | gi|297835868 | 15.677/6.45 | 33.76/6.16 | *Arabidopsis lyrata subsp. lyrata* | 64 |  |  | 7 | 0.408 |  |  |

*Spot number corresponds to the 2-DE imagines in* ***Figure 2C-D.*** *NMP means the number of peptides.Ratio means the ratio of B-toxic roots to controls. Covered sequence (%) means the ratio of the number of amino acids of the matched peptides to the number of amino acids of the full-length protein. Proteins shared by* C. sinensis *and* C. grandis *roots were marked in bold.*

**Table S3 | Master list of proteins identified in MALDI TOF/TOF MS from B-toxic *Citrus sinensis* roots using** 2DE and DIGE experiments

| **S16 gi|169296 Mass: 80770 Score: 102 Expect: 0.00012 Queries matched: 8 heat shock protein 83 [Ipomoea nil]**  Observed Mr(expt) Mr(calc) Delta Start End Miss Ions Peptide  1323.6270 1322.6197 1322.5990 0.0207 185 - 194 0 --- K.EDQLEYLEER.R  1905.9847 1904.9774 1904.8971 0.0803 610 - 625 1 --- K.TMEINPDNGIMEELRK.R + Oxidation (M)  1922.0072 1920.9999 1920.8921 0.1079 610 - 625 1 --- K.TMEINPDNGIMEELRK.R + 2 Oxidation (M)  2440.1489 2439.1416 2439.0727 0.0689 146 - 166 0 91 K.HNDDEQYIWESQAGGSFTVTR.D  2440.1489 2439.1416 2439.0727 0.0689 146 - 166 0 --- K.HNDDEQYIWESQAGGSFTVTR.D  2716.2830 2715.2757 2715.2528 0.0229 569 - 592 1 --- R.IVDSPCCLVTGEYGWTANMERIMK.A  2748.3667 2747.3594 2747.2426 0.1168 569 - 592 1 --- R.IVDSPCCLVTGEYGWTANMERIMK.A + 2 Oxidation (M)  3353.7957 3352.7884 3352.4697 0.3187 668 - 698 1 --- R.MLKLGLSIDEEEAGDDADMPALEEEAGEESK.M + 2 Oxidation (M)  No match to: 842.5065, 952.4592, 1015.4484, 1137.5433, 1137.5433, 1141.5863, 1379.7140, 1448.7358, 1475.7784, 1595.8881, 1603.7698, 1619.7762, 1625.8671, 1651.7876, 1667.7836, 1667.7836, 1683.7861, 1683.7861, 1716.9049, 1728.8517, 1748.9934, 1809.9271, 1834.9901, 1845.9000, 1857.9751, 1857.9751, 1872.9727, 1873.9613, 1878.9856, 1886.9611, 1894.0288, 1895.9562, 1921.0573, 1928.9600, 1937.9818, 1943.9808, 1967.9675, 1967.9675, 1968.9586, 1983.9883, 1985.0415, 1985.9752, 1985.9752, 1994.0089, 2007.9799, 2013.0001, 2023.0018, 2025.9960, 2028.0140, 2056.0239, 2123.0325, 2170.1367, 2189.1533, 2197.1213, 2211.1782, 2225.1982, 2230.2561, 2239.2070, 2246.2615, 2278.1370, 2283.2244, 2292.1292, 2306.1699, 2306.1699, 2332.2051, 2342.1909, 2369.2695, 2395.2202, 2417.3176, 2444.1387, 2534.4390, 2563.2393, 2564.2622, 2568.2332, 2569.1985, 2578.2705, 2596.2229, 2596.2229, 2614.2961, 2637.2612, 2653.2832, 2655.2761, 2655.2761, 2660.2803, 2665.3281, 2669.2849, 2671.2727, 2671.2727, 2677.3132, 2685.2712, 2687.2668, 2687.2668, 2691.3149, 2695.3367, 2705.2546, 2712.2991, 2720.3137, 2751.3667, 2807.3826, 3146.6179, 3256.6123, 3274.5911, 3302.6169, 3346.7598 |
| --- |
| **S32 gi|20466256 Mass: 61242 Score: 288 Expect: 2.9e-023 Queries matched: 22 mitochondrial chaperonin hsp60 [Arabidopsis thaliana]**  Observed Mr(expt) Mr(calc) Delta Start End Miss Ions Peptide  847.3776 846.3703 846.3984 -0.0280 308 - 315 0 --- K.APGFGENR.K  975.4744 974.4672 974.4933 -0.0262 308 - 316 1 --- K.APGFGENRK.A  1196.7112 1195.7039 1195.7288 -0.0249 468 - 478 0 --- K.IGVQIIQNALK.T  1203.5634 1202.5561 1202.6255 -0.0694 424 - 434 1 --- K.DRVTDALNATK.A  1235.5631 1234.5558 1234.5798 -0.0239 136 - 147 0 --- K.SVAAGMNAMDLR.R  1251.5593 1250.5520 1250.5747 -0.0227 136 - 147 0 --- K.SVAAGMNAMDLR.R + Oxidation (M)  1267.5564 1266.5491 1266.5696 -0.0205 136 - 147 0 --- K.SVAAGMNAMDLR.R + 2 Oxidation (M)  1327.6808 1326.6735 1326.6931 -0.0196 67 - 78 0 74 R.NVVIEQSWGAPK.V  1327.6808 1326.6735 1326.6931 -0.0196 67 - 78 0 --- R.NVVIEQSWGAPK.V  1331.6827 1330.6754 1330.7166 -0.0411 187 - 198 1 --- R.EIGELIAKAMEK.V  1343.6747 1342.6674 1342.7456 -0.0781 199 - 211 1 --- K.VGKEGVITIQDGK.T  1407.6848 1406.6775 1406.6758 0.0017 136 - 148 1 --- K.SVAAGMNAMDLRR.G + Oxidation (M)  1524.7472 1523.7399 1523.7541 -0.0141 212 - 224 0 --- K.TLFNELEVVEGMK.L + Oxidation (M)  1540.7450 1539.7377 1539.8157 -0.0780 65 - 78 1 --- K.GRNVVIEQSWGAPK.V  1665.7860 1664.7787 1664.8807 -0.1020 243 - 256 1 --- K.CELDDPLILIHEKK.I  1908.9728 1907.9655 1907.9662 -0.0007 212 - 227 1 --- K.TLFNELEVVEGMKLDR.G + Oxidation (M)  2093.0059 2091.9986 2092.0106 -0.0120 167 - 186 0 148 R.MISTSEEIAQVGTISANGER.E  2093.0059 2091.9986 2092.0106 -0.0120 167 - 186 0 --- R.MISTSEEIAQVGTISANGER.E  2109.0154 2108.0081 2108.0055 0.0026 167 - 186 0 (136) R.MISTSEEIAQVGTISANGER.E + Oxidation (M)  2109.0154 2108.0081 2108.0055 0.0026 167 - 186 0 --- R.MISTSEEIAQVGTISANGER.E + Oxidation (M)  2211.1133 2210.1060 2210.2422 -0.1362 459 - 478 1 --- K.LPTANFDQKIGVQIIQNALK.T  2946.3965 2945.3892 2945.4875 -0.0983 98 - 127 1 --- K.NVGASLVKQVANATNDVAGDGTTCATVLTR.A  No match to: 705.3077, 823.4503, 842.4784, 855.0481, 871.0239, 972.5553, 972.5553, 1023.4828, 1036.5039, 1060.0583, 1187.5590, 1359.6680, 1430.7164, 1430.7164, 1452.6991, 1468.6769, 1544.7035, 1562.7372, 1577.7719, 1586.7146, 1594.7974, 1680.9543, 1695.8595, 1711.7833, 1795.9073, 1858.9523, 1860.9500, 1860.9500, 1873.9395, 1876.9500, 1885.0470, 1885.0470, 1890.0404, 1891.0059, 1907.0093, 1911.9729, 1922.9939, 1924.9738, 1964.9631, 2027.0017, 2027.9668, 2031.0034, 2043.9996, 2044.9908, 2044.9908, 2054.1074, 2060.9922, 2064.0938, 2068.0781, 2074.9868, 2075.9924, 2085.9863, 2090.9866, 2125.0095, 2150.0178, 2153.0398, 2167.0352, 2182.0723, 2217.0549, 2218.0278, 2218.0278, 2225.1108, 2230.1848, 2235.0493, 2235.0493, 2276.1116, 2309.0579, 2321.1477, 2498.3943, 2517.4363, 2518.4265, 2518.4265, 2533.4321, 2535.4402, 2535.4402, 2548.3088, 2557.4385, 2562.3926, 2573.4106, 2628.3413, 2644.3481, 2662.3376, 2691.2817, 2708.3521, 2720.2588, 2748.3000, 2807.3022, 2883.4065, 2929.4556, 3346.6948 |
| **S17 gi|212552206 Mass: 34343 Score: 260 Expect: 1.9e-020 Queries matched: 14 late-embryogenesis abundant protein 2 [Glycine max]**  Observed Mr(expt) Mr(calc) Delta Start End Miss Ions Peptide  1098.6957 1097.6884 1097.6696 0.0189 70 - 79 0 --- K.AELVVDVLIK.N  1310.6487 1309.6414 1309.6125 0.0290 257 - 267 0 --- K.DFGSALWDMIR.G  1326.6500 1325.6427 1325.6074 0.0353 257 - 267 0 26 K.DFGSALWDMIR.G + Oxidation (M)  1326.6500 1325.6427 1325.6074 0.0353 257 - 267 0 --- K.DFGSALWDMIR.G + Oxidation (M)  1328.7865 1327.7792 1327.7499 0.0293 151 - 162 0 --- K.VDLIVDVPVFGR.L  1387.7003 1386.6930 1386.6601 0.0329 277 - 289 0 --- K.GHIDVDTPFGAMK.L  1403.6982 1402.6909 1402.6551 0.0359 277 - 289 0 --- K.GHIDVDTPFGAMK.L + Oxidation (M)  1533.8811 1532.8738 1532.8238 0.0500 191 - 203 0 62 R.FSFEETVAILHLK.L  1533.8811 1532.8738 1532.8238 0.0500 191 - 203 0 --- R.FSFEETVAILHLK.L  1555.8599 1554.8526 1554.9133 -0.0607 149 - 162 1 --- R.VKVDLIVDVPVFGR.L  2142.1848 2141.1775 2141.0251 0.1524 270 - 289 1 --- R.GTGYTFKGHIDVDTPFGAMK.L  2160.2246 2159.2173 2159.1222 0.0951 102 - 122 0 124 K.LVSGLIPDSGTIHAHGEETVK.I  2160.2246 2159.2173 2159.1222 0.0951 102 - 122 0 --- K.LVSGLIPDSGTIHAHGEETVK.I  2663.3972 2662.3899 2662.4329 -0.0430 44 - 69 0 --- K.IEGAVGFGKPTADVTGIHIPSINLEK.A  No match to: 842.5136, 877.0521, 1197.6823, 1258.6534, 1261.6775, 1262.3457, 1262.6359, 1266.6345, 1277.7321, 1278.6415, 1286.7714, 1289.7660, 1293.7489, 1294.2272, 1294.6302, 1300.7218, 1309.6541, 1314.6494, 1324.6359, 1330.6461, 1339.6794, 1342.6405, 1342.6405, 1346.6376, 1358.6359, 1358.6359, 1374.6329, 1440.7520, 1448.7167, 1475.7915, 1490.7501, 1491.8018, 1508.8490, 1508.8490, 1509.8464, 1569.9803, 1569.9803, 1591.9583, 1605.9109, 1619.9496, 1689.9507, 1697.9775, 1715.9962, 1716.9910, 1716.9910, 1733.0494, 1734.0173, 1734.0173, 1738.9977, 1749.9960, 1753.9542, 1755.9700, 1771.9647, 1775.9352, 1788.9266, 1800.9829, 1803.0117, 1824.0099, 1841.0419, 1845.9250, 1863.0154, 1878.0668, 1891.0278, 1943.1976, 1994.0359, 2029.0302, 2089.1885, 2091.2256, 2130.2214, 2182.1924, 2197.1389, 2211.2078, 2223.3040, 2223.3040, 2230.2854, 2245.2742, 2246.2893, 2261.2356, 2331.3203, 2332.3135, 2348.3474, 2349.3342, 2349.3342, 2351.8965, 2371.3245, 2384.0361, 2387.2173, 2459.3691, 2477.4294, 2477.4294, 2658.3726, 2691.3645, 2695.3762, 2705.2646, 2748.4001, 2807.4128, 3312.4304, 3346.8462 |
| **S30 gi|6103623 Mass: 41564 Score: 637 Expect: 3.7e-058 Queries matched: 25 actin [Picea rubens]**  Observed Mr(expt) Mr(calc) Delta Start End Miss Ions Peptide  976.3809 975.3736 975.4410 -0.0673 21 - 30 0 --- K.AGFAGDDAPR.A  1132.4514 1131.4441 1131.5196 -0.0755 199 - 208 0 --- R.GYSFTTTAER.E  1176.4606 1175.4533 1175.5427 -0.0893 42 - 52 0 --- R.HTGVMVGMGQK.D + 2 Oxidation (M)  1198.6320 1197.6247 1197.6982 -0.0734 31 - 41 0 52 R.AVFPSIVGRPR.H  1198.6320 1197.6247 1197.6982 -0.0734 31 - 41 0 --- R.AVFPSIVGRPR.H  1459.5989 1458.5916 1458.6739 -0.0823 362 - 374 0 79 K.AEYDESGPSIVHR.K  1459.5989 1458.5916 1458.6739 -0.0823 362 - 374 0 --- K.AEYDESGPSIVHR.K  1515.6697 1514.6624 1514.7419 -0.0794 87 - 97 0 62 K.IWHHTFYNELR.V  1515.6697 1514.6624 1514.7419 -0.0794 87 - 97 0 --- K.IWHHTFYNELR.V  1587.6898 1586.6825 1586.7688 -0.0863 362 - 375 1 --- K.AEYDESGPSIVHRK.C  1744.7927 1743.7854 1743.8791 -0.0937 194 - 208 1 --- K.ILTERGYSFTTTAER.E  1747.7988 1746.7915 1746.8788 -0.0872 241 - 256 0 102 K.SYELPDGQVITIGAER.F  1747.7988 1746.7915 1746.8788 -0.0872 241 - 256 0 --- K.SYELPDGQVITIGAER.F  1855.8403 1854.8330 1854.9250 -0.0920 218 - 233 0 --- K.LAYVALDYEQELETAK.S  1932.8016 1931.7943 1931.8723 -0.0780 71 - 86 0 --- K.YPIEHGIVSNWDDMEK.I  1948.7932 1947.7859 1947.8672 -0.0813 71 - 86 0 --- K.YPIEHGIVSNWDDMEK.I + Oxidation (M)  1953.9819 1952.9746 1953.0571 -0.0824 98 - 115 0 85 R.VAPEEHPVLLTEAPLNPK.A  1953.9819 1952.9746 1953.0571 -0.0824 98 - 115 0 --- R.VAPEEHPVLLTEAPLNPK.A  2182.9785 2181.9712 2182.0728 -0.1015 294 - 314 0 164 K.DLYGNIVLSGGSTMFPGIADR.M  2182.9785 2181.9712 2182.0728 -0.1015 294 - 314 0 --- K.DLYGNIVLSGGSTMFPGIADR.M  2198.9941 2197.9868 2198.0677 -0.0809 294 - 314 0 (28) K.DLYGNIVLSGGSTMFPGIADR.M + Oxidation (M)  2198.9941 2197.9868 2198.0677 -0.0809 294 - 314 0 --- K.DLYGNIVLSGGSTMFPGIADR.M + Oxidation (M)  2311.0559 2310.0486 2310.1677 -0.1191 293 - 314 1 --- R.KDLYGNIVLSGGSTMFPGIADR.M  2327.0654 2326.0581 2326.1626 -0.1045 293 - 314 1 --- R.KDLYGNIVLSGGSTMFPGIADR.M + Oxidation (M)  3151.5208 3150.5135 3150.6349 -0.1213 150 - 179 0 --- R.TTGIVLDSGDGVSHTVPIYEGYALPHAILR.L  No match to: 945.4846, 1042.5319, 1128.4598, 1156.4727, 1181.5876, 1192.4579, 1392.5398, 1429.5879, 1445.5826, 1445.5826, 1450.6090, 1456.6097, 1471.6401, 1475.6050, 1497.6563, 1498.6545, 1501.6564, 1513.6511, 1517.9325, 1519.6669, 1529.6448, 1531.6565, 1531.6565, 1537.6566, 1543.6503, 1547.6488, 1547.6488, 1572.6790, 1576.6727, 1599.7113, 1613.7247, 1637.7002, 1698.7118, 1702.7617, 1729.7906, 1742.8344, 1770.8124, 1771.8146, 1788.8246, 1788.8246, 1802.8210, 1810.8121, 1812.8962, 1820.8435, 1884.7799, 1935.8069, 1980.7960, 2026.8567, 2090.9380, 2116.9697, 2134.9705, 2134.9705, 2142.0059, 2150.9724, 2164.9707, 2169.9841, 2180.9739, 2187.9941, 2193.0437, 2200.5347, 2214.9722, 2225.9709, 2239.9844, 2263.0210, 2335.1528, 2366.1399, 2487.0637, 2533.1797, 2676.1353, 2680.2053, 2691.1738, 2698.2537, 2720.1821, 2748.2004, 2779.2664, 2808.1968, 3087.4758, 3088.4497, 3104.5281, 3107.4661, 3133.5149, 3138.4558, 3150.5928, 3208.5081, 3235.5632, 3249.5752, 3337.6245 |
| **S29 gi|321437427 Mass: 46368 Score: 647 Expect: 3.7e-059 Queries matched: 21 alpha-tubulin [Musa acuminata AAA Group]**  Observed Mr(expt) Mr(calc) Delta Start End Miss Ions Peptide  1007.5159 1006.5086 1006.4468 0.0618 69 - 77 0 --- K.EDAANNFAR.G  1396.8586 1395.8513 1395.7510 0.1003 57 - 68 0 65 R.QLFHPEQLISGK.E  1396.8586 1395.8513 1395.7510 0.1003 57 - 68 0 --- R.QLFHPEQLISGK.E  1473.9626 1472.9553 1472.8562 0.0991 202 - 215 0 121 R.LVSQVISSLTASLR.F  1473.9626 1472.9553 1472.8562 0.0991 202 - 215 0 --- R.LVSQVISSLTASLR.F  1692.0044 1690.9971 1690.8638 0.1333 188 - 201 0 71 R.SLDIERPTYTNLNR.L  1692.0044 1690.9971 1690.8638 0.1333 188 - 201 0 --- R.SLDIERPTYTNLNR.L  1702.0300 1701.0227 1700.8984 0.1243 37 - 51 0 51 R.AVFVDLEPTVIDEVR.T  1702.0300 1701.0227 1700.8984 0.1243 37 - 51 0 --- R.AVFVDLEPTVIDEVR.T  1793.0614 1792.0541 1791.9229 0.1312 237 - 252 0 --- R.IHFMLSSYAPVISAEK.A  1809.0818 1808.0745 1807.9178 0.1567 237 - 252 0 --- R.IHFMLSSYAPVISAEK.A + Oxidation (M)  1815.0300 1814.0227 1813.9032 0.1195 325 - 342 0 --- K.CGINYQPPSVVPGGDLAK.V  1831.9974 1830.9901 1830.9298 0.0604 78 - 93 1 --- R.GHYTIGKEIVDLCLDR.I  1978.0524 1977.0451 1976.8751 0.1700 13 - 32 0 --- K.TVGGGDDAFNTFFSETGAGK.H  2330.1877 2329.1804 2329.0109 0.1696 375 - 394 0 124 R.AFVHWYVGEGMEEGEFSEAR.E  2330.1877 2329.1804 2329.0109 0.1696 375 - 394 0 --- R.AFVHWYVGEGMEEGEFSEAR.E  2346.2102 2345.2029 2345.0058 0.1971 375 - 394 0 (66) R.AFVHWYVGEGMEEGEFSEAR.E + Oxidation (M)  2346.2102 2345.2029 2345.0058 0.1971 375 - 394 0 --- R.AFVHWYVGEGMEEGEFSEAR.E + Oxidation (M)  2395.3584 2394.3511 2394.1855 0.1656 216 - 236 0 151 R.FDGALNVDVTEFQTNLVPYPR.I  2395.3584 2394.3511 2394.1855 0.1656 216 - 236 0 --- R.FDGALNVDVTEFQTNLVPYPR.I  2657.4631 2656.4558 2656.2148 0.2410 253 - 276 0 --- K.AYHEQLSVAEITNSAFEPSSMMAK.C + Oxidation (M)  No match to: 832.3565, 1132.6365, 1219.5873, 1235.5847, 1237.6517, 1267.5916, 1283.5891, 1299.5889, 1378.8436, 1379.8336, 1418.8207, 1527.8522, 1529.9363, 1531.9683, 1549.8069, 1598.8871, 1614.9038, 1630.9075, 1636.9208, 1647.9343, 1649.9434, 1658.0109, 1674.0033, 1674.9819, 1674.9819, 1678.9822, 1684.0175, 1688.0067, 1701.0172, 1716.0322, 1724.0088, 1745.0414, 1754.9747, 1804.0435, 1810.0447, 1821.0656, 1826.0126, 1827.9342, 1835.0881, 1858.0186, 1858.0186, 1869.0474, 1874.0259, 1874.0259, 1875.9620, 1885.0479, 1886.1019, 1891.9417, 1908.0313, 1914.0825, 1918.0409, 2000.0239, 2017.2354, 2196.2246, 2211.2815, 2225.2783, 2239.3157, 2264.3098, 2282.1887, 2282.1887, 2297.2725, 2298.2207, 2309.3706, 2313.2412, 2328.2688, 2351.2280, 2362.1917, 2366.2554, 2368.2908, 2377.3235, 2378.2805, 2383.1797, 2387.2214, 2394.2810, 2409.3398, 2417.3525, 2425.3076, 2507.4070, 2524.3921, 2559.4692, 2559.4692, 2575.4629, 2593.4614, 2609.4814, 2673.4868, 2691.4802, 2695.4463, 2748.5139, 2807.5325, 3193.8547, 3297.8828 |
| **S18 gi|166343835 Mass: 49980 Score: 618 Expect: 2.9e-056 Queries matched: 31 beta-tubulin 14 [Gossypium hirsutum]**  Observed Mr(expt) Mr(calc) Delta Start End Miss Ions Peptide  1059.5990 1058.5917 1058.5219 0.0699 310 - 318 0 --- R.YLTASAMFR.G  1075.6000 1074.5927 1074.5168 0.0759 310 - 318 0 --- R.YLTASAMFR.G + Oxidation (M)  1077.6083 1076.6010 1076.5250 0.0760 155 - 162 1 --- K.IREEYPDR.M  1139.7751 1138.7678 1138.6862 0.0816 253 - 262 0 59 K.LAVNLIPFPR.L  1139.7751 1138.7678 1138.6862 0.0816 253 - 262 0 --- K.LAVNLIPFPR.L  1146.6750 1145.6677 1145.5829 0.0848 242 - 251 0 46 R.FPGQLNSDLR.K  1146.6750 1145.6677 1145.5829 0.0848 242 - 251 0 --- R.FPGQLNSDLR.K  1215.6736 1214.6663 1214.5753 0.0910 381 - 390 0 60 R.VSEQFTAMFR.R  1215.6736 1214.6663 1214.5753 0.0910 381 - 390 0 --- R.VSEQFTAMFR.R  1231.6735 1230.6662 1230.5703 0.0960 381 - 390 0 (8) R.VSEQFTAMFR.R + Oxidation (M)  1231.6735 1230.6662 1230.5703 0.0960 381 - 390 0 --- R.VSEQFTAMFR.R + Oxidation (M)  1274.7803 1273.7730 1273.6778 0.0952 242 - 252 1 59 R.FPGQLNSDLRK.L  1274.7803 1273.7730 1273.6778 0.0952 242 - 252 1 --- R.FPGQLNSDLRK.L  1342.7412 1341.7339 1341.6313 0.1027 47 - 58 0 --- R.INVYYNEASGGR.Y  1622.9777 1621.9704 1621.8438 0.1266 263 - 276 0 --- R.LHFFMVGFAPLTSR.G  1633.9264 1632.9191 1632.7851 0.1340 63 - 77 0 --- R.AVLMDLEPGTMDSVR.S  1638.9865 1637.9792 1637.8388 0.1405 263 - 276 0 25 R.LHFFMVGFAPLTSR.G + Oxidation (M)  1638.9865 1637.9792 1637.8388 0.1405 263 - 276 0 --- R.LHFFMVGFAPLTSR.G + Oxidation (M)  1649.9327 1648.9254 1648.7800 0.1454 63 - 77 0 --- R.AVLMDLEPGTMDSVR.S + Oxidation (M)  1665.9384 1664.9311 1664.7749 0.1562 63 - 77 0 --- R.AVLMDLEPGTMDSVR.S + 2 Oxidation (M)  1696.9902 1695.9829 1695.8256 0.1573 337 - 350 0 --- K.NSSYFVEWIPNNVK.S  1863.0071 1861.9998 1861.8304 0.1694 20 - 35 0 28 K.FWEVICDEHGIDNTGK.Y  1863.0071 1861.9998 1861.8304 0.1694 20 - 35 0 --- K.FWEVICDEHGIDNTGK.Y  1920.0602 1919.0529 1918.8917 0.1613 363 - 379 0 --- K.MASTFIGNSTSIQEMFR.R  1936.0787 1935.0714 1934.8866 0.1848 363 - 379 0 --- K.MASTFIGNSTSIQEMFR.R + Oxidation (M)  1952.0812 1951.0739 1950.8815 0.1924 363 - 379 0 --- K.MASTFIGNSTSIQEMFR.R + 2 Oxidation (M)  1973.1636 1972.1563 1971.9901 0.1662 104 - 121 0 154 K.GHYTEGAELIDSVLDVVR.K  1973.1636 1972.1563 1971.9901 0.1662 104 - 121 0 --- K.GHYTEGAELIDSVLDVVR.K  2101.2517 2100.2444 2100.0851 0.1594 104 - 122 1 --- K.GHYTEGAELIDSVLDVVRK.E  2814.5952 2813.5879 2813.3673 0.2207 78 - 103 0 100 R.SGLFGQIFRPDNFVFGQSGAGNNWAK.G  2814.5952 2813.5879 2813.3673 0.2207 78 - 103 0 --- R.SGLFGQIFRPDNFVFGQSGAGNNWAK.G  No match to: 1128.6608, 1167.2886, 1167.6586, 1230.7550, 1235.6736, 1236.6893, 1294.7628, 1324.7036, 1354.7423, 1382.7777, 1398.7849, 1408.7675, 1431.7419, 1443.7667, 1445.7616, 1445.7616, 1486.7936, 1497.8276, 1549.8870, 1574.9144, 1574.9144, 1585.9050, 1601.9175, 1656.9424, 1679.9567, 1690.8231, 1803.0875, 1823.0919, 1838.0133, 1845.0337, 1846.0253, 1861.0149, 1867.0007, 1872.0311, 1876.0398, 1879.0054, 1888.0758, 1892.0502, 1895.0045, 1917.0464, 1929.0939, 1956.1166, 1957.1682, 1969.0930, 2003.1622, 2023.1888, 2067.0989, 2147.1289, 2182.2825, 2211.3167, 2225.3008, 2229.3027, 2239.3145, 2241.2561, 2246.3821, 2267.2212, 2269.2183, 2283.2251, 2309.3093, 2315.3130, 2353.3467, 2357.3516, 2455.3718, 2691.5190, 2695.4292, 2752.5498, 2769.5657, 2796.5508, 2800.5603, 2807.5693, 2812.6072, 2818.5903, 2828.5781, 2829.5923, 2830.5872, 2834.5774, 2842.5876, 2846.5791, 2846.5791, 2859.5278, 3347.0308 |
| **S19 gi|332197637 Mass: 50185 Score: 660 Expect: 1.9e-060 Queries matched: 27 tubulin beta-1 chain [Arabidopsis thaliana]**  Observed Mr(expt) Mr(calc) Delta Start End Miss Ions Peptide  1059.4657 1058.4584 1058.5219 -0.0634 311 - 319 0 --- R.YLTASAMFR.G  1075.4620 1074.4547 1074.5168 -0.0621 311 - 319 0 --- R.YLTASAMFR.G + Oxidation (M)  1077.4685 1076.4612 1076.5250 -0.0638 156 - 163 1 --- K.IREEYPDR.M  1139.6276 1138.6203 1138.6862 -0.0659 254 - 263 0 56 K.LAVNLIPFPR.L  1139.6276 1138.6203 1138.6862 -0.0659 254 - 263 0 --- K.LAVNLIPFPR.L  1146.5272 1145.5199 1145.5829 -0.0630 243 - 252 0 40 R.FPGQLNSDLR.K  1146.5272 1145.5199 1145.5829 -0.0630 243 - 252 0 --- R.FPGQLNSDLR.K  1215.5156 1214.5083 1214.5753 -0.0670 382 - 391 0 60 R.VSEQFTAMFR.R  1215.5156 1214.5083 1214.5753 -0.0670 382 - 391 0 --- R.VSEQFTAMFR.R  1231.5138 1230.5065 1230.5703 -0.0637 382 - 391 0 (9) R.VSEQFTAMFR.R + Oxidation (M)  1231.5138 1230.5065 1230.5703 -0.0637 382 - 391 0 --- R.VSEQFTAMFR.R + Oxidation (M)  1274.6171 1273.6098 1273.6778 -0.0680 243 - 253 1 60 R.FPGQLNSDLRK.L  1274.6171 1273.6098 1273.6778 -0.0680 243 - 253 1 --- R.FPGQLNSDLRK.L  1342.5686 1341.5613 1341.6313 -0.0699 48 - 59 0 78 R.INVYYNEASGGR.Y  1342.5686 1341.5613 1341.6313 -0.0699 48 - 59 0 --- R.INVYYNEASGGR.Y  1622.7678 1621.7605 1621.8438 -0.0833 264 - 277 0 --- R.LHFFMVGFAPLTSR.G  1638.7678 1637.7605 1637.8388 -0.0782 264 - 277 0 25 R.LHFFMVGFAPLTSR.G + Oxidation (M)  1638.7678 1637.7605 1637.8388 -0.0782 264 - 277 0 --- R.LHFFMVGFAPLTSR.G + Oxidation (M)  1647.7272 1646.7199 1646.8007 -0.0808 64 - 78 0 --- R.AVLMDLEPGTMDSIR.S  1663.7266 1662.7193 1662.7957 -0.0763 64 - 78 0 --- R.AVLMDLEPGTMDSIR.S + Oxidation (M)  1679.7405 1678.7332 1678.7906 -0.0573 64 - 78 0 --- R.AVLMDLEPGTMDSIR.S + 2 Oxidation (M)  1696.7651 1695.7578 1695.8256 -0.0678 338 - 351 0 --- K.NSSYFVEWIPNNVK.S  1956.9138 1955.9065 1955.9952 -0.0887 105 - 122 0 161 K.GHYTEGAELIDAVLDVVR.K  1956.9138 1955.9065 1955.9952 -0.0887 105 - 122 0 --- K.GHYTEGAELIDAVLDVVR.K  2084.9968 2083.9895 2084.0901 -0.1006 105 - 123 1 --- K.GHYTEGAELIDAVLDVVRK.E  2814.2166 2813.2093 2813.3309 -0.1216 79 - 104 0 112 R.SGPYGQIFRPDNFVFGQSGAGNNWAK.G  2814.2166 2813.2093 2813.3309 -0.1216 79 - 104 0 --- R.SGPYGQIFRPDNFVFGQSGAGNNWAK.G  No match to: 1128.5208, 1167.0283, 1167.1964, 1167.5089, 1197.5919, 1230.5614, 1270.6283, 1294.5911, 1336.5524, 1365.6390, 1369.6091, 1382.6154, 1398.6134, 1431.5581, 1445.5771, 1497.6573, 1531.6771, 1546.6563, 1549.6848, 1573.7549, 1574.6918, 1574.6918, 1585.7101, 1599.7284, 1615.7266, 1633.7217, 1649.7234, 1650.7437, 1656.7285, 1675.6649, 1700.7997, 1820.8585, 1822.8524, 1834.9038, 1838.8407, 1844.7854, 1862.7668, 1866.7646, 1870.8387, 1871.7883, 1876.8147, 1878.7675, 1887.8325, 1894.7612, 1903.8214, 1912.8635, 1916.7898, 1919.8087, 1928.8406, 1935.8252, 1938.8567, 1951.8212, 1954.8461, 1972.9028, 1972.9028, 1994.8848, 2100.9795, 2128.9231, 2160.9504, 2176.9277, 2192.9434, 2211.0010, 2224.9985, 2230.1050, 2266.9407, 2407.1118, 2455.0415, 2592.1047, 2658.1497, 2690.1414, 2705.1204, 2796.1855, 2797.2185, 2800.1995, 2812.2056, 2819.2183, 2826.2000, 2828.2251, 2830.2136, 2830.2136, 2842.2048, 2844.2119, 2846.2085, 3044.3611, 3346.5696 |
| **S3 gi|12659206 Mass: 14133 Score: 89 Expect: 0.0022 Queries matched: 9 minor allergen hazelnut profilin [Corylus avellana]**  Observed Mr(expt) Mr(calc) Delta Start End Miss Ions Peptide  1432.6193 1431.6120 1431.7543 -0.1423 72 - 84 0 71 K.YMVIQGEPGAVIR.R  1432.6193 1431.6120 1431.7543 -0.1423 72 - 84 0 --- K.YMVIQGEPGAVIR.R  1448.6138 1447.6065 1447.7493 -0.1427 72 - 84 0 (50) K.YMVIQGEPGAVIR.R + Oxidation (M)  1448.6138 1447.6065 1447.7493 -0.1427 72 - 84 0 --- K.YMVIQGEPGAVIR.R + Oxidation (M)  2298.9707 2297.9634 2297.9503 0.0131 1 - 19 0 --- -.MSWQTYGDEHLMCEIEGNR.L  2298.9707 2297.9634 2297.9503 0.0131 1 - 19 0 --- -.MSWQTYGDEHLMCEIEGNR.L  2795.1099 2794.1026 2794.3339 -0.2313 97 - 121 0 --- K.TSQALIIGIYDEPMTPGQCNMIVER.L + Oxidation (M)  2811.1064 2810.0991 2810.3288 -0.2297 97 - 121 0 --- K.TSQALIIGIYDEPMTPGQCNMIVER.L + 2 Oxidation (M)  2811.1064 2810.0991 2810.3288 -0.2297 97 - 121 0 --- K.TSQALIIGIYDEPMTPGQCNMIVER.L + 2 Oxidation (M)  No match to: 713.3303, 734.4075, 768.4433, 842.4109, 850.4346, 854.9473, 856.4238, 860.9643, 864.4152, 868.4437, 870.4391, 876.9385, 882.4615, 930.3735, 996.4799, 1045.4434, 1051.5905, 1106.4481, 1126.4387, 1128.4292, 1144.4028, 1165.6154, 1183.5690, 1193.5287, 1334.7540, 1340.5631, 1341.5770, 1342.5809, 1355.5858, 1357.5939, 1367.5558, 1382.6016, 1384.3339, 1384.3339, 1384.6130, 1400.6101, 1400.6101, 1406.5897, 1414.6270, 1415.6190, 1418.6044, 1422.5835, 1428.5645, 1430.6029, 1443.6007, 1447.6095, 1453.6150, 1454.5977, 1462.5935, 1464.5952, 1464.5952, 1470.5802, 1475.6051, 1486.5525, 1489.6123, 1492.5688, 1508.5300, 1575.6442, 1713.7415, 2002.8781, 2181.9397, 2210.9080, 2210.9080, 2220.9258, 2224.9167, 2224.9167, 2229.9819, 2232.9099, 2238.9346, 2246.0103, 2246.0103, 2248.9272, 2260.8931, 2281.9011, 2282.9429, 2291.8230, 2312.9851, 2320.9092, 2663.0869, 2676.9954, 2679.0793, 2682.1267, 2683.1033, 2683.1033, 2692.0393, 2696.0347, 2708.0505, 2713.0454, 2720.0715, 2724.0256, 2731.0823, 2736.0549, 2747.1074, 2747.1074, 2752.0562, 2763.1089, 2779.0378, 2823.0488, 2914.2415, 3330.3635, 3346.3909, 3350.3379, 3352.2778 |
| **S27 gi|149391003 Mass: 26477 Score: 80 Expect: 0.018 Queries matched: 7 adenosine kinase 2, partial [Oryza sativa Indica Group]**  Observed Mr(expt) Mr(calc) Delta Start End Miss Ions Peptide  1213.7145 1212.7072 1212.6502 0.0570 148 - 157 0 --- K.KPENWALVEK.A  1303.6998 1302.6925 1302.6278 0.0647 43 - 53 0 --- K.HLPMYDELASK.G  1319.7062 1318.6989 1318.6227 0.0762 43 - 53 0 --- K.HLPMYDELASK.G + Oxidation (M)  1869.9794 1868.9721 1868.9308 0.0413 209 - 224 0 66 K.VLPFVDYIFGNETEAR.I  1869.9794 1868.9721 1868.9308 0.0413 209 - 224 0 --- K.VLPFVDYIFGNETEAR.I  1934.0225 1933.0152 1932.9630 0.0523 188 - 203 0 --- K.VFLMNLSAPFICEFFR.D  1934.0225 1933.0152 1932.9630 0.0523 188 - 203 0 --- K.VFLMNLSAPFICEFFR.D  No match to: 871.4477, 912.6235, 1039.7310, 1158.6775, 1236.6211, 1239.7083, 1255.6938, 1256.7562, 1256.7562, 1260.7549, 1272.7534, 1273.7007, 1277.8513, 1288.7484, 1317.7367, 1321.7317, 1321.7317, 1344.7570, 1344.7570, 1351.7478, 1351.7478, 1374.7648, 1374.7648, 1384.7025, 1386.7323, 1388.7291, 1396.6975, 1399.8005, 1404.7759, 1404.7759, 1410.7776, 1428.6853, 1431.7566, 1445.7325, 1461.7953, 1470.9005, 1519.8596, 1556.8563, 1731.9500, 1737.9698, 1738.9556, 1739.9537, 1754.9956, 1755.9659, 1755.9659, 1767.9705, 1769.0530, 1800.0195, 1810.0289, 1827.0610, 1828.0015, 1828.0015, 1868.0457, 1875.9690, 1886.0298, 1887.0778, 1891.9773, 1900.0245, 1908.0293, 1911.1274, 1916.0084, 1918.0198, 1918.0198, 1925.1439, 1933.0319, 1944.9917, 1949.0468, 1961.0382, 1968.1674, 1977.0626, 2025.0608, 2041.0657, 2081.2041, 2186.2559, 2188.2654, 2209.2585, 2229.1528, 2230.1851, 2302.1882, 2310.1155, 2320.1335, 2320.1335, 2343.2637, 2359.2595, 2396.3022, 2425.3408, 2564.4773, 2663.5469, 2691.4570, 2748.4856, 2807.4973, 2909.4714, 2943.5264, 2956.5986, 2982.5476, 2999.6104, 3000.5500, 3057.6326, 3157.8762, 3159.7695, 3203.8242, 3220.8223, 3231.8359, 3248.8445, 3491.9729 |
| **S28 gi|82400168 Mass: 37549 Score: 113 Expect: 9.3e-006 Queries matched: 6 adenosine kinase isoform 1T-like protein [Solanum tuberosum**]  Observed Mr(expt) Mr(calc) Delta Start End Miss Ions Peptide  1739.7450 1738.7377 1738.8526 -0.1148 229 - 243 0 --- K.VHGWETDNVEEIALK.I  1811.8468 1810.8395 1810.9676 -0.1281 257 - 274 0 --- R.ITVITQGADPVVVAEDGK.V  1875.7236 1874.7163 1874.8508 -0.1345 209 - 224 0 96 K.ALPYMDFVFGNETEAR.I + Oxidation (M)  1875.7236 1874.7163 1874.8508 -0.1345 209 - 224 0 --- K.ALPYMDFVFGNETEAR.I + Oxidation (M)  1967.9506 1966.9433 1967.0687 -0.1254 256 - 274 1 --- K.RITVITQGADPVVVAEDGK.V  2159.9641 2158.9568 2159.0139 -0.0571 318 - 337 1 --- R.AGCYASNVIIQRSGCTYPEK.T  No match to: 871.3336, 1039.5964, 1185.4626, 1238.5870, 1239.5721, 1254.5739, 1255.5549, 1256.5983, 1256.5983, 1260.5959, 1272.5927, 1273.5200, 1284.5848, 1288.5824, 1303.5377, 1319.5332, 1321.5641, 1321.5641, 1351.5735, 1351.5735, 1378.6208, 1386.5692, 1396.5198, 1410.6008, 1465.7380, 1470.7198, 1500.6095, 1755.7389, 1784.6969, 1799.8177, 1809.7500, 1825.7295, 1827.7212, 1827.7212, 1841.7919, 1843.7238, 1857.7430, 1860.7161, 1873.7294, 1874.7296, 1886.8263, 1889.7638, 1890.7799, 1891.7289, 1891.7289, 1907.7432, 1910.7905, 1911.8409, 1912.7555, 1912.7555, 1917.7548, 1918.7401, 1919.7410, 1932.7429, 1934.7532, 1959.8285, 1960.7876, 1960.7876, 1975.8689, 1976.7964, 1976.7964, 1992.7854, 2002.8217, 2006.7924, 2008.7987, 2017.7982, 2024.8025, 2024.8025, 2040.8031, 2040.8031, 2056.7979, 2081.8047, 2208.9951, 2211.9075, 2228.0068, 2228.8477, 2262.8193, 2275.8130, 2301.8389, 2317.8755, 2319.8291, 2319.8291, 2321.3826, 2333.8506, 2335.8579, 2360.8538, 2379.0129, 2544.0127, 2678.1030, 2691.0713, 2800.3206, 2807.1038, 3129.4336, 3132.3352, 3176.4253, 3202.4487, 3203.3872, 3218.3921, 3220.4099, 3233.3884, 3242.3223, 3260.4116, 3277.3679, 3322.4392, 3347.4326, 3477.5444 |
| **S47 gi|160690776 Mass: 14017 Score: 220 Expect: 1.9e-016 Queries matched: 6 malate dehydrogenase, partial [Citrus trifoliata]**  Observed Mr(expt) Mr(calc) Delta Start End Miss Ions Peptide  1238.4999 1237.4926 1237.6012 -0.1086 53 - 63 0 --- K.EVIEAMASFNK.K  1496.8319 1495.8246 1495.9085 -0.0839 34 - 48 0 91 K.VIKPTILIGSSGVGR.T  1496.8319 1495.8246 1495.9085 -0.0839 34 - 48 0 --- K.VIKPTILIGSSGVGR.T  1846.7872 1845.7799 1845.8573 -0.0774 92 - 108 0 --- R.AIFASGSPFDPFEYNGK.V  2096.9990 2095.9917 2096.0803 -0.0885 16 - 33 0 105 K.KPWAHEHEPVNNLLDAVK.V  2096.9990 2095.9917 2096.0803 -0.0885 16 - 33 0 --- K.KPWAHEHEPVNNLLDAVK.V  No match to: 701.2820, 733.2725, 742.3365, 752.3862, 774.3315, 801.3476, 842.4468, 855.0113, 870.9862, 877.0049, 979.4523, 1012.4422, 1046.5297, 1060.0209, 1064.5863, 1066.5449, 1147.6106, 1157.5824, 1174.6193, 1174.6193, 1221.5597, 1222.5085, 1258.6168, 1270.5055, 1276.6279, 1276.6279, 1286.4980, 1290.6365, 1292.5966, 1298.6089, 1302.4924, 1315.6405, 1315.6405, 1339.6216, 1461.5409, 1471.5177, 1478.7528, 1487.5576, 1488.5396, 1488.5396, 1519.5232, 1535.5166, 1536.5367, 1536.5367, 1551.5118, 1552.5398, 1552.5398, 1559.6769, 1568.5438, 1593.5912, 1611.6249, 1611.6249, 1616.8538, 1620.8525, 1638.6761, 1642.8252, 1658.7999, 1704.7778, 1734.7858, 1736.8427, 1800.8599, 1868.7813, 1902.9169, 1953.8630, 1964.9935, 1964.9935, 1993.8728, 2017.8726, 2033.9558, 2100.9968, 2112.9934, 2118.9761, 2128.9849, 2211.0115, 2219.0559, 2225.0298, 2230.0952, 2239.0269, 2320.1631, 2324.0464, 2328.0420, 2374.0881, 2393.0872, 2421.1184, 2428.1157, 2436.0947, 2452.1184, 2477.0732, 2480.1323, 2480.1323, 2484.1350, 2496.1223, 2512.1194, 2512.1194, 2663.1980, 2691.1675, 2700.3262, 2705.1418, 2720.1582, 2748.1794, 2777.3811, 2807.1816, 2859.3914, 2931.3127, 3162.4695, 3346.5532 |
| **S31 gi|295291644 Mass: 63583 Score: 286 Expect: 4.6e-023 Queries matched: 16 ketol-acid reductoisomerase [Catharanthus roseus]**  Observed Mr(expt) Mr(calc) Delta Start End Miss Ions Peptide  823.3240 822.3168 822.3871 -0.0704 305 - 311 0 --- K.SDIFGER.G  1046.4471 1045.4398 1045.5266 -0.0868 417 - 426 0 --- K.EGLPAFPMGK.I  1062.4431 1061.4358 1061.5215 -0.0857 417 - 426 0 --- K.EGLPAFPMGK.I + Oxidation (M)  1399.6487 1398.6414 1398.6561 -0.0147 49 - 63 0 --- R.AASFTTSCGSAVAAR.M  1680.8557 1679.8484 1679.9722 -0.1238 312 - 327 0 98 R.GILLGAVHGIVESLFR.R  1680.8557 1679.8484 1679.9722 -0.1238 312 - 327 0 --- R.GILLGAVHGIVESLFR.R  1695.7249 1694.7176 1694.8548 -0.1372 359 - 373 1 --- K.GMLAVYNALTEEEKK.E  1695.7249 1694.7176 1694.8548 -0.1372 359 - 373 1 --- K.GMLAVYNALTEEEKK.E  1711.7229 1710.7156 1710.8498 -0.1341 359 - 373 1 --- K.GMLAVYNALTEEEKK.E + Oxidation (M)  1711.7229 1710.7156 1710.8498 -0.1341 359 - 373 1 --- K.GMLAVYNALTEEEKK.E + Oxidation (M)  1735.7372 1734.7299 1734.8822 -0.1522 1 - 19 0 --- -.MAAAAATSISVSTAPAATK.T + Oxidation (M)  1836.9391 1835.9318 1836.0733 -0.1415 312 - 328 1 --- R.GILLGAVHGIVESLFRR.Y  2085.8611 2084.8538 2084.9875 -0.1336 258 - 277 0 159 K.EINGAGINSSFAVHQDVDGR.A  2085.8611 2084.8538 2084.9875 -0.1336 258 - 277 0 --- K.EINGAGINSSFAVHQDVDGR.A  2931.2461 2930.2388 2930.3868 -0.1480 474 - 499 0 --- K.GHSYSEIINESVIESVDSLNPFMHAR.G  2947.2346 2946.2273 2946.3817 -0.1544 474 - 499 0 --- K.GHSYSEIINESVIESVDSLNPFMHAR.G + Oxidation (M)  No match to: 842.4349, 899.3835, 960.3886, 998.4464, 1198.5635, 1358.5580, 1365.6042, 1390.5872, 1445.5618, 1464.6536, 1483.5695, 1496.5760, 1496.5760, 1515.6313, 1531.6063, 1543.6960, 1544.6093, 1544.6093, 1545.5939, 1547.5879, 1560.5691, 1560.5691, 1576.6190, 1601.6896, 1602.6926, 1619.7266, 1619.7266, 1626.7620, 1638.7358, 1639.6667, 1647.7103, 1663.7211, 1671.7813, 1677.6886, 1678.6962, 1687.6705, 1693.7286, 1694.7594, 1703.6658, 1708.8044, 1718.7379, 1731.7227, 1737.8134, 1747.7588, 1763.6649, 1788.7838, 1819.8201, 1827.8041, 1953.9252, 2067.8506, 2067.8506, 2068.8464, 2075.8826, 2116.9131, 2122.9651, 2139.9514, 2147.8240, 2185.9746, 2187.9651, 2203.9717, 2210.9636, 2238.9851, 2374.1118, 2392.1482, 2454.9800, 2483.1638, 2557.1045, 2591.1636, 2689.1235, 2692.1086, 2720.1042, 2737.1606, 2748.1155, 2753.1509, 2807.1216, 2883.2026, 2883.2026, 2920.3193, 2929.2659, 2929.2659, 2932.2368, 2933.2446, 2961.2678, 2961.2678, 2984.3887, 3011.2988, 3034.3777, 3039.3672, 3050.3467, 3066.3447, 3074.2739, 3162.1687, 3210.1538, 3226.2092, 3527.5762, 3555.5620 |
| **S 14 gi|291162645 Mass: 29404 Score: 241 Expect: 1.5e-018 Queries matched: 7 14-3-3 family protein [Dimocarpus longan]**  Observed Mr(expt) Mr(calc) Delta Start End Miss Ions Peptide  1418.5627 1417.5554 1417.6936 -0.1382 37 - 48 0 --- K.TVDVEELTVEER.N  1716.7319 1715.7246 1715.8941 -0.1694 34 - 48 1 --- K.VAKTVDVEELTVEER.N  1802.8032 1801.7959 1801.9686 -0.1727 160 - 176 0 93 K.SAQDIALAELAPTHPIR.L  1802.8032 1801.7959 1801.9686 -0.1727 160 - 176 0 --- K.SAQDIALAELAPTHPIR.L  2121.8762 2120.8689 2120.9750 -0.1060 232 - 250 0 --- R.DNLTLWTSDITDDAGDEIK.E  2330.9949 2329.9876 2330.1946 -0.2070 177 - 196 0 128 R.LGLALNFSVFYYEILNSPDR.A  2330.9949 2329.9876 2330.1946 -0.2070 177 - 196 0 --- R.LGLALNFSVFYYEILNSPDR.A  No match to: 816.3356, 842.4168, 844.3982, 945.4744, 1011.4207, 1024.4255, 1061.3923, 1110.4712, 1112.5081, 1112.5081, 1140.4888, 1141.5074, 1189.5398, 1205.5347, 1205.5347, 1277.6573, 1279.5872, 1309.5408, 1321.6626, 1345.4207, 1360.5558, 1364.5543, 1371.5720, 1376.5538, 1399.6271, 1408.5610, 1424.5524, 1440.5469, 1456.5430, 1492.6282, 1518.6382, 1518.6382, 1590.7076, 1635.7428, 1641.7330, 1645.5889, 1657.6508, 1680.6969, 1725.7380, 1760.6783, 1775.6877, 1777.7017, 1777.7017, 1793.6935, 1799.6709, 1815.6516, 1831.7450, 1864.7506, 1880.7404, 1886.8088, 1887.8224, 1902.7942, 1904.8495, 1904.8495, 1910.8419, 1926.8301, 1929.7794, 1936.8522, 1957.8624, 1957.8624, 1962.8044, 1966.8488, 1977.7928, 1993.7880, 1997.7817, 2000.8436, 2036.8479, 2036.8479, 2057.8633, 2060.8542, 2080.8828, 2113.8909, 2120.8921, 2171.8445, 2181.8982, 2185.9412, 2210.9202, 2224.9341, 2229.9993, 2232.9453, 2238.9453, 2245.9958, 2256.9424, 2269.8831, 2273.8833, 2298.9792, 2345.0142, 2345.0142, 2351.8608, 2374.9490, 2375.9683, 2390.9380, 2392.9531, 2392.9531, 2414.9595, 2421.0137, 2427.0459, 2430.9656, 2449.9558, 2463.9746, 2463.9746, 2479.8840, 2723.0789, 3050.1011, 3346.4155 |
| **S 9 gi|255584432 Mass: 25994 Score: 191 Expect: 1.5e-013 Queries matched: 11 proteasome subunit alpha type, putative [Ricinus communis]**  Observed Mr(expt) Mr(calc) Delta Start End Miss Ions Peptide  825.4193 824.4120 824.4504 -0.0384 87 - 93 0 --- R.TLVEHAR.V  883.3979 882.3906 882.4308 -0.0401 94 - 100 0 --- R.VETQNHR.F  1063.4755 1062.4682 1062.5094 -0.0411 11 - 20 0 65 R.GVNTFSPEGR.L  1063.4755 1062.4682 1062.5094 -0.0411 11 - 20 0 --- R.GVNTFSPEGR.L  1098.5499 1097.5426 1097.5829 -0.0403 208 - 217 0 --- K.VTPNNVDIAR.V  1399.7266 1398.7193 1398.7606 -0.0413 54 - 66 0 --- R.ITSPLLEPSSVEK.V  1423.7460 1422.7387 1422.7758 -0.0371 21 - 32 0 83 R.LFQVEYAIEAIK.L  1423.7460 1422.7387 1422.7758 -0.0371 21 - 32 0 --- R.LFQVEYAIEAIK.L  1940.8617 1939.8544 1939.8759 -0.0214 167 - 185 0 --- K.AIGSGSEGADSSLQEQYNK.D  2487.1206 2486.1133 2486.3553 -0.2419 186 - 207 1 --- K.DITLLEAETIALSILKQVMEEK.V  2503.1257 2502.1184 2502.3502 -0.2318 186 - 207 1 --- K.DITLLEAETIALSILKQVMEEK.V + Oxidation (M)  No match to: 709.3345, 725.3290, 842.4697, 1045.4894, 1195.4413, 1259.4436, 1439.2841, 1439.5546, 1439.5546, 1445.7206, 1461.6934, 1485.5901, 1487.5680, 1490.7379, 1503.5680, 1503.5680, 1602.7417, 1603.8080, 1649.8036, 1677.8024, 1757.9581, 1779.9310, 1795.9097, 1829.8364, 1972.8713, 1986.9602, 2078.9656, 2086.0273, 2105.9773, 2115.0452, 2127.9795, 2141.0679, 2142.0742, 2152.9001, 2153.9871, 2158.0864, 2159.0745, 2159.0745, 2170.0256, 2170.0256, 2174.0637, 2181.0591, 2186.0154, 2197.0759, 2201.9910, 2211.0820, 2216.0815, 2218.0239, 2218.0239, 2225.0828, 2228.1155, 2231.1624, 2234.0225, 2234.0225, 2239.0879, 2250.0676, 2254.0806, 2256.1326, 2272.1501, 2272.1501, 2273.6814, 2283.1257, 2288.1265, 2294.0972, 2310.0928, 2313.1128, 2329.1482, 2350.0879, 2397.1794, 2400.0881, 2414.1458, 2423.0994, 2439.1567, 2441.1265, 2441.1265, 2449.1270, 2455.1541, 2459.1406, 2489.1309, 2489.1309, 2505.1506, 2505.1506, 2521.1094, 2527.1382, 2532.1108, 2546.1384, 2662.3018, 2691.2317, 2695.2224, 2709.2205, 2720.2493, 2721.2290, 2724.2446, 2748.2520, 2764.2615, 2807.2568, 2823.2686, 2914.4712, 3290.7251, 3338.6860, 3346.6304 |
| **S 8 gi|124484511 Mass: 21911 Score: 242 Expect: 1.2e-018 Queries matched: 8 alpha chain of nascent polypeptide associated complex [Nicotiana benthamiana]**  Observed Mr(expt) Mr(calc) Delta Start End Miss Ions Peptide  1154.7423 1153.7350 1153.6641 0.0709 73 - 83 0 41 K.LGMKPIPGVSR.V  1154.7423 1153.7350 1153.6641 0.0709 73 - 83 0 --- K.LGMKPIPGVSR.V  1170.7405 1169.7332 1169.6590 0.0742 73 - 83 0 (30) K.LGMKPIPGVSR.V + Oxidation (M)  1170.7405 1169.7332 1169.6590 0.0742 73 - 83 0 --- K.LGMKPIPGVSR.V + Oxidation (M)  1520.0001 1518.9928 1518.8809 0.1119 91 - 103 0 56 K.NILFVISKPDVFK.S  1520.0001 1518.9928 1518.8809 0.1119 91 - 103 0 --- K.NILFVISKPDVFK.S  1936.1322 1935.1249 1934.9585 0.1664 118 - 134 0 137 K.IEDLSSQLQTQAAEQFK.A  1936.1322 1935.1249 1934.9585 0.1664 118 - 134 0 --- K.IEDLSSQLQTQAAEQFK.A  No match to: 713.4592, 768.5732, 842.5514, 870.5857, 882.6126, 1045.6251, 1106.2971, 1106.2971, 1106.7349, 1126.6316, 1152.7300, 1179.6825, 1369.8181, 1378.8180, 1380.5625, 1380.5625, 1380.8318, 1383.8330, 1396.8419, 1408.8322, 1411.8423, 1426.8422, 1428.8494, 1428.8494, 1442.8424, 1444.8496, 1444.8496, 1449.8569, 1456.8402, 1460.8411, 1466.8318, 1471.8440, 1475.8492, 1496.8472, 1501.9426, 1514.8494, 1536.8442, 1541.9808, 1552.8263, 1557.9480, 1650.9690, 1707.9561, 1767.9218, 1789.0254, 1919.1161, 1949.1293, 1950.1190, 1958.1116, 1963.1312, 1967.1328, 1967.1328, 1974.0868, 1977.1201, 1989.1279, 1994.1113, 2003.2174, 2005.1080, 2011.1213, 2180.2117, 2182.2942, 2182.2942, 2211.2888, 2219.2852, 2225.3174, 2229.3271, 2230.3723, 2230.3723, 2239.3289, 2246.3816, 2246.3816, 2283.3308, 2292.2578, 2297.2751, 2299.3547, 2313.3640, 2324.2410, 2384.1299, 2406.3174, 2419.3994, 2476.4353, 2665.4619, 2678.4929, 2691.4885, 2695.4976, 2696.4790, 2705.3760, 2717.3223, 2720.4385, 2723.4988, 2748.5103, 2752.5012, 2764.5403, 2768.5208, 2780.5286, 2807.5203, 2914.7354, 2997.0212, 3329.9595, 3346.9746, 3353.9673, 3376.9214, 3440.9277, 3452.9617, 3468.9377 |
| **S 12 gi|226505926 Mass: 24767 Score: 219 Expect: 2.3e-016 Queries matched: 11 elongation factor 1-delta 1 [Zea mays]**  Observed Mr(expt) Mr(calc) Delta Start End Miss Ions Peptide  716.3586 715.3514 715.3864 -0.0350 159 - 164 0 --- K.LEEAVR.S  1022.5124 1021.5051 1021.5444 -0.0393 17 - 24 0 50 K.LDEYLLTR.S  1022.5124 1021.5051 1021.5444 -0.0393 17 - 24 0 --- K.LDEYLLTR.S  1117.5144 1116.5071 1116.5451 -0.0380 25 - 34 0 --- R.SYITGYQASK.D  1150.6096 1149.6023 1149.6393 -0.0370 16 - 24 1 70 K.KLDEYLLTR.S  1150.6096 1149.6023 1149.6393 -0.0370 16 - 24 1 --- K.KLDEYLLTR.S  1405.6786 1404.6713 1404.7071 -0.0358 165 - 177 0 --- R.SVQMEGLLWGASK.L  1421.6766 1420.6693 1420.7020 -0.0327 165 - 177 0 70 R.SVQMEGLLWGASK.L + Oxidation (M)  1421.6766 1420.6693 1420.7020 -0.0327 165 - 177 0 --- R.SVQMEGLLWGASK.L + Oxidation (M)  2332.1345 2331.1272 2331.2660 -0.1387 165 - 186 1 --- R.SVQMEGLLWGASKLVPVGYGIK.K  2348.0955 2347.0882 2347.2609 -0.1727 165 - 186 1 --- R.SVQMEGLLWGASKLVPVGYGIK.K + Oxidation (M)  No match to: 713.3596, 768.4968, 820.4082, 832.2725, 837.4579, 842.4693, 844.4520, 855.0349, 871.0082, 877.0204, 882.5269, 917.5055, 939.4988, 955.4821, 1060.0457, 1108.4901, 1133.6140, 1147.5470, 1190.5887, 1197.6216, 1208.5531, 1208.5531, 1354.6639, 1356.6758, 1357.6736, 1357.6736, 1361.6678, 1371.7003, 1373.6758, 1389.6652, 1402.6401, 1425.6779, 1430.6967, 1431.6857, 1433.7052, 1437.6726, 1443.6550, 1448.7040, 1448.7040, 1453.6686, 1470.6842, 1486.6654, 1576.7960, 1582.6671, 1616.7987, 1654.8071, 1693.7794, 1701.7814, 1787.8917, 1882.9902, 1929.9288, 1958.0012, 1975.9377, 1977.9327, 1977.9327, 1993.9319, 1998.9342, 2009.9280, 2015.9233, 2025.9192, 2058.0115, 2058.0115, 2082.8694, 2106.0088, 2122.0288, 2122.0288, 2138.0122, 2162.0388, 2208.1013, 2211.0928, 2225.0928, 2230.1545, 2238.0403, 2239.0571, 2292.0896, 2299.1543, 2308.1470, 2310.1433, 2310.1433, 2331.1494, 2401.1707, 2424.9790, 2441.1548, 2458.1719, 2535.0801, 2553.0693, 2553.0693, 2569.3120, 2569.3120, 2575.1274, 2591.2524, 2594.1960, 2607.2366, 2658.2229, 2691.2441, 2720.2292, 2748.2642, 2771.3496, 2807.2722, 3091.4170, 3348.5698 |
| **S 15 gi|195646972 Mass: 93862 Score: 143 Expect: 9.3e-009 Queries matched: 8 elongation factor 2 [Zea mays]**  Observed Mr(expt) Mr(calc) Delta Start End Miss Ions Peptide  744.3888 743.3815 743.3754 0.0061 392 - 397 0 --- R.FFAFGR.V  1358.6331 1357.6258 1357.6006 0.0253 233 - 243 1 --- K.FGVDETKMMER.L + Oxidation (M)  1453.5963 1452.5890 1452.6442 -0.0552 831 - 842 0 --- K.EQMTPLSEFEDK.L  1469.6069 1468.5996 1468.6391 -0.0395 831 - 842 0 --- K.EQMTPLSEFEDK.L + Oxidation (M)  1469.6069 1468.5996 1468.6391 -0.0395 831 - 842 0 --- K.EQMTPLSEFEDK.L + Oxidation (M)  2256.9858 2255.9785 2256.1961 -0.2175 33 - 55 0 133 K.STLTDSLVAAAGIIAQEVAGDVR.M  2256.9858 2255.9785 2256.1961 -0.2175 33 - 55 0 --- K.STLTDSLVAAAGIIAQEVAGDVR.M  2931.1819 2930.1746 2930.4409 -0.2663 94 - 120 0 --- R.DGNQYLINLIDSPGHVDFSSEVTAALR.I  No match to: 779.9317, 795.9045, 832.9800, 842.4085, 854.9642, 864.1978, 870.9381, 872.9437, 876.9586, 892.9308, 984.9313, 985.4226, 985.4226, 1007.3793, 1025.3549, 1025.3549, 1029.3491, 1041.3477, 1052.4263, 1057.3394, 1059.9606, 1059.9606, 1063.9606, 1065.9827, 1081.9603, 1097.9346, 1110.4609, 1116.3943, 1179.4744, 1184.4287, 1260.4883, 1260.4883, 1264.4829, 1270.5596, 1270.9869, 1276.4755, 1302.5043, 1336.5405, 1360.5095, 1367.5839, 1368.5559, 1405.5671, 1418.5778, 1421.5769, 1421.5769, 1468.6017, 1472.6127, 1475.6041, 1485.5959, 1485.5959, 1492.6180, 1502.7147, 1607.7615, 1638.6268, 1649.6533, 1657.6367, 1679.7251, 1707.6040, 1716.6874, 1737.7258, 1737.7258, 1747.7200, 1776.7310, 1777.7468, 1782.6869, 1791.5764, 1794.6865, 1821.7069, 1827.7539, 1833.7245, 1920.8147, 1993.7810, 2002.8054, 2027.7401, 2040.8191, 2043.8153, 2052.8452, 2056.8071, 2057.8306, 2059.7896, 2059.7896, 2071.7874, 2073.8181, 2139.9355, 2167.8682, 2185.9292, 2185.9292, 2191.7935, 2210.9089, 2224.9243, 2229.9783, 2245.9954, 2278.9749, 2280.9758, 2328.9731, 2383.7244, 2414.9333, 2603.9956, 2646.0994, 2646.0994, 2704.9109, 2716.8289, 2945.1985, 3346.4126 |
| **S 36 gi|217038830 Mass: 17387 Score: 153 Expect: 9.3e-010 Queries matched: 10 eukaryotic translation initiation factor 5A1 [Glycine max]**  Observed Mr(expt) Mr(calc) Delta Start End Miss Ions Peptide  1118.6526 1117.6453 1117.5880 0.0573 19 - 28 0 57 K.TFPQQAGTIR.K  1118.6526 1117.6453 1117.5880 0.0573 19 - 28 0 --- K.TFPQQAGTIR.K  1134.6450 1133.6377 1133.6292 0.0086 42 - 52 1 --- K.VVEVSTSKTGK.H  1134.6450 1133.6377 1133.6292 0.0086 42 - 52 1 --- K.VVEVSTSKTGK.H  1246.7489 1245.7416 1245.6829 0.0587 19 - 29 1 --- K.TFPQQAGTIRK.N  1370.8362 1369.8289 1369.7453 0.0837 117 - 128 0 --- R.LPTDENLLSQIK.D  1870.0980 1869.0907 1868.9843 0.1064 113 - 128 1 60 K.DDLRLPTDENLLSQIK.D  1870.0980 1869.0907 1868.9843 0.1064 113 - 128 1 --- K.DDLRLPTDENLLSQIK.D  2075.1343 2074.1270 2074.0582 0.0688 117 - 135 1 --- R.LPTDENLLSQIKDGFAEGK.D  2075.1343 2074.1270 2074.0582 0.0688 117 - 135 1 --- R.LPTDENLLSQIKDGFAEGK.D  No match to: 712.2749, 713.4423, 723.4113, 739.3614, 758.4239, 767.2178, 773.2246, 780.0735, 789.1828, 796.0481, 832.3411, 833.1242, 842.5416, 855.1128, 859.1116, 861.1246, 870.5741, 871.0894, 873.0948, 874.0817, 877.1014, 882.6031, 890.4998, 893.0776, 985.1101, 1007.0978, 1060.1508, 1060.1508, 1066.1649, 1074.5214, 1082.1427, 1098.1206, 1100.6399, 1116.6438, 1120.4618, 1140.6351, 1156.6168, 1197.7103, 1201.7079, 1213.7117, 1229.7047, 1271.2151, 1286.5988, 1327.8453, 1362.7382, 1379.7611, 1384.7867, 1384.7867, 1388.7797, 1389.7477, 1401.7388, 1406.7736, 1406.7736, 1417.7185, 1428.7458, 1444.7310, 1526.8960, 1590.8772, 1638.9036, 1654.9105, 1724.9504, 1776.0028, 1868.0225, 2015.1268, 2047.1014, 2047.1014, 2056.2009, 2058.1619, 2088.1296, 2088.1296, 2092.1543, 2104.1636, 2112.1775, 2120.2095, 2125.2090, 2143.2217, 2157.2080, 2170.1970, 2175.2043, 2175.2043, 2186.2295, 2198.2168, 2203.2405, 2203.2405, 2211.2749, 2216.2324, 2216.2324, 2225.2822, 2230.3303, 2239.2788, 2246.3513, 2260.2512, 2273.2466, 2399.1909, 2659.5334, 2665.4170, 2672.5388, 2678.4929, 2691.4751, 2710.4707, 2807.4934, 3346.9724 |
| **S 40 gi|82621136 Mass: 29831 Score: 101 Expect: 0.00015 Queries matched: 9 eukaryotic translation initiation factor 2 beta subunit-like [Solanum tuberosum]**  Observed Mr(expt) Mr(calc) Delta Start End Miss Ions Peptide  761.4828 760.4755 760.4595 0.0160 120 - 125 0 --- R.VFNILR.E  848.4787 847.4714 847.4552 0.0163 202 - 208 0 --- K.NFEGILR.R  1196.7262 1195.7189 1195.6859 0.0330 138 - 147 0 --- R.TVMRPPQVLR.E  1212.7258 1211.7185 1211.6808 0.0377 138 - 147 0 --- R.TVMRPPQVLR.E + Oxidation (M)  1286.6271 1285.6198 1285.5826 0.0372 110 - 119 0 78 R.DYEYEELLGR.V  1286.6271 1285.6198 1285.5826 0.0372 110 - 119 0 --- R.DYEYEELLGR.V  1444.7747 1443.7674 1443.7254 0.0421 152 - 163 1 --- K.KTVFVNFMDLCK.T  2230.2314 2229.2241 2229.0437 0.1805 2 - 21 0 --- M.AEEENQNELVADIAPFDPTK.K  2246.1694 2245.1621 2245.0758 0.0863 210 - 229 1 --- R.YVNEYVICNGCKSPDTILSK.E  No match to: 774.3870, 842.5228, 862.4926, 877.4698, 908.4764, 937.5316, 981.4885, 981.4885, 997.6011, 1009.4609, 1076.5341, 1136.6398, 1152.6477, 1152.6477, 1293.6409, 1344.7627, 1359.7614, 1362.8164, 1375.7651, 1391.7570, 1408.7434, 1459.8104, 1464.7878, 1550.8597, 1564.8613, 1576.8848, 1579.8827, 1591.8831, 1592.8721, 1593.8834, 1594.8845, 1594.8845, 1596.4255, 1598.2113, 1600.8715, 1601.8558, 1604.8608, 1608.8732, 1610.8748, 1616.8591, 1618.8522, 1618.8522, 1622.8533, 1632.8381, 1634.8435, 1634.8435, 1640.8433, 1644.8618, 1646.8566, 1648.8392, 1650.8339, 1650.8339, 1656.8832, 1666.8392, 1668.8632, 1684.8698, 1690.8888, 1737.8872, 1740.9161, 1758.9673, 1758.9673, 1780.9408, 1796.9244, 1797.9813, 1828.0292, 1989.1003, 2115.2205, 2143.2324, 2143.2324, 2165.1409, 2165.1409, 2181.1538, 2206.1260, 2210.1699, 2211.1992, 2223.1506, 2224.1292, 2224.1292, 2228.1353, 2231.2717, 2235.2268, 2239.2004, 2240.1470, 2256.1223, 2262.0847, 2338.2817, 2342.2942, 2390.3247, 2406.3201, 2473.3450, 2491.3760, 2491.3760, 2535.3291, 2552.4077, 2599.4041, 2600.4019, 2616.4321, 2691.4033, 2807.4302, 3010.6262, 3022.7202, 3345.8269, 3347.8635 |
| **S 34 gi|19702 Mass: 17353 Score: 120 Expect: 1.9e-006 Queries matched: 10 eukaryotic initiation factor 5A (2) [Nicotiana plumbaginifolia]**  Observed Mr(expt) Mr(calc) Delta Start End Miss Ions Peptide  1134.6472 1133.6399 1133.5829 0.0570 19 - 28 0 54 K.TYPQQAGTIR.K  1134.6472 1133.6399 1133.6292 0.0108 42 - 52 1 --- K.VVEVSTSKTGK.H  1262.7498 1261.7425 1261.6779 0.0647 19 - 29 1 --- K.TYPQQAGTIRK.N  1370.8374 1369.8301 1369.7453 0.0848 117 - 128 0 --- R.LPTDDNLLTQIK.D  1389.7500 1388.7427 1388.7445 -0.0018 37 - 49 1 --- K.GRPCKVVEVSTSK.T  1870.1130 1869.1057 1868.9843 0.1214 113 - 128 1 --- K.DDLRLPTDDNLLTQIK.D  2075.1399 2074.1326 2074.0582 0.0744 117 - 135 1 --- R.LPTDDNLLTQIKDGFAEGK.D  2075.1399 2074.1326 2074.0582 0.0744 117 - 135 1 --- R.LPTDDNLLTQIKDGFAEGK.D  2159.2200 2158.2127 2158.0589 0.1538 70 - 88 1 --- K.KLEDIVPSSHNCDVPHVNR.T  2672.5430 2671.5357 2671.2864 0.2493 89 - 112 0 --- R.TDYQLIDISEDGFVSLLTENGNTK.D  No match to: 712.2780, 842.5418, 870.5757, 882.6033, 1074.4152, 1074.6158, 1090.3567, 1090.5769, 1100.6427, 1116.6367, 1118.6543, 1118.6543, 1140.6396, 1156.6304, 1246.7513, 1286.5988, 1322.7297, 1361.7533, 1362.7372, 1371.8501, 1379.7649, 1379.7649, 1384.7738, 1388.7654, 1401.7474, 1405.7560, 1406.7749, 1406.7749, 1417.7302, 1428.7603, 1444.7393, 1490.8331, 1715.9420, 1724.9595, 1788.9731, 1868.0408, 1871.1245, 1984.1104, 1997.1139, 2013.1387, 2015.1274, 2015.1274, 2029.1077, 2047.1031, 2047.1031, 2056.1926, 2058.1563, 2070.1521, 2088.1372, 2088.1372, 2092.1650, 2093.1929, 2104.1782, 2112.1875, 2120.2166, 2125.2161, 2140.2034, 2142.2031, 2143.2375, 2143.2375, 2156.2151, 2170.1997, 2175.2092, 2175.2092, 2181.2202, 2182.2319, 2185.2268, 2186.2375, 2193.2271, 2196.2444, 2198.2339, 2199.2192, 2200.2324, 2203.2473, 2203.2473, 2211.2766, 2215.2664, 2216.2375, 2216.2375, 2225.2869, 2230.3230, 2233.2888, 2239.2766, 2244.2661, 2246.3477, 2299.3601, 2313.3682, 2655.4854, 2659.5103, 2663.5149, 2678.5239, 2691.4834, 2694.4966, 2695.4822, 2710.4756, 2720.4622, 2723.4644, 2748.4954, 2752.4937, 2807.5027, 2839.5225, 3346.9773 |
| **S 23 gi|197312901 Mass: 16419 Score: 188 Expect: 2.9e-013 Queries matched: 7 translation initiation factor [Rheum australe]**  Observed Mr(expt) Mr(calc) Delta Start End Miss Ions Peptide  897.3963 896.3890 896.3698 0.0192 95 - 101 0 --- K.YMPDEAR.L + Oxidation (M)  1307.6655 1306.6582 1306.6153 0.0429 30 - 40 0 75 K.EDGQEYAQVLR.M  1307.6655 1306.6582 1306.6153 0.0429 30 - 40 0 --- K.EDGQEYAQVLR.M  1495.9479 1494.9406 1494.8922 0.0485 69 - 82 0 --- K.VWIAAGDIILVGLR.D  1624.0490 1623.0417 1622.9871 0.0546 68 - 82 1 --- K.KVWIAAGDIILVGLR.D  1924.0594 1923.0521 1922.9737 0.0784 25 - 40 1 91 R.ELVFKEDGQEYAQVLR.M  1924.0594 1923.0521 1922.9737 0.0784 25 - 40 1 --- R.ELVFKEDGQEYAQVLR.M  No match to: 709.3903, 712.3670, 713.4280, 717.3904, 719.3837, 720.3677, 723.3921, 725.4027, 727.4472, 737.4304, 743.4528, 752.3907, 764.3990, 768.5474, 780.0447, 782.4901, 796.0156, 802.0218, 821.4261, 833.4197, 842.5249, 850.5527, 855.0885, 861.0982, 864.3306, 870.5583, 871.0646, 877.0759, 882.5843, 893.0493, 930.5034, 996.6207, 1007.0500, 1045.5856, 1051.7338, 1060.1139, 1066.1287, 1075.3391, 1082.1050, 1082.1050, 1098.0822, 1122.5736, 1122.5736, 1126.5941, 1149.3101, 1165.7771, 1249.6321, 1289.6541, 1291.6626, 1334.9380, 1340.7252, 1371.7394, 1371.7394, 1418.8457, 1448.9851, 1477.9238, 1481.9294, 1493.9102, 1499.9456, 1499.9456, 1509.9229, 1509.9229, 1511.9341, 1525.7858, 1527.9342, 1527.9342, 1610.0157, 1618.1428, 1628.0370, 1638.0087, 1640.0345, 1656.0406, 1732.1940, 1780.9141, 1797.9031, 1797.9031, 1858.8967, 1906.0298, 2003.1727, 2180.0977, 2182.1855, 2211.2246, 2225.2388, 2225.2388, 2230.2991, 2233.2434, 2239.2456, 2246.3179, 2246.3179, 2249.2554, 2281.2249, 2283.2292, 2292.1624, 2299.2947, 2299.2947, 2313.2839, 2470.3254, 2691.3726, 2720.3667, 2748.4148, 2752.3921, 2807.4556, 3329.8120, 3346.8374, 3428.7256 |
| **S 6 gi|115187479 Mass: 19116 Score: 286 Expect: 4.6e-023 Queries matched: 9 translationally controlled tumor-like protein [Arachis hypogaea]**  Observed Mr(expt) Mr(calc) Delta Start End Miss Ions Peptide  1063.5660 1062.5587 1062.5709 -0.0122 67 - 75 0 69 K.VVDIVDTFR.L  1063.5660 1062.5587 1062.5709 -0.0122 67 - 75 0 --- K.VVDIVDTFR.L  1075.5310 1074.5237 1074.5345 -0.0108 76 - 84 0 --- R.LQEQPAFDK.K  1203.6278 1202.6205 1202.6295 -0.0090 76 - 85 1 --- R.LQEQPAFDKK.Q  1515.7732 1514.7659 1514.7616 0.0043 22 - 34 0 68 K.EIENGILWEVEGK.W  1515.7732 1514.7659 1514.7616 0.0043 22 - 34 0 --- K.EIENGILWEVEGK.W  2447.2424 2446.2351 2446.1977 0.0374 1 - 21 0 --- -.MLVYQDLLTGDELLSDSFPYK.E  2463.2666 2462.2593 2462.1926 0.0667 1 - 21 0 123 -.MLVYQDLLTGDELLSDSFPYK.E + Oxidation (M)  2463.2666 2462.2593 2462.1926 0.0667 1 - 21 0 --- -.MLVYQDLLTGDELLSDSFPYK.E + Oxidation (M)  No match to: 713.4011, 768.5183, 842.4929, 856.5083, 868.5313, 870.5258, 882.5501, 1013.5350, 1029.5316, 1045.5491, 1051.6976, 1165.7374, 1169.6154, 1231.6366, 1334.8911, 1398.6945, 1439.8356, 1439.8356, 1448.9225, 1465.6156, 1503.7410, 1519.7581, 1531.7693, 1537.7446, 1621.7097, 1621.7097, 1625.8062, 1694.8383, 1724.8885, 1724.8885, 1742.8470, 1746.8617, 1758.8427, 1758.8427, 1762.8402, 1780.8197, 1861.9330, 2000.9001, 2003.0826, 2182.2136, 2183.1616, 2193.1079, 2194.1084, 2207.0959, 2210.1162, 2211.1394, 2222.1497, 2225.1523, 2225.1523, 2230.2202, 2233.1809, 2235.1340, 2239.1577, 2246.2312, 2246.2312, 2249.1711, 2282.1484, 2283.2056, 2292.0667, 2296.0991, 2299.1951, 2299.1951, 2313.2197, 2322.1250, 2399.2961, 2417.2822, 2421.1934, 2434.1902, 2448.2300, 2485.2256, 2501.2256, 2564.2307, 2662.3684, 2666.2715, 2676.3049, 2678.3430, 2691.3127, 2691.3127, 2695.2937, 2704.3376, 2707.2832, 2720.2856, 2723.3064, 2724.2957, 2748.3318, 2748.3318, 2752.3379, 2764.3208, 2780.3442, 2792.3770, 2793.3464, 2807.3254, 2811.3315, 2839.3193, 2841.3440, 2857.3601, 2914.5244, 3255.6084, 3289.7878, 3329.7329, 3337.7551, 3346.7515, 3353.7458 |
| **S 39 gi|255569484 Mass: 84668 Score: 373 Expect: 9.3e-032 Queries matched: 19 5-methyltetrahydropteroyltriglutamate--homocysteine methyltransferase, putative [Ricinus communis]**  Observed Mr(expt) Mr(calc) Delta Start End Miss Ions Peptide  734.4490 733.4417 733.4486 -0.0069 679 - 684 0 --- K.LLSVFR.E  1096.5676 1095.5603 1095.5712 -0.0109 287 - 296 0 --- K.YLFAGVVDGR.N  1130.5626 1129.5553 1129.5615 -0.0062 705 - 714 0 --- R.IPSTEEIADR.I  1197.6151 1196.6078 1196.6189 -0.0111 260 - 270 0 --- K.GVTAYGFDLVR.G  1412.7551 1411.7478 1411.7394 0.0084 2 - 14 1 --- M.ASHIVGYPRMGPK.R  1412.7551 1411.7478 1411.7394 0.0084 2 - 14 1 --- M.ASHIVGYPRMGPK.R  1482.8221 1481.8148 1481.8201 -0.0053 597 - 610 0 109 K.AGINVIQIDEAALR.E  1482.8221 1481.8148 1481.8201 -0.0053 597 - 610 0 --- K.AGINVIQIDEAALR.E  1543.7463 1542.7390 1542.7799 -0.0408 1 - 14 1 --- -.MASHIVGYPRMGPK.R  1658.8252 1657.8179 1657.8212 -0.0033 689 - 704 0 96 K.YGAGIGPGVYDIHSPR.I  1658.8252 1657.8179 1657.8212 -0.0033 689 - 704 0 --- K.YGAGIGPGVYDIHSPR.I  1806.9415 1805.9342 1805.9498 -0.0155 556 - 571 0 --- K.GMLTGPVTILNWSFVR.N + Oxidation (M)  1822.9044 1821.8971 1821.9658 -0.0687 739 - 755 1 --- R.KYSEVKPALSNMVAAAK.L + Oxidation (M)  1984.0355 1983.0282 1982.9638 0.0644 617 - 632 1 --- R.KSEQAFYLDWAVHSFR.I  1991.0090 1990.0017 1990.0007 0.0011 477 - 493 0 113 K.LQEELDIDVLVHGEPER.N  1991.0090 1990.0017 1990.0007 0.0011 477 - 493 0 --- K.LQEELDIDVLVHGEPER.N  2175.0515 2174.0442 2174.0937 -0.0495 718 - 736 0 --- K.MLAVLEMNILWVNPDCGLK.T + Oxidation (M)  2324.1106 2323.1033 2323.3151 -0.2118 427 - 447 0 --- K.LNLPILPTTTIGSFPQTIELR.R  2720.2759 2719.2686 2719.4544 -0.1858 297 - 321 1 --- R.NIWANDLAASLTILHELEGIVGKDK.L  No match to: 842.4940, 932.4745, 943.4518, 996.4769, 1041.5360, 1087.5687, 1133.6204, 1149.6167, 1229.6368, 1292.5983, 1309.7042, 1325.6212, 1424.7701, 1424.7701, 1444.7709, 1444.7709, 1474.6882, 1490.7347, 1503.7922, 1521.8536, 1521.8536, 1530.7264, 1558.6758, 1635.8412, 1640.8315, 1648.8519, 1648.8519, 1653.8864, 1685.8680, 1691.8467, 1711.9087, 1718.7908, 1724.7808, 1728.9408, 1728.9408, 1738.8551, 1742.8981, 1746.8695, 1754.8280, 1781.8407, 1802.8688, 1810.9524, 1818.8643, 1825.8644, 1826.9380, 1829.8424, 1834.8943, 1845.8348, 1864.8715, 1864.8715, 1868.8752, 1880.8873, 1890.0040, 1896.8624, 1908.8804, 1968.0222, 1972.9824, 2032.0282, 2101.9998, 2140.1606, 2147.0852, 2157.0366, 2168.1670, 2178.0732, 2192.0566, 2198.0935, 2199.0718, 2211.1279, 2219.1445, 2219.1445, 2225.1309, 2230.1809, 2242.0862, 2246.1404, 2250.1316, 2252.1028, 2342.1238, 2342.1238, 2386.2310, 2404.1890, 2419.2522, 2434.2424, 2450.2285, 2459.2856, 2609.2158, 2613.2173, 2650.3420, 2691.2732, 2695.2808, 2748.3052, 2752.3069, 2871.4460, 2952.4495 |
| **S 43 gi|332197185 Mass: 47680 Score: 138 Expect: 2.9e-008 Queries matched: 5 alanine aminotransferase 2 [Arabidopsis thaliana]**  Observed Mr(expt) Mr(calc) Delta Start End Miss Ions Peptide  1518.5851 1517.5778 1517.6820 -0.1042 367 - 380 0 --- R.GGYMEVTGFTSDVR.E  1800.7540 1799.7467 1799.8777 -0.1309 410 - 423 1 --- K.KEMEFSHPWLNVQR.L  1816.7584 1815.7511 1815.8726 -0.1215 410 - 423 1 --- K.KEMEFSHPWLNVQR.L + Oxidation (M)  2219.0420 2218.0347 2218.1705 -0.1358 280 - 300 0 131 R.ALVVINPGNPTGQVLAEENQR.D  2219.0420 2218.0347 2218.1705 -0.1358 280 - 300 0 --- R.ALVVINPGNPTGQVLAEENQR.D  No match to: 791.3800, 908.3791, 972.4634, 978.4199, 1008.3898, 1044.3677, 1071.5250, 1074.5164, 1105.5111, 1106.4709, 1107.4692, 1128.4224, 1128.4224, 1154.4730, 1170.4668, 1259.4860, 1293.5928, 1296.6053, 1296.6053, 1300.6013, 1312.6033, 1328.5913, 1353.5389, 1386.5387, 1390.5426, 1453.6417, 1454.5920, 1472.5951, 1475.6613, 1502.5862, 1502.5862, 1537.6366, 1539.6178, 1539.6178, 1564.5083, 1564.5083, 1601.7316, 1613.7069, 1618.6659, 1619.6826, 1630.7308, 1637.7167, 1637.7167, 1680.7244, 1729.6495, 1752.7430, 1756.7516, 1763.7521, 1804.8027, 1804.8027, 1811.7466, 1819.7500, 1825.7759, 1836.8579, 1842.8466, 1853.8855, 1853.8855, 1864.8021, 1869.8446, 1870.8390, 1887.8661, 1887.8661, 1891.8668, 1892.8672, 1902.8317, 1903.8466, 1912.8163, 1919.8525, 1928.8221, 1960.8297, 1976.8307, 1992.8318, 2071.9224, 2087.8391, 2161.8381, 2178.8496, 2178.8496, 2194.8562, 2201.9946, 2213.0488, 2220.5447, 2230.0125, 2259.9233, 2290.9583, 2307.0161, 2307.9670, 2308.9763, 2308.9763, 2323.9463, 2365.9880, 2398.0754, 2435.1370, 2609.2468, 2623.2512, 2627.2437, 2637.2249, 2655.2415, 2690.3074, 2692.1951, 2697.1826, 2735.2053, 2739.3018, 2754.2937, 2944.2749, 2965.2715, 2985.3044, 3013.2429 |
| **S 41 gi|1655578 Mass: 42977 Score: 241 Expect: 1.5e-018 Queries matched: 19 S-adenosyl-L-methionine synthetase 2 [Catharanthus roseus]**  Observed Mr(expt) Mr(calc) Delta Start End Miss Ions Peptide  979.4460 978.4388 978.4671 -0.0284 365 - 373 0 --- K.TAAYGHFGR.E  1141.5809 1140.5736 1140.6040 -0.0303 228 - 237 0 53 K.TIFHLNPSGR.F  1141.5809 1140.5736 1140.6040 -0.0303 228 - 237 0 --- K.TIFHLNPSGR.F  1220.5870 1219.5797 1219.6560 -0.0763 61 - 70 1 --- K.AQVDYEKIVR.D  1220.5870 1219.5797 1219.6560 -0.0763 61 - 70 1 --- K.AQVDYEKIVR.D  1453.7242 1452.7169 1452.7473 -0.0304 238 - 252 0 113 R.FVIGGPHGDAGLTGR.K  1453.7242 1452.7169 1452.7473 -0.0304 238 - 252 0 --- R.FVIGGPHGDAGLTGR.K  1702.8361 1701.8288 1701.8613 -0.0325 374 - 387 0 --- R.EDPDFTWEVVKPLK.F  1738.8447 1737.8374 1737.7879 0.0495 75 - 91 0 --- R.AIGFVSDDVGLDADNCK.V  1738.8447 1737.8374 1737.7879 0.0495 75 - 91 0 --- R.AIGFVSDDVGLDADNCK.V  1963.9530 1962.9457 1962.9587 -0.0130 254 - 273 0 --- K.IIIDTYGGWGAHGGGAFSGK.D  1992.9888 1991.9815 1992.0138 -0.0323 339 - 355 0 --- K.ENFDFRPGMIAINLDLK.R  2067.9902 2066.9829 2066.9255 0.0575 1 - 18 0 --- -.METFLFTSESVNEGHPDK.L  2069.9714 2068.9641 2068.9887 -0.0246 170 - 187 0 --- K.TQVTVEYYNDNGAMVPIR.V  2085.9705 2084.9632 2084.9836 -0.0204 170 - 187 0 --- K.TQVTVEYYNDNGAMVPIR.V + Oxidation (M)  2149.0898 2148.0825 2148.1149 -0.0324 339 - 356 1 --- K.ENFDFRPGMIAINLDLKR.G  2165.1099 2164.1026 2164.1098 -0.0072 339 - 356 1 --- K.ENFDFRPGMIAINLDLKR.G + Oxidation (M)  2304.0820 2303.0747 2303.1177 -0.0430 48 - 67 1 --- K.TNMVMVFGEITTKAQVDYEK.I  2649.3442 2648.3369 2648.3293 0.0076 188 - 211 0 --- R.VHTVLISTQHDETVTNDEIAADLK.E  No match to: 952.4979, 968.4973, 1043.6034, 1105.6063, 1224.5811, 1236.5865, 1252.5730, 1265.5925, 1281.6271, 1281.6271, 1285.5885, 1291.6638, 1306.6583, 1324.6040, 1324.6040, 1340.6001, 1356.5934, 1386.7003, 1400.6442, 1404.7192, 1404.7192, 1420.6373, 1435.6979, 1452.6510, 1457.7352, 1458.7413, 1505.7469, 1521.7472, 1525.7474, 1528.7379, 1537.7448, 1553.7445, 1579.8209, 1627.8297, 1643.8356, 1662.9309, 1670.8450, 1720.8177, 1756.8918, 1762.8990, 1772.8845, 1827.8955, 1881.8271, 1939.1033, 1944.9749, 1974.9640, 1990.9265, 2007.9520, 2021.9628, 2021.9628, 2029.9684, 2045.9813, 2055.9539, 2061.9456, 2071.9727, 2101.0598, 2109.9441, 2116.0378, 2125.9460, 2132.0457, 2194.0508, 2200.0632, 2217.0815, 2217.0815, 2218.6001, 2230.1431, 2247.9299, 2272.0938, 2272.0938, 2276.0952, 2288.0903, 2417.2505, 2418.2581, 2518.2910, 2531.2834, 2536.3022, 2536.3022, 2550.2793, 2604.3040, 2612.3154, 2621.3157, 2622.3052, 2622.3052, 2731.4875, 2768.4033, 2870.4690, 2941.4500, 3397.4934, 3455.6624, 3459.6895, 3487.7000, 3554.7957, 3600.5515 |
| **S 42 gi|255562088 Mass: 50396 Score: 244 Expect: 7.4e-019 Queries matched: 10 Transaminase mtnE, putative [Ricinus communis]**  Observed Mr(expt) Mr(calc) Delta Start End Miss Ions Peptide  773.3339 772.3266 772.3980 -0.0713 439 - 445 0 --- R.VSAFGHR.D  870.3649 869.3576 869.4395 -0.0819 305 - 312 0 --- K.YAGFTGVR.L  1322.5818 1321.5745 1321.6917 -0.1172 321 - 332 0 --- K.ELLFSDGFPVAK.D  1377.6381 1376.6308 1376.7452 -0.1144 67 - 78 0 80 K.LQAGYLFPEIAR.R  1377.6381 1376.6308 1376.7452 -0.1144 67 - 78 0 --- K.LQAGYLFPEIAR.R  1854.7612 1853.7539 1853.9311 -0.1772 321 - 336 1 --- K.ELLFSDGFPVAKDFNR.I  1972.8346 1971.8273 1971.9802 -0.1529 419 - 438 0 136 K.THVVTTPGSGFGPGGEGFIR.V  1972.8346 1971.8273 1971.9802 -0.1529 419 - 438 0 --- K.THVVTTPGSGFGPGGEGFIR.V  2011.8197 2010.8124 2010.9469 -0.1345 233 - 251 0 --- R.TDIIFFCSPNNPTGSAATR.E  2230.9841 2229.9768 2230.0501 -0.0733 115 - 135 1 --- K.RSQALSTLEGYSGYGAEQGEK.L  No match to: 842.4310, 1013.4478, 1056.4115, 1060.4856, 1096.5883, 1141.5110, 1155.5387, 1162.5190, 1174.5299, 1176.5463, 1205.4379, 1235.5450, 1253.4343, 1269.4314, 1289.5912, 1292.5775, 1304.6051, 1314.5640, 1361.6322, 1415.6049, 1433.6096, 1433.6096, 1437.6034, 1447.6013, 1449.6110, 1453.6290, 1465.5975, 1471.5841, 1484.5422, 1489.5981, 1500.5594, 1537.6062, 1553.5988, 1639.7556, 1660.7211, 1664.6803, 1680.6920, 1694.6965, 1723.7120, 1738.7441, 1759.6360, 1766.7108, 1768.6755, 1783.6776, 1784.6897, 1784.6897, 1788.7023, 1814.7417, 1818.7733, 1826.7803, 1830.7484, 1832.7820, 1846.7529, 1851.6926, 1862.7499, 1877.7678, 1878.7013, 1878.7013, 1894.7566, 1905.7849, 1909.7257, 1919.8104, 1924.7146, 1926.6354, 1926.6354, 1942.6385, 1942.6385, 1961.8162, 1977.8337, 1993.8275, 1994.8644, 1995.8217, 1995.8217, 2008.8101, 2029.8506, 2041.8273, 2043.8293, 2052.8276, 2052.8276, 2059.8357, 2071.8450, 2075.8320, 2100.8281, 2123.8750, 2159.9595, 2160.9395, 2160.9395, 2187.9221, 2206.9380, 2208.9495, 2215.8577, 2224.9570, 2224.9570, 2240.9443, 2430.9419, 2478.9385, 2490.9937, 2490.9937, 2495.9697, 2629.9951, 2647.0925, 2927.2217 |
| **S 13 gi|332643299 Mass: 14120 Score: 133 Expect: 9.3e-008 Queries matched: 4 glycine-rich RNA-binding protein 4 [Arabidopsis thaliana]**  Observed Mr(expt) Mr(calc) Delta Start End Miss Ions Peptide  713.3554 712.3481 712.3036 0.0445 1 - 6 0 --- -.MAFCNK.L  1681.7476 1680.7403 1680.8358 -0.0955 37 - 52 0 118 K.LFVGGLSWGTDDSSLK.Q  1681.7476 1680.7403 1680.8358 -0.0955 37 - 52 0 --- K.LFVGGLSWGTDDSSLK.Q  2533.0144 2532.0071 2532.1948 -0.1876 13 - 36 1 --- R.QGVSQSSNGPVTSMLGSLRYMSSK.L + 2 Oxidation (M)  No match to: 768.4702, 773.3434, 842.4394, 870.4716, 882.4937, 888.3560, 1045.4808, 1051.6302, 1063.4819, 1116.3315, 1116.3315, 1116.4722, 1140.5131, 1141.4977, 1141.4977, 1151.6444, 1158.5229, 1158.5229, 1165.6639, 1172.4932, 1180.4983, 1320.4972, 1410.5771, 1428.5737, 1428.5737, 1449.6583, 1450.5922, 1475.6206, 1478.7147, 1492.6003, 1508.5947, 1537.6208, 1571.5157, 1571.5157, 1577.7600, 1589.5258, 1589.5258, 1611.5081, 1647.8243, 1663.7386, 1667.7625, 1679.7386, 1685.7454, 1695.7450, 1697.7443, 1703.7192, 1707.6841, 1713.7322, 1719.7024, 1735.7195, 1764.7949, 1838.8054, 1880.7653, 1993.8568, 2002.9235, 2007.8583, 2018.7399, 2026.8851, 2036.7404, 2036.7404, 2050.8018, 2066.9568, 2066.9568, 2211.0005, 2225.0061, 2230.0562, 2233.0374, 2239.0120, 2246.0891, 2249.0486, 2283.0557, 2295.9919, 2299.0693, 2313.0662, 2383.8159, 2664.1638, 2691.1211, 2705.0432, 2716.9500, 2720.0835, 2748.1494, 2764.1260, 2807.1663, 2808.1523, 2811.1460, 2824.1414, 2839.1841, 2859.0559, 2860.1523, 2877.1873, 2877.1873, 2880.0791, 2907.1768, 2908.1194, 2923.1460, 2925.1316, 2925.1316, 2937.1580, 2940.1694, 2941.1653, 2941.1653, 2955.1858, 2957.1296, 2963.1816, 2980.1550, 3337.5286, 3346.5432, 3353.5259 |
| **S 21 gi|7024451 Mass: 16839 Score: 227 Expect: 3.7e-017 Queries matched: 10 glycine-rich RNA-binding protein [Citrus unshiu]**  Observed Mr(expt) Mr(calc) Delta Start End Miss Ions Peptide  930.3957 929.3884 929.4759 -0.0875 50 - 57 0 57 R.GFGFVTFR.D  930.3957 929.3884 929.4759 -0.0875 50 - 57 0 --- R.GFGFVTFR.D  1025.3387 1024.3314 1024.4321 -0.1007 88 - 100 0 --- R.GSGGGGGGGYGSR.G  1131.4683 1130.4610 1130.5680 -0.1069 78 - 87 0 43 R.NITVNEAQSR.G  1131.4683 1130.4610 1130.5680 -0.1069 78 - 87 0 --- R.NITVNEAQSR.G  1302.5280 1301.5207 1301.6404 -0.1196 50 - 60 1 48 R.GFGFVTFRDEK.S  1302.5280 1301.5207 1301.6404 -0.1196 50 - 60 1 --- R.GFGFVTFRDEK.S  1489.5383 1488.5310 1488.6626 -0.1316 64 - 77 0 --- R.DAIEGMNGQNLDGR.N  1505.5352 1504.5279 1504.6576 -0.1296 64 - 77 0 52 R.DAIEGMNGQNLDGR.N + Oxidation (M)  1505.5352 1504.5279 1504.6576 -0.1296 64 - 77 0 --- R.DAIEGMNGQNLDGR.N + Oxidation (M)  No match to: 794.2703, 842.4211, 870.4511, 912.3802, 913.3760, 915.3705, 922.3350, 922.3350, 928.3693, 929.3654, 936.3480, 936.3480, 944.4041, 952.3661, 968.3414, 978.3853, 1039.3541, 1096.4734, 1165.4747, 1179.4863, 1184.5023, 1197.5255, 1213.5188, 1234.5612, 1277.5880, 1284.5255, 1285.5208, 1320.4647, 1340.6090, 1365.5176, 1368.5533, 1383.5615, 1400.5515, 1434.6394, 1441.5234, 1441.5234, 1445.4995, 1475.6190, 1479.6156, 1487.5415, 1491.5483, 1493.5970, 1521.5270, 1657.6510, 1672.5634, 1707.6277, 1716.7042, 1733.6492, 1744.6875, 1758.6620, 1783.5757, 1783.5757, 1788.7147, 1797.6772, 1810.6816, 1811.6449, 1814.6466, 1814.6466, 1818.7310, 1825.7360, 1828.6592, 1828.6592, 1835.7891, 1839.7433, 1842.7030, 1850.6688, 1853.6996, 1856.7002, 1876.7676, 1890.7993, 1923.7241, 1929.7687, 1946.7396, 1950.7184, 1953.7290, 1967.7178, 1967.7178, 1993.8096, 1998.7687, 2012.7822, 2012.7822, 2025.7919, 2034.7776, 2103.8770, 2210.9348, 2224.9504, 2229.9910, 2232.9424, 2240.9712, 2245.9929, 2298.9958, 2367.0586, 2383.7673, 2420.9668, 2692.0654, 2720.0916, 2782.2004, 2825.0923, 3102.2451, 3132.2087, 3164.2402, 3346.4556 |
| **S 44 gi|255571035 Mass: 16301 Score: 136 Expect: 4.6e-008 Queries matched: 4 nucleoside diphosphate kinase, putative [Ricinus communis]**  Observed Mr(expt) Mr(calc) Delta Start End Miss Ions Peptide  1661.7633 1660.7560 1660.8606 -0.1046 2 - 15 0 --- M.EQTFIMIKPDGVQR.G  1667.8097 1666.8024 1666.9002 -0.0978 86 - 102 0 127 R.TIIGATNPAQSAPGTIR.G  1667.8097 1666.8024 1666.9002 -0.0978 86 - 102 0 --- R.TIIGATNPAQSAPGTIR.G  1677.7738 1676.7665 1676.8555 -0.0890 2 - 15 0 --- M.EQTFIMIKPDGVQR.G + Oxidation (M)  No match to: 725.3028, 799.3776, 867.4005, 901.4348, 910.4360, 912.4511, 938.4332, 951.5175, 967.4580, 969.5323, 969.5323, 1065.5205, 1188.5116, 1240.5725, 1242.5887, 1258.2805, 1258.5811, 1274.5787, 1288.5806, 1304.5784, 1306.5879, 1306.5879, 1322.5848, 1322.5848, 1338.5746, 1356.6787, 1373.7136, 1373.7136, 1387.6938, 1395.6959, 1484.6390, 1501.8009, 1501.8009, 1515.7739, 1558.8074, 1613.7537, 1621.7789, 1623.7469, 1632.7650, 1637.7827, 1648.7725, 1649.8083, 1652.7754, 1665.8116, 1666.8271, 1669.3513, 1679.7811, 1681.8076, 1682.7811, 1683.7660, 1689.7645, 1694.8021, 1696.7787, 1697.8223, 1700.7429, 1705.7950, 1716.7468, 1730.7946, 1738.7281, 1738.7281, 1745.8021, 1754.7991, 1758.8279, 1760.7950, 1768.8104, 1784.8164, 1786.8217, 1786.8217, 1800.8298, 1802.8286, 1802.8286, 1816.8075, 1817.8315, 1818.8116, 1829.8179, 1832.8124, 1833.8177, 1834.8232, 1834.8232, 1843.7871, 1848.8203, 1850.8295, 1850.8295, 1864.8032, 1866.8110, 1866.8110, 1876.9027, 1882.7666, 1890.7748, 1891.7797, 1893.7787, 1904.7325, 1905.7551, 1906.7963, 1907.7882, 1923.7870, 1927.7848, 1991.8428, 2016.0643, 2034.0823, 2332.2659, 2663.1750, 2691.1223, 2720.1270, 2748.1406, 2806.1975, 3571.9585, 3573.8591 |
| **S 20 gi|4519264 Mass: 54329 Score: 641 Expect: 1.5e-058 Queries matched: 35 vacuolar H+-ATPase B subunit [Citrus unshiu]**  Observed Mr(expt) Mr(calc) Delta Start End Miss Ions Peptide  842.4846 841.4773 841.4406 0.0368 293 - 299 1 --- R.EEVPGRR.G  1045.4753 1044.4680 1044.4876 -0.0196 477 - 484 0 --- K.TLDQYYSR.D  1069.5494 1068.5421 1068.5603 -0.0182 42 - 49 0 50 K.YYEIVNIR.L  1069.5494 1068.5421 1068.5603 -0.0182 42 - 49 0 --- K.YYEIVNIR.L  1088.5573 1087.5500 1087.5695 -0.0195 95 - 104 0 --- K.TPVSLDMLGR.I  1104.5536 1103.5463 1103.5645 -0.0181 95 - 104 0 --- K.TPVSLDMLGR.I + Oxidation (M)  1113.5149 1112.5076 1112.5250 -0.0174 440 - 449 0 64 K.FVAQGAYDSR.N  1113.5149 1112.5076 1112.5250 -0.0174 440 - 449 0 --- K.FVAQGAYDSR.N  1213.4641 1212.4568 1212.4717 -0.0148 225 - 234 0 --- R.DFEENGSMER.V  1229.4592 1228.4519 1228.4666 -0.0147 225 - 234 0 --- R.DFEENGSMER.V + Oxidation (M)  1241.6111 1240.6038 1240.6200 -0.0162 439 - 449 1 --- R.KFVAQGAYDSR.N  1369.7268 1368.7195 1368.7289 -0.0093 83 - 94 0 --- K.FTTVQFTGEVLK.T  1563.8002 1562.7929 1562.7940 -0.0010 68 - 82 0 --- K.AVVQVFEGTSGIDNK.F  1596.8995 1595.8922 1595.9035 -0.0113 365 - 378 0 87 R.QIYPPINVLPSLSR.L  1596.8995 1595.8922 1595.9035 -0.0113 365 - 378 0 --- R.QIYPPINVLPSLSR.L  1715.9260 1714.9187 1714.9253 -0.0066 235 - 249 0 93 R.VTLFLNLANDPTIER.I  1715.9260 1714.9187 1714.9253 -0.0066 235 - 249 0 --- R.VTLFLNLANDPTIER.I  1903.9893 1902.9820 1902.9873 -0.0053 270 - 286 0 --- K.HVLVILTDMSSYADALR.E  1919.9999 1918.9926 1918.9822 0.0104 270 - 286 0 48 K.HVLVILTDMSSYADALR.E + Oxidation (M)  1919.9999 1918.9926 1918.9822 0.0104 270 - 286 0 --- K.HVLVILTDMSSYADALR.E + Oxidation (M)  1939.8809 1938.8736 1938.8821 -0.0085 300 - 315 0 85 R.GYPGYMYTDLAQIYER.A  1939.8809 1938.8736 1938.8821 -0.0085 300 - 315 0 --- R.GYPGYMYTDLAQIYER.A  1955.8944 1954.8871 1954.8771 0.0101 300 - 315 0 (49) R.GYPGYMYTDLAQIYER.A + Oxidation (M)  1955.8944 1954.8871 1954.8771 0.0101 300 - 315 0 --- R.GYPGYMYTDLAQIYER.A + Oxidation (M)  2050.9856 2049.9783 2049.9868 -0.0084 391 - 408 1 89 R.RDHSDVSNQLYANYAIGK.D  2050.9856 2049.9783 2049.9868 -0.0084 391 - 408 1 --- R.RDHSDVSNQLYANYAIGK.D  2340.1504 2339.1431 2339.1783 -0.0352 415 - 435 0 --- K.AVVGEEALSSEDLLYLEFLDK.F  2355.0598 2354.0525 2354.0518 0.0008 2 - 22 0 --- M.GVAQNNVDMEEGTLEVAMEYR.T  2371.0830 2370.0757 2370.0467 0.0290 2 - 22 0 --- M.GVAQNNVDMEEGTLEVAMEYR.T + Oxidation (M)  2387.0779 2386.0706 2386.0416 0.0290 2 - 22 0 --- M.GVAQNNVDMEEGTLEVAMEYR.T + 2 Oxidation (M)  2469.2036 2468.1963 2468.1927 0.0037 138 - 159 0 --- R.TYPEEMIQTGISTIDVMNSIAR.G  2485.2195 2484.2122 2484.1876 0.0246 138 - 159 0 --- R.TYPEEMIQTGISTIDVMNSIAR.G + Oxidation (M)  2501.2302 2500.2229 2500.1825 0.0404 138 - 159 0 --- R.TYPEEMIQTGISTIDVMNSIAR.G + 2 Oxidation (M)  2772.3481 2771.3408 2771.3904 -0.0496 415 - 438 1 --- K.AVVGEEALSSEDLLYLEFLDKFER.K  3454.7603 3453.7530 3453.7415 0.0115 105 - 137 0 --- R.IFNGSGKPIDNGPPILPEAYLDISGSSINPSER.T  No match to: 1139.6731, 1146.5718, 1197.6414, 1215.5651, 1231.5636, 1274.6676, 1408.6525, 1421.8077, 1431.6204, 1445.6388, 1486.6506, 1574.7855, 1578.8835, 1579.8717, 1579.8717, 1591.8478, 1638.8376, 1671.8590, 1693.9106, 1700.8856, 1702.8534, 1721.9089, 1834.9601, 1854.9229, 1855.9478, 1871.9624, 1887.9257, 1891.8723, 1891.8723, 1907.9019, 1916.8856, 1935.9390, 1937.9150, 1938.9086, 1972.9637, 1993.9478, 2066.9089, 2086.6245, 2087.0994, 2089.0503, 2148.1584, 2160.1375, 2178.1450, 2178.1450, 2179.6565, 2200.1262, 2207.0566, 2211.1057, 2219.1594, 2225.1130, 2230.1836, 2235.1511, 2259.0627, 2267.0222, 2269.0139, 2283.0283, 2306.1013, 2307.0603, 2323.0752, 2353.0955, 2373.1038, 2403.1711, 2419.1636, 2421.1880, 2437.2129, 2658.2820, 2695.1987, 2705.1848, 2733.3708, 2734.3679, 2751.3889, 2751.3889, 2800.3127, 2828.3513, 2832.3420, 2844.3274, 2859.2686 |
| **S 37 gi|296088008 Mass: 17163 Score: 709 Expect: 2.3e-065 Queries matched: 17 unnamed protein product, partial [Vitis vinifera]**  Observed Mr(expt) Mr(calc) Delta Start End Miss Ions Peptide  863.3895 862.3823 862.4548 -0.0726 115 - 121 0 --- K.LAEQFQK.Q  937.3793 936.3720 936.4512 -0.0793 31 - 38 1 32 K.TTTTDDKR.L  937.3793 936.3720 936.4512 -0.0793 31 - 38 1 --- K.TTTTDDKR.L  991.4756 990.4684 990.5498 -0.0814 114 - 121 1 46 R.KLAEQFQK.Q  991.4756 990.4684 990.5498 -0.0814 114 - 121 1 --- R.KLAEQFQK.Q  1287.5930 1286.5857 1286.6870 -0.1013 62 - 72 0 79 K.DDVVIQFLNPK.V  1287.5930 1286.5857 1286.6870 -0.1013 62 - 72 0 --- K.DDVVIQFLNPK.V  1726.8523 1725.8450 1725.9664 -0.1214 46 - 61 0 66 R.IGVNAIPAIEEVNIFK.D  1726.8523 1725.8450 1725.9664 -0.1214 46 - 61 0 --- R.IGVNAIPAIEEVNIFK.D  1943.8943 1942.8870 1943.0112 -0.1242 73 - 91 0 37 K.VQASIAANTWVVSGSPQTK.K  1943.8943 1942.8870 1943.0112 -0.1242 73 - 91 0 --- K.VQASIAANTWVVSGSPQTK.K  2331.1318 2330.1245 2330.2594 -0.1348 93 - 113 0 172 K.LQDILPGIINQLGPDNLDNLR.K  2331.1318 2330.1245 2330.2594 -0.1348 93 - 113 0 --- K.LQDILPGIINQLGPDNLDNLR.K  2459.1987 2458.1914 2458.3543 -0.1629 93 - 114 1 100 K.LQDILPGIINQLGPDNLDNLRK.L  2459.1987 2458.1914 2458.3543 -0.1629 93 - 114 1 --- K.LQDILPGIINQLGPDNLDNLRK.L  2995.4922 2994.4849 2994.6429 -0.1580 46 - 72 1 115 R.IGVNAIPAIEEVNIFKDDVVIQFLNPK.V  2995.4922 2994.4849 2994.6429 -0.1580 46 - 72 1 --- R.IGVNAIPAIEEVNIFKDDVVIQFLNPK.V  No match to: 711.3436, 713.3571, 744.3445, 768.4654, 842.4390, 870.4695, 876.9780, 882.4896, 885.3726, 901.3443, 1012.3897, 1013.4352, 1045.4789, 1309.5730, 1325.5515, 1343.6616, 1475.6315, 1657.6523, 1679.7693, 1690.7058, 1748.8167, 1764.7972, 1909.8503, 1927.9033, 1927.9033, 1931.9011, 1949.8613, 1953.8595, 1959.8888, 1959.8888, 1965.8414, 1969.8297, 1974.9382, 1981.8408, 1993.8188, 1997.8281, 2143.9683, 2210.9778, 2224.9832, 2230.0608, 2233.0059, 2239.0068, 2247.0388, 2269.0110, 2272.0598, 2283.0564, 2285.0994, 2287.1384, 2296.0845, 2300.0742, 2313.1340, 2313.1340, 2314.1270, 2327.1055, 2329.1348, 2330.1531, 2338.0828, 2343.1050, 2345.1069, 2347.0906, 2351.0476, 2353.1003, 2358.0691, 2369.0469, 2369.0469, 2375.0574, 2391.0186, 2429.0234, 2435.0586, 2440.1055, 2441.1594, 2442.1846, 2456.2251, 2473.1743, 2475.1599, 2481.1829, 2497.1738, 2499.1819, 2503.1313, 2540.2661, 2612.2832, 2662.2065, 2677.1089, 2691.1064, 2720.0991, 2807.1169, 2811.1113, 2825.1140, 2914.3269, 3017.4478, 3033.4084, 3292.3508, 3337.5024, 3348.4319, 3353.4922 |

**Table S4 | Master list of proteins identified in MALDI TOF/TOF MS from B-toxic *Citrus grandis* roots using** 2DE and DIGE experiments

| **G 32 gi|2274917 Mass: 12777 Score: 179 Expect: 2.3e-012 Queries matched: 7 Cu/Zn superoxide dismutase, partial [Citrus sinensis]**  Observed Mr(expt) Mr(calc) Delta Start End Miss Ions Peptide  1076.5397 1075.5324 1075.5444 -0.0119 3 - 13 0 --- K.AVAVMTGTNGR.K  1314.7227 1313.7154 1313.7303 -0.0148 89 - 101 0 85 K.TIPLSGTNSVIGR.G  1314.7227 1313.7154 1313.7303 -0.0148 89 - 101 0 --- K.TIPLSGTNSVIGR.G  1552.7808 1551.7735 1551.7780 -0.0045 15 - 30 0 51 K.GTVSFSVEGSGPTTVK.G  1552.7808 1551.7735 1551.7780 -0.0045 15 - 30 0 --- K.GTVSFSVEGSGPTTVK.G  1680.8738 1679.8665 1679.8729 -0.0064 14 - 30 1 --- R.KGTVSFSVEGSGPTTVK.G  2468.2327 2467.2254 2467.2019 0.0236 102 - 126 0 --- R.GLVVHSSPSPDPGDGVAWGTIGLSY.-  No match to: 713.4004, 768.5177, 842.4903, 850.5190, 856.5060, 861.0480, 870.5216, 877.0212, 878.4550, 882.5473, 884.4473, 895.4355, 930.4625, 968.4766, 1003.5745, 1034.5560, 1045.5447, 1051.6931, 1060.5592, 1064.5475, 1074.5405, 1121.6813, 1165.7283, 1179.5825, 1205.5361, 1254.6696, 1270.6798, 1271.6904, 1271.6904, 1296.7094, 1328.7113, 1328.7113, 1350.5944, 1400.7693, 1414.6016, 1414.6016, 1523.9019, 1537.7058, 1606.7797, 1647.8217, 1693.8816, 1755.7935, 1876.9199, 1876.9199, 1904.8907, 1938.8822, 1993.9559, 2211.1201, 2225.1245, 2225.1245, 2239.1462, 2246.2031, 2299.1909, 2339.1406, 2339.1406, 2472.2285, 2472.2285, 2500.2268, 2500.2268, 2643.3474, 2643.3474, 2724.2642, 2807.3652, 2823.3264, 2933.5603, 2933.5603, 3461.7979 |
| --- |
| **G 26 gi|255554865 Mass: 31527 Score: 219 Expect: 2.3e-016 Queries matched: 12 lactoylglutathione lyase, putative [Ricinus communis]**  Observed Mr(expt) Mr(calc) Delta Start End Miss Ions Peptide  871.4592 870.4519 870.5075 -0.0556 23 - 29 0 --- R.LLHAVYR.V  1030.5033 1029.4960 1029.5567 -0.0606 160 - 168 1 --- R.VGDLDRSIR.F  1278.5676 1277.5603 1277.6292 -0.0689 264 - 274 0 46 K.ITSFLDPDGWK.T  1278.5676 1277.5603 1277.6292 -0.0689 264 - 274 0 --- K.ITSFLDPDGWK.T  1440.6838 1439.6765 1439.7265 -0.0499 147 - 159 0 --- R.GPTPEPLCQVMLR.V  1465.6807 1464.6734 1464.7249 -0.0514 135 - 146 0 79 K.DPDGYIFELIQR.G  1465.6807 1464.6734 1464.7249 -0.0514 135 - 146 0 --- K.DPDGYIFELIQR.G  1507.6565 1506.6492 1506.7363 -0.0870 39 - 50 1 --- K.FYTECFGMKLLR.K  1543.7476 1542.7403 1542.8253 -0.0849 235 - 249 0 50 K.SAEVVNLVTQELGGK.I  1543.7476 1542.7403 1542.8253 -0.0849 235 - 249 0 --- K.SAEVVNLVTQELGGK.I  1714.7579 1713.7506 1713.8209 -0.0703 219 - 234 0 --- K.GNAYAQVAISTDDVYK.S  1957.9346 1956.9273 1956.9615 -0.0341 1 - 18 1 --- -.MAAEATAPNADVLEWPKK.D + Oxidation (M)  No match to: 713.3626, 761.3491, 777.3397, 842.4528, 905.4380, 924.3929, 940.3900, 1003.3891, 1111.5579, 1115.5548, 1134.4264, 1138.5582, 1139.6167, 1146.5239, 1182.4299, 1198.4224, 1198.4224, 1206.6206, 1253.5851, 1256.5198, 1264.5529, 1274.6105, 1282.5667, 1292.6007, 1294.5863, 1310.5596, 1342.5674, 1349.3199, 1349.3199, 1350.6211, 1404.6520, 1406.6661, 1421.6770, 1445.6500, 1447.6660, 1448.6981, 1449.6118, 1449.6118, 1453.6338, 1480.6654, 1481.6641, 1487.6525, 1496.6736, 1497.6792, 1497.6792, 1503.6420, 1511.6680, 1513.6760, 1513.6760, 1519.6563, 1524.6964, 1529.6573, 1535.6511, 1538.7006, 1540.6978, 1554.7040, 1565.7021, 1593.7037, 1609.6658, 1625.6716, 1634.7852, 1634.7852, 1638.7719, 1646.7250, 1648.7672, 1692.8125, 1747.8079, 1758.7572, 1781.7423, 1788.8335, 1839.8452, 1839.8452, 1843.8452, 1861.8177, 1871.8270, 1877.7937, 1953.9946, 1953.9946, 1967.9296, 1970.9698, 1972.9260, 1984.9442, 2011.9219, 2133.9656, 2211.0183, 2225.0308, 2230.1067, 2239.0515, 2342.0161, 2342.0161, 2477.1592, 2497.2036, 2691.1631, 2748.1848, 2807.1938, 2814.1973, 2819.2471, 3303.5520, 3337.6006, 3349.4592 |
| **G 15 gi|162459589 Mass: 41640 Score: 110 Expect: 1.9e-005 Queries matched: 11 12-oxo-phytodienoic acid reductase2 [Zea mays]**  Observed Mr(expt) Mr(calc) Delta Start End Miss Ions Peptide  868.4907 867.4834 867.5542 -0.0707 27 - 34 0 --- R.VVLAPLTR.C  1155.5529 1154.5456 1154.6448 -0.0991 326 - 335 0 64 K.LFLANPDLPR.R  1155.5529 1154.5456 1154.6448 -0.0991 326 - 335 0 --- K.LFLANPDLPR.R  1239.4779 1238.4706 1238.5527 -0.0821 198 - 208 0 --- R.TDQYGGSLENR.C  1541.7170 1540.7097 1540.7278 -0.0181 260 - 272 0 --- R.HSGLLYCHMVEPR.I  1596.6311 1595.6238 1595.7480 -0.1242 291 - 306 0 --- K.AFHGTFIAAGGGGYDR.E  1797.8687 1796.8614 1797.0400 -0.1785 2 - 17 1 --- M.VQQAAKEVIPLLTPYK.M  1928.9203 1927.9130 1928.0804 -0.1674 1 - 17 1 --- -.MVQQAAKEVIPLLTPYK.M  1972.8658 1971.8585 1971.9385 -0.0800 113 - 131 0 --- R.VSTNELQPNGDAPISSTDK.Q  2746.2034 2745.1961 2745.3220 -0.1258 167 - 191 0 --- R.NAVEAGFDAVEIHGAHGYLLEQFMK.D  2762.2312 2761.2239 2761.3169 -0.0930 167 - 191 0 --- R.NAVEAGFDAVEIHGAHGYLLEQFMK.D + Oxidation (M)  No match to: 823.3277, 842.4399, 852.4939, 881.4173, 883.4197, 892.4174, 895.4161, 915.4263, 919.3922, 939.3903, 952.4496, 1013.4669, 1013.4669, 1035.4294, 1055.4248, 1062.5277, 1137.4989, 1186.5117, 1235.5679, 1235.5679, 1326.5356, 1342.5377, 1368.5994, 1368.5994, 1377.6586, 1385.6263, 1385.6263, 1405.6847, 1421.6982, 1433.6340, 1446.6283, 1446.6283, 1453.6609, 1474.6505, 1489.6227, 1489.6227, 1524.6794, 1534.6392, 1537.6316, 1537.6316, 1549.7826, 1553.6365, 1553.6365, 1556.6764, 1564.6493, 1564.6493, 1568.6451, 1569.6459, 1580.6404, 1584.6031, 1594.6489, 1621.6779, 1671.7017, 1675.7458, 1698.7030, 1745.6445, 1819.8691, 1825.7484, 1832.8198, 1848.8230, 1848.8230, 1868.7108, 1885.7659, 1904.8026, 1919.8405, 1984.9736, 2049.8833, 2052.8687, 2055.8623, 2065.8889, 2106.9111, 2216.9641, 2230.0740, 2234.9590, 2280.9028, 2314.0430, 2353.0945, 2491.0347, 2555.2021, 2691.1450, 2698.1912, 2823.2563, 2840.2793, 2840.2793, 2854.2671, 3295.4927 |
| **G 49 gi|162459589 Mass: 41640 Score: 98 Expect: 0.00029 Queries matched: 11 12-oxo-phytodienoic acid reductase2 [Zea mays]**  Observed Mr(expt) Mr(calc) Delta Start End Miss Ions Peptide  868.5035 867.4963 867.5542 -0.0579 27 - 34 0 --- R.VVLAPLTR.C  1155.5586 1154.5513 1154.6448 -0.0934 326 - 335 0 49 K.LFLANPDLPR.R  1155.5586 1154.5513 1154.6448 -0.0934 326 - 335 0 --- K.LFLANPDLPR.R  1239.4921 1238.4848 1238.5527 -0.0679 198 - 208 0 --- R.TDQYGGSLENR.C  1541.7235 1540.7162 1540.7278 -0.0116 260 - 272 0 --- R.HSGLLYCHMVEPR.I  1596.6487 1595.6414 1595.7480 -0.1066 291 - 306 0 --- K.AFHGTFIAAGGGGYDR.E  1797.8942 1796.8869 1797.0400 -0.1530 2 - 17 1 --- M.VQQAAKEVIPLLTPYK.M  1928.9296 1927.9223 1928.0804 -0.1581 1 - 17 1 --- -.MVQQAAKEVIPLLTPYK.M  1972.8855 1971.8782 1971.9385 -0.0603 113 - 131 0 --- R.VSTNELQPNGDAPISSTDK.Q  2746.2146 2745.2073 2745.3220 -0.1146 167 - 191 0 --- R.NAVEAGFDAVEIHGAHGYLLEQFMK.D  2762.2263 2761.2190 2761.3169 -0.0979 167 - 191 0 --- R.NAVEAGFDAVEIHGAHGYLLEQFMK.D + Oxidation (M)  No match to: 823.3410, 832.2518, 842.4514, 852.5063, 881.4298, 892.4307, 895.4322, 915.4409, 919.4053, 925.3597, 939.4020, 952.4657, 1013.4806, 1013.4806, 1035.4470, 1055.4318, 1062.5385, 1137.5188, 1186.5282, 1218.5564, 1235.5839, 1235.5839, 1257.5601, 1273.5358, 1326.5533, 1342.5557, 1368.6124, 1368.6124, 1377.6737, 1385.6405, 1385.6405, 1405.6913, 1421.7065, 1433.6436, 1446.6436, 1446.6436, 1489.6400, 1489.6400, 1537.6465, 1537.6465, 1549.7998, 1553.6506, 1553.6506, 1556.6917, 1564.6609, 1564.6609, 1568.6605, 1580.6566, 1584.6174, 1594.6648, 1671.7159, 1698.7263, 1819.8848, 1825.7749, 1832.8286, 1848.8373, 1848.8373, 1868.7382, 1870.7715, 1885.7894, 1904.8195, 1919.8507, 1984.9980, 2049.8950, 2052.8823, 2106.9204, 2211.0100, 2234.9697, 2239.0200, 2280.9175, 2314.0552, 2353.1084, 2555.2183, 2718.2710, 2823.2605, 2840.2925, 2840.2925, 2993.3142 |
| **G 12 gi|153793260 Mass: 35718 Score: 284 Expect: 7.4e-023 Queries matched: 9 chilling-responsive protein [Nicotiana tabacum]**  Observed Mr(expt) Mr(calc) Delta Start End Miss Ions Peptide  1310.5463 1309.5390 1309.6125 -0.0734 261 - 271 0 76 K.DFGSALWDMIR.G  1310.5463 1309.5390 1309.6125 -0.0734 261 - 271 0 --- K.DFGSALWDMIR.G  1326.5449 1325.5376 1325.6074 -0.0698 261 - 271 0 (36) K.DFGSALWDMIR.G + Oxidation (M)  1326.5449 1325.5376 1325.6074 -0.0698 261 - 271 0 --- K.DFGSALWDMIR.G + Oxidation (M)  1328.6770 1327.6697 1327.7499 -0.0802 155 - 166 0 --- K.VDLIVDVPVFGR.L  1569.8553 1568.8480 1568.9289 -0.0809 153 - 166 1 110 R.IKVDLIVDVPVFGR.L  1569.8553 1568.8480 1568.9289 -0.0809 153 - 166 1 --- R.IKVDLIVDVPVFGR.L  1733.8811 1732.8738 1732.9512 -0.0773 246 - 260 0 84 K.NGISYIDIPITFRPK.D  1733.8811 1732.8738 1732.9512 -0.0773 246 - 260 0 --- K.NGISYIDIPITFRPK.D  No match to: 713.3655, 768.4779, 842.4470, 856.4512, 858.4627, 870.4787, 874.3766, 876.9813, 882.5034, 920.4031, 971.5431, 1001.4274, 1005.4216, 1033.4172, 1070.5144, 1098.6077, 1258.5519, 1262.2424, 1262.2424, 1262.5313, 1266.5234, 1289.6643, 1294.1724, 1294.5200, 1300.6185, 1314.5479, 1330.5414, 1342.5338, 1358.5289, 1358.5289, 1374.5206, 1387.5903, 1403.5857, 1417.5631, 1480.7373, 1490.6316, 1494.7501, 1494.7501, 1508.7285, 1509.7255, 1533.7577, 1538.6616, 1550.6503, 1554.6661, 1596.7357, 1675.8162, 1689.8278, 1701.8164, 1716.8593, 1716.8593, 1735.8802, 1739.8483, 1753.8174, 1755.8358, 1772.8220, 1779.8075, 1795.8102, 1796.8455, 1840.8878, 1874.8994, 1890.9128, 1943.0360, 2077.9646, 2091.0608, 2160.0483, 2223.1284, 2299.0554, 2299.0554, 2331.1282, 2332.1299, 2338.9946, 2349.1482, 2349.1482, 2477.2373, 2477.2373, 2537.0957, 2616.1526, 2616.1526, 2623.1384, 2639.1682, 2691.1677, 2695.1606, 2723.1599, 2807.1785, 3131.3628, 3147.4072, 3337.6145, 3349.4929, 3353.5798 |
| **G 25 gi|334261583 Mass: 49415 Score: 297 Expect: 3.7e-024 Queries matched: 12 alpha-tubulin [Pellia endiviifolia (species B)]**  Observed Mr(expt) Mr(calc) Delta Start End Miss Ions Peptide  1007.3594 1006.3521 1006.4468 -0.0947 97 - 105 0 --- K.EDAANNFAR.G  1396.6376 1395.6303 1395.7510 -0.1207 85 - 96 0 75 R.QLFHPEQLISGK.E  1396.6376 1395.6303 1395.6856 -0.0553 391 - 401 1 --- R.IDHKFDLMYAK.R + Oxidation (M)  1683.7338 1682.7265 1682.8239 -0.0973 339 - 352 1 --- K.RTVQFVDWCPTGFK.C  1691.7084 1690.7011 1690.8638 -0.1627 216 - 229 0 56 R.SLDIERPTYTNLNR.L  1691.7084 1690.7011 1690.8638 -0.1627 216 - 229 0 --- R.SLDIERPTYTNLNR.L  1808.7648 1807.7575 1807.9178 -0.1603 265 - 280 0 --- R.IHFMLSSYAPVISAEK.A + Oxidation (M)  2329.7869 2328.7796 2329.0109 -0.2312 403 - 422 0 122 R.AFVHWYVGEGMEEGEFSEAR.E  2329.7869 2328.7796 2329.0109 -0.2312 403 - 422 0 --- R.AFVHWYVGEGMEEGEFSEAR.E  2345.7883 2344.7810 2345.0058 -0.2248 403 - 422 0 (47) R.AFVHWYVGEGMEEGEFSEAR.E + Oxidation (M)  2345.7883 2344.7810 2345.0058 -0.2248 403 - 422 0 --- R.AFVHWYVGEGMEEGEFSEAR.E + Oxidation (M)  2695.0122 2694.0049 2694.2669 -0.2619 281 - 304 0 --- K.AYHEQLSVPEITNAVFEPSSMMAK.C + Oxidation (M)  No match to: 712.1845, 832.2333, 842.4242, 1132.4550, 1219.3698, 1267.3793, 1283.3854, 1299.3788, 1378.6213, 1379.6200, 1407.5753, 1418.5850, 1434.5739, 1531.7516, 1531.7516, 1538.6055, 1549.5358, 1549.5358, 1584.5996, 1649.6501, 1657.7235, 1673.7156, 1674.6876, 1687.7211, 1701.7355, 1701.7355, 1715.7313, 1820.6560, 1820.6560, 1827.6044, 1868.7126, 1868.7126, 1875.6045, 1884.7087, 1884.7087, 1891.6061, 1913.7478, 2127.7935, 2281.7854, 2281.7854, 2286.7900, 2313.7874, 2349.7991, 2350.8040, 2361.7878, 2367.9138, 2377.7681, 2384.9448, 2408.9502, 2408.9502, 2424.9895, 2559.0088, 3209.2825 |
| **G 34 gi|255115691 Mass: 41639 Score: 169 Expect: 2.3e-011 Queries matched: 12 actin 1 [Boehmeria nivea]**  Observed Mr(expt) Mr(calc) Delta Start End Miss Ions Peptide  767.4126 766.4053 766.4337 -0.0284 331 - 337 0 --- K.VVAPPER.K  1176.5035 1175.4962 1175.5281 -0.0319 199 - 208 0 --- R.GYMFTTTAER.E  1192.4965 1191.4892 1191.5230 -0.0338 199 - 208 0 --- R.GYMFTTTAER.E + Oxidation (M)  1445.6814 1444.6741 1444.6582 0.0159 362 - 374 0 36 K.GEYDESGPSIVHR.K  1445.6814 1444.6741 1444.6582 0.0159 362 - 374 0 --- K.GEYDESGPSIVHR.K  1515.7130 1514.7057 1514.7419 -0.0361 87 - 97 0 --- K.IWHHTFYNELR.V  1547.6947 1546.6874 1546.8025 -0.1150 180 - 193 1 --- R.LDLAGRDLTDSLMK.I  1788.8829 1787.8756 1787.9053 -0.0297 241 - 256 0 98 K.NYELPDGQIITIGAER.F  1788.8829 1787.8756 1787.8876 -0.0119 194 - 208 1 --- K.ILTERGYMFTTTAER.E  1954.0420 1953.0347 1953.0571 -0.0223 98 - 115 0 --- R.VAPEEHPVLLTEAPLNPK.A  2183.0334 2182.0261 2182.0728 -0.0466 294 - 314 0 --- K.DLYGNIVLSGGSTMFPGIADR.M  2199.0564 2198.0491 2198.0677 -0.0186 294 - 314 0 --- K.DLYGNIVLSGGSTMFPGIADR.M + Oxidation (M)  No match to: 703.3138, 749.3701, 789.3673, 832.2783, 842.4768, 870.5123, 891.4426, 913.4548, 939.3494, 944.3556, 998.4624, 1023.4451, 1034.5028, 1036.4954, 1042.4907, 1049.4918, 1059.5258, 1064.5364, 1065.5127, 1068.5392, 1081.4900, 1100.5535, 1104.6228, 1108.4946, 1112.5618, 1120.6140, 1128.5111, 1132.4995, 1139.6523, 1146.5532, 1158.6879, 1238.5094, 1429.7067, 1444.7554, 1455.6803, 1457.7203, 1457.7203, 1477.6809, 1506.6599, 1507.6515, 1519.7354, 1519.7354, 1531.6998, 1581.7631, 1683.8286, 1705.7916, 1721.7734, 1738.7487, 1747.8507, 1747.8507, 1794.7911, 1805.8867, 1805.8867, 1811.8229, 1837.8671, 1853.8978, 1853.8978, 1865.8804, 1869.9078, 1876.8989, 1876.8989, 1880.8390, 1885.8782, 1901.8945, 2070.9031, 2070.9031, 2095.9705, 2102.8977, 2118.9153, 2118.9153, 2134.9490, 2134.9490, 2139.9390, 2142.0327, 2150.9121, 2155.0701, 2159.0781, 2166.9192, 2187.0562, 2187.0562, 2203.0559, 2235.0452, 2251.0627, 2267.0588, 2272.0764, 2400.1738, 2442.3198, 2691.2461, 2698.3276, 2807.2808, 2823.3184, 2941.3484, 3337.6824, 3348.6277 |
| **G 52 gi|195623832 Mass: 40879 Score: 422 Expect: 1.2e-036 Queries matched: 20 alpha-1,4-glucan-protein synthase 1 [Zea mays]**  Observed Mr(expt) Mr(calc) Delta Start End Miss Ions Peptide  839.3937 838.3865 838.4258 -0.0394 309 - 315 0 --- K.CYLELAK.Q  842.4653 841.4581 841.3970 0.0611 324 - 330 0 --- K.VDGYFNK.L  989.4875 988.4802 988.5341 -0.0539 270 - 278 0 --- K.ASNPFVNLK.K  1201.5811 1200.5738 1200.6291 -0.0553 260 - 269 0 --- K.TGLPYIWHSK.A  1222.5957 1221.5884 1221.6539 -0.0655 309 - 318 1 --- K.CYLELAKQVR.A  1222.5957 1221.5884 1221.6539 -0.0655 309 - 318 1 --- K.CYLELAKQVR.A  1283.6588 1282.6515 1282.7132 -0.0617 14 - 24 0 --- K.DELDIVIPTIR.N  1297.6484 1296.6411 1296.6570 -0.0158 72 - 83 1 --- R.MLGPKASCISFK.D + Oxidation (M)  1375.5587 1374.5514 1374.5373 0.0141 238 - 248 0 --- R.YDDMWAGWCTK.V  1501.6367 1500.6294 1500.6884 -0.0590 55 - 66 0 99 R.VPEGFDYELYNR.N  1501.6367 1500.6294 1500.6884 -0.0590 55 - 66 0 --- R.VPEGFDYELYNR.N  2121.9812 2120.9739 2121.0386 -0.0647 218 - 237 0 120 R.ELIGPAMYFGLMGDGQPIGR.Y  2121.9812 2120.9739 2121.0386 -0.0647 218 - 237 0 --- R.ELIGPAMYFGLMGDGQPIGR.Y  2137.9922 2136.9849 2137.0335 -0.0486 218 - 237 0 (70) R.ELIGPAMYFGLMGDGQPIGR.Y + Oxidation (M)  2137.9922 2136.9849 2137.0335 -0.0486 218 - 237 0 --- R.ELIGPAMYFGLMGDGQPIGR.Y + Oxidation (M)  2153.9912 2152.9839 2153.0285 -0.0445 218 - 237 0 --- R.ELIGPAMYFGLMGDGQPIGR.Y + 2 Oxidation (M)  2292.0713 2291.0640 2291.1262 -0.0622 127 - 145 0 119 K.NLLSPSTPFFFNTLYDPYR.V  2292.0713 2291.0640 2291.1262 -0.0622 127 - 145 0 --- K.NLLSPSTPFFFNTLYDPYR.V  3315.6697 3314.6624 3314.7298 -0.0674 160 - 189 0 --- R.EGVPTAVSHGLWLNIPDYDAPTQLVKPLER.N  3321.6555 3320.6482 3320.6328 0.0154 25 - 51 0 --- R.NLDFLEMWRPFFQPYHLIIVQDGDPSK.T + Oxidation (M)  No match to: 720.3451, 775.3486, 799.3667, 825.3469, 832.2654, 854.3669, 893.5447, 913.4153, 928.4399, 943.3830, 986.4523, 1020.4136, 1068.4176, 1069.5779, 1175.5441, 1186.5410, 1204.5819, 1205.5806, 1210.6044, 1220.5493, 1236.5463, 1251.6837, 1252.5381, 1335.5771, 1370.4929, 1405.5690, 1405.5690, 1434.4916, 1464.6671, 1468.6594, 1483.6451, 1522.8214, 1524.8281, 1557.6996, 1570.6304, 1582.7312, 1608.6780, 1618.6556, 1624.7491, 1666.7130, 1666.7130, 1682.7198, 1698.7328, 1714.7289, 1730.7267, 1730.7267, 1733.7333, 1744.7823, 1746.7311, 1873.9338, 1919.8888, 1973.8850, 1977.8866, 2002.1262, 2002.1262, 2005.8857, 2025.9784, 2025.9784, 2041.9490, 2073.9778, 2073.9778, 2089.9941, 2089.9941, 2142.0256, 2158.2078, 2275.0625, 2395.1079, 2412.1687, 2438.2261, 2442.2209, 2443.2231, 2470.2148, 2769.4856, 2787.5320, 2824.2710, 2863.3308, 3276.5825, 3308.6191, 3320.6606, 3331.6472, 3347.6272 |
| **G 46 gi|222356608 Mass: 40264 Score: 323 Expect: 9.3e-027 Queries matched: 13 ATPase alpha subunit, partial (mitochondrion) [Afrothismia hydra]**  Observed Mr(expt) Mr(calc) Delta Start End Miss Ions Peptide  775.3719 774.3646 774.3984 -0.0338 324 - 331 0 --- R.VGSAAQSR.A  842.4412 841.4339 841.4657 -0.0318 371 - 378 1 --- R.GARLTEVP.-  892.4164 891.4091 891.4814 -0.0723 342 - 348 0 --- K.LELAQYR.E  1026.5154 1025.5081 1025.5869 -0.0788 92 - 101 0 --- K.AVDSLVPIGR.G  1210.5897 1209.5824 1209.6717 -0.0893 47 - 58 0 --- R.VVDALGVPIDGR.G  1438.7483 1437.7410 1437.8415 -0.1005 310 - 323 0 25 R.GLRPAINVGLSVSR.V  1438.7483 1437.7410 1437.8415 -0.1005 310 - 323 0 --- R.GLRPAINVGLSVSR.V  1505.7269 1504.7196 1504.7595 -0.0399 211 - 223 0 --- K.SMHALIIFDDLSK.Q + Oxidation (M)  1537.6366 1536.6293 1536.7361 -0.1067 242 - 254 0 80 R.EAFPGDVFYLHSR.L  1537.6366 1536.6293 1536.7361 -0.1067 242 - 254 0 --- R.EAFPGDVFYLHSR.L  2178.0222 2177.0149 2177.1302 -0.1153 211 - 229 1 --- K.SMHALIIFDDLSKQAVAYR.Q  2308.0034 2306.9961 2307.1494 -0.1533 349 - 370 0 173 R.EVAAFAQFGSDLDAATQALLNR.G  2308.0034 2306.9961 2307.1494 -0.1533 349 - 370 0 --- R.EVAAFAQFGSDLDAATQALLNR.G  No match to: 748.3052, 755.3823, 766.2862, 782.2878, 796.3005, 803.3671, 804.3384, 812.2935, 847.3458, 870.4717, 906.4535, 916.4196, 931.4585, 932.4235, 994.4214, 994.4214, 1010.4904, 1028.4119, 1032.4896, 1046.4121, 1050.4071, 1091.5037, 1099.5046, 1113.3872, 1118.4709, 1122.4941, 1129.3827, 1162.5269, 1174.5017, 1177.4207, 1178.4861, 1185.5846, 1190.4974, 1193.3839, 1203.5739, 1206.5081, 1284.6636, 1336.5754, 1336.5754, 1340.5736, 1355.6932, 1368.6539, 1372.6625, 1384.5574, 1433.6416, 1460.4435, 1478.6560, 1557.6301, 1562.7374, 1565.5763, 1565.5763, 1607.5045, 1619.7153, 1707.7083, 1720.7472, 1721.7504, 1738.7705, 1738.7705, 1790.7837, 1791.6389, 1792.6879, 1796.7437, 1813.7695, 1813.7695, 1825.7592, 1825.7592, 1847.6844, 1854.7632, 1869.7588, 1887.7673, 1887.7673, 1938.6655, 1938.6655, 2031.8943, 2123.8525, 2156.0801, 2210.9873, 2233.0154, 2239.0034, 2295.1292, 2295.1292, 2333.0908, 2446.0923, 2464.0593, 2516.0261, 2535.1116, 2551.1243, 2574.1240, 2691.1028, 2695.0977, 2703.1892, 2807.1489, 2839.1958, 2930.4780, 2955.2100, 3337.5496, 3342.5959, 3390.5649, 3406.6003 |
| **G 24 gi|82400168 Mass: 37549 Score: 113 Expect: 9.3e-006 Queries matched: 6 adenosine kinase isoform 1T-like protein [Solanum tuberosum]**  Observed Mr(expt) Mr(calc) Delta Start End Miss Ions Peptide  1739.7450 1738.7377 1738.8526 -0.1148 229 - 243 0 --- K.VHGWETDNVEEIALK.I  1811.8468 1810.8395 1810.9676 -0.1281 257 - 274 0 --- R.ITVITQGADPVVVAEDGK.V  1875.7236 1874.7163 1874.8508 -0.1345 209 - 224 0 96 K.ALPYMDFVFGNETEAR.I + Oxidation (M)  1875.7236 1874.7163 1874.8508 -0.1345 209 - 224 0 --- K.ALPYMDFVFGNETEAR.I + Oxidation (M)  1967.9506 1966.9433 1967.0687 -0.1254 256 - 274 1 --- K.RITVITQGADPVVVAEDGK.V  2159.9641 2158.9568 2159.0139 -0.0571 318 - 337 1 --- R.AGCYASNVIIQRSGCTYPEK.T  No match to: 871.3336, 1039.5964, 1185.4626, 1238.5870, 1239.5721, 1254.5739, 1255.5549, 1256.5983, 1256.5983, 1260.5959, 1272.5927, 1273.5200, 1284.5848, 1288.5824, 1303.5377, 1319.5332, 1321.5641, 1321.5641, 1351.5735, 1351.5735, 1378.6208, 1386.5692, 1396.5198, 1410.6008, 1465.7380, 1470.7198, 1500.6095, 1755.7389, 1784.6969, 1799.8177, 1809.7500, 1825.7295, 1827.7212, 1827.7212, 1841.7919, 1843.7238, 1857.7430, 1860.7161, 1873.7294, 1874.7296, 1886.8263, 1889.7638, 1890.7799, 1891.7289, 1891.7289, 1907.7432, 1910.7905, 1911.8409, 1912.7555, 1912.7555, 1917.7548, 1918.7401, 1919.7410, 1932.7429, 1934.7532, 1959.8285, 1960.7876, 1960.7876, 1975.8689, 1976.7964, 1976.7964, 1992.7854, 2002.8217, 2006.7924, 2008.7987, 2017.7982, 2024.8025, 2024.8025, 2040.8031, 2040.8031, 2056.7979, 2081.8047, 2208.9951, 2211.9075, 2228.0068, 2228.8477, 2262.8193, 2275.8130, 2301.8389, 2317.8755, 2319.8291, 2319.8291, 2321.3826, 2333.8506, 2335.8579, 2360.8538, 2379.0129, 2544.0127, 2678.1030, 2691.0713, 2800.3206, 2807.1038, 3129.4336, 3132.3352, 3176.4253, 3202.4487, 3203.3872, 3218.3921, 3220.4099, 3233.3884, 3242.3223, 3260.4116, 3277.3679, 3322.4392, 3347.4326, 3477.5444 |
| **G 22 gi|295687231 Mass: 33099 Score: 316 Expect: 4.6e-026 Queries matched: 12 triosephosphate isomerase [Gossypium hirsutum]**  Observed Mr(expt) Mr(calc) Delta Start End Miss Ions Peptide  954.4411 953.4338 953.4759 -0.0421 58 - 65 0 42 K.FFVGGNWK.C  954.4411 953.4338 953.4759 -0.0421 58 - 65 0 --- K.FFVGGNWK.C  1096.5138 1095.5065 1095.5825 -0.0760 142 - 150 0 --- K.WVILGHSER.R  1331.6465 1330.6392 1330.6881 -0.0488 111 - 122 0 --- R.IEVSAQNSWIGK.G  1435.6957 1434.6884 1434.7354 -0.0470 123 - 136 0 63 K.GGAFTGEISVEQLK.D  1435.6957 1434.6884 1434.7354 -0.0470 123 - 136 0 --- K.GGAFTGEISVEQLK.D  1449.7107 1448.7034 1448.7511 -0.0476 290 - 303 0 76 K.GPEFATIVNSVTSK.K  1449.7107 1448.7034 1448.7511 -0.0476 290 - 303 0 --- K.GPEFATIVNSVTSK.K  1457.6672 1456.6599 1456.6921 -0.0322 58 - 70 1 --- K.FFVGGNWKCNGTK.D  1548.7468 1547.7395 1547.7831 -0.0435 275 - 289 0 93 K.QEDIDGFLVGGASLK.G  1548.7468 1547.7395 1547.7831 -0.0435 275 - 289 0 --- K.QEDIDGFLVGGASLK.G  2748.2405 2747.2332 2747.4129 -0.1797 111 - 136 1 --- R.IEVSAQNSWIGKGGAFTGEISVEQLK.D  No match to: 705.3179, 713.3806, 741.3547, 768.4828, 842.4657, 856.4801, 870.4976, 882.5225, 914.4800, 951.4589, 952.4583, 958.4341, 970.4336, 972.4628, 986.4269, 1045.5154, 1065.4991, 1069.5009, 1082.5314, 1082.5314, 1086.5261, 1094.5264, 1097.4922, 1098.5120, 1104.4943, 1114.5135, 1134.4961, 1139.5444, 1285.5773, 1285.5773, 1291.6969, 1307.5649, 1315.6521, 1319.6493, 1347.6387, 1357.6276, 1430.7223, 1471.6963, 1485.7153, 1513.7222, 1513.7222, 1530.7341, 1531.7242, 1570.7327, 1575.7488, 1601.7832, 1602.7853, 1619.7991, 1619.7991, 1633.7970, 1639.8318, 1641.8000, 1645.7882, 1645.7882, 1649.7927, 1657.7588, 1660.7223, 1671.7645, 1676.8054, 1679.8225, 1688.7715, 1703.8340, 1717.8457, 1718.8641, 1737.8328, 1752.8130, 1755.8163, 1774.7950, 1778.9056, 1847.8763, 1847.8763, 1858.8688, 1992.0020, 2001.9663, 2005.9736, 2042.9882, 2042.9882, 2178.0479, 2211.0566, 2216.1511, 2225.0676, 2246.1450, 2257.0906, 2299.1321, 2330.1123, 2348.1213, 2348.1213, 2556.1255, 2691.2021, 2707.1934, 2806.4006, 2809.2927, 3438.6045, 3470.6023 |
| **G 42 gi|76573375 Mass: 27694 Score: 215 Expect: 5.9e-016 Queries matched: 8 triosphosphate isomerase-like protein [Solanum tuberosum]**  Observed Mr(expt) Mr(calc) Delta Start End Miss Ions Peptide  954.4235 953.4162 953.4759 -0.0596 5 - 12 0 44 K.FFVGGNWK.C  954.4235 953.4162 953.4759 -0.0596 5 - 12 0 --- K.FFVGGNWK.C  1049.5316 1048.5243 1048.5916 -0.0673 114 - 123 0 --- K.VAYALSQGLK.V  1695.8031 1694.7958 1694.8740 -0.0781 176 - 190 0 99 K.VATPAQAQEVHFELR.K  1695.8031 1694.7958 1694.8740 -0.0781 176 - 190 0 --- K.VATPAQAQEVHFELR.K  1823.8983 1822.8910 1822.9689 -0.0779 176 - 191 1 48 K.VATPAQAQEVHFELRK.W  1823.8983 1822.8910 1822.9689 -0.0779 176 - 191 1 --- K.VATPAQAQEVHFELRK.W  2842.4670 2841.4597 2841.5163 -0.0566 220 - 246 0 --- K.ELAAQPDVDGFLVGGASLKPEFIDIIK.S  No match to: 713.3663, 763.3583, 768.4792, 832.2500, 842.4490, 857.4039, 866.3888, 870.4804, 882.5061, 900.4065, 925.4347, 931.4077, 941.4752, 958.4173, 960.4736, 970.4163, 976.4047, 986.4091, 992.3805, 1016.4702, 1070.5052, 1130.5499, 1141.5275, 1149.5339, 1151.6069, 1212.5272, 1223.5398, 1256.5630, 1269.5958, 1349.6075, 1352.5990, 1374.6320, 1374.6320, 1383.6366, 1397.6130, 1404.6920, 1413.6058, 1415.6659, 1482.7133, 1503.7389, 1503.7389, 1513.6619, 1525.7108, 1555.8352, 1567.7875, 1595.7742, 1596.7672, 1613.7941, 1613.7941, 1617.7974, 1628.7633, 1629.7772, 1635.7739, 1639.7732, 1645.7754, 1651.7976, 1659.8312, 1659.8312, 1678.7885, 1717.7819, 1729.7231, 1752.8124, 1796.8187, 1858.8495, 1862.8126, 1862.8126, 1897.8621, 1944.8900, 1954.8743, 1956.9097, 1956.9097, 1960.9102, 1961.9041, 1972.8953, 1972.8953, 1988.8979, 1988.8979, 2010.9244, 2013.9102, 2167.0798, 2253.1272, 2325.0237, 2552.2686, 2570.2893, 2570.2893, 2691.1787, 2807.2224, 2864.4185, 2977.4590, 3009.4292, 3025.4070, 3029.3982, 3041.4541, 3057.4077 |
| **G 51 gi|332198142 Mass: 42105 Score: 120 Expect: 1.9e-006 Queries matched: 9 phosphoglycerate kinase [Arabidopsis thaliana]**  Observed Mr(expt) Mr(calc) Delta Start End Miss Ions Peptide  1072.6061 1071.5988 1071.6440 -0.0452 75 - 83 0 --- K.YSLKPLVPR.L  1465.7736 1464.7663 1464.8162 -0.0499 166 - 178 0 --- K.FLKPSVAGFLMQK.E  1481.7725 1480.7652 1480.8111 -0.0459 166 - 178 0 53 K.FLKPSVAGFLMQK.E + Oxidation (M)  1481.7725 1480.7652 1480.8111 -0.0459 166 - 178 0 --- K.FLKPSVAGFLMQK.E + Oxidation (M)  1539.8529 1538.8456 1538.8919 -0.0463 262 - 276 0 --- K.GVSLLLPTDVVIADK.F  1753.8192 1752.8119 1752.8545 -0.0425 317 - 331 0 --- K.TIIWNGPMGVFEFDK.F  1769.8152 1768.8079 1768.8494 -0.0415 317 - 331 0 47 K.TIIWNGPMGVFEFDK.F + Oxidation (M)  1769.8152 1768.8079 1768.8494 -0.0415 317 - 331 0 --- K.TIIWNGPMGVFEFDK.F + Oxidation (M)  2863.3379 2862.3306 2862.3785 -0.0479 307 - 331 1 --- K.TFSEALDTTKTIIWNGPMGVFEFDK.F + Oxidation (M)  No match to: 712.2173, 713.3788, 734.4605, 745.3498, 804.2388, 825.4180, 832.2709, 842.4677, 850.4909, 855.0094, 861.0249, 870.4983, 877.0019, 882.5224, 911.4550, 928.4548, 981.5413, 1051.6162, 1056.5016, 1069.5936, 1069.5936, 1086.5922, 1121.6501, 1156.5869, 1186.5504, 1222.6061, 1283.6719, 1360.6841, 1404.6897, 1417.7681, 1417.7681, 1471.6526, 1500.6047, 1501.6381, 1562.7555, 1626.7322, 1705.8079, 1705.8079, 1709.8032, 1744.7648, 1773.8132, 1801.8069, 1802.8317, 1873.9484, 1889.8954, 1901.9009, 1901.9009, 1902.8976, 1919.9121, 1919.9121, 1976.9110, 1993.9113, 2003.9625, 2023.9381, 2041.9537, 2041.9537, 2174.1069, 2174.1069, 2222.1047, 2238.1086, 2238.1086, 2292.0718, 2292.0718, 2353.1641, 2353.1641, 2395.1218, 2660.2939, 2691.2188, 2695.2212, 2807.2349, 2824.2769, 3315.6885, 3346.6584 |
| **G 50 gi|226509380 Mass: 48719 Score: 92 Expect: 0.0012 Queries matched: 12 dihydrolipoyllysine-residue succinyltransferase component of 2-oxoglutarate dehydrogenase complex [Zea mays]**  Observed Mr(expt) Mr(calc) Delta Start End Miss Ions Peptide  852.4857 851.4784 851.5592 -0.0808 319 - 326 0 --- K.GLVVPVIR.D  868.4754 867.4681 867.5112 -0.0431 220 - 226 1 --- R.VPMPRLR.K  881.4067 880.3994 880.4806 -0.0812 424 - 430 0 --- R.EAVFFLR.R  1013.4489 1012.4416 1012.5085 -0.0669 273 - 281 0 --- K.LGLMSCFVK.A + Oxidation (M)  1070.4869 1069.4796 1069.5767 -0.0971 432 - 440 1 --- R.IKDVVEDPR.R  1155.5352 1154.5279 1154.6118 -0.0838 193 - 203 0 --- K.MQAPKPTAPSK.T  1155.5352 1154.5279 1154.6118 -0.0838 193 - 203 0 --- K.MQAPKPTAPSK.T  1180.5173 1179.5100 1179.6135 -0.1035 204 - 214 0 --- K.TSPSEPQLPPK.E  1385.6025 1384.5952 1384.5816 0.0136 327 - 338 0 --- R.DADTMNFADIEK.G + Oxidation (M)  1385.6025 1384.5952 1384.5816 0.0136 327 - 338 0 --- R.DADTMNFADIEK.G + Oxidation (M)  2555.1584 2554.1511 2554.3390 -0.1879 282 - 305 0 60 K.AAVSALQNQPIVNAVIDGDDIIYR.D  2555.1584 2554.1511 2554.3390 -0.1879 282 - 305 0 --- K.AAVSALQNQPIVNAVIDGDDIIYR.D  No match to: 706.3292, 743.3116, 759.3508, 832.2300, 833.3606, 842.4271, 863.3948, 870.4610, 892.4019, 919.3773, 921.3729, 939.3800, 946.4587, 988.4292, 993.3676, 1046.4598, 1072.4856, 1076.5227, 1090.4923, 1094.4426, 1109.4884, 1124.5160, 1140.5197, 1186.4941, 1235.5459, 1235.5459, 1239.4618, 1270.6187, 1292.6440, 1301.5889, 1315.5889, 1326.5237, 1344.5741, 1348.5436, 1368.5839, 1373.5645, 1378.5505, 1393.6332, 1393.6332, 1405.6749, 1405.6749, 1421.6654, 1424.6980, 1425.6926, 1429.5515, 1429.5515, 1446.6018, 1446.6018, 1462.5957, 1467.7185, 1474.6432, 1474.6432, 1478.6163, 1485.5356, 1489.6028, 1510.5955, 1523.6400, 1526.5988, 1537.6035, 1542.6310, 1549.7565, 1553.6171, 1556.7106, 1564.6187, 1564.6187, 1576.7061, 1579.6393, 1580.6274, 1659.7795, 1668.7375, 1671.6752, 1675.7316, 1675.7316, 1723.8217, 1735.8044, 1783.7317, 1848.7843, 1852.7778, 1868.7052, 1961.8563, 2117.8135, 2149.8928, 2151.9346, 2216.9482, 2261.9348, 2291.9641, 2301.9673, 2301.9673, 2497.1545, 2546.2432, 2840.2134, 3212.1885, 3229.2280 |
| **G 27 gi|295291644 Mass: 63583 Score: 286 Expect: 4.6e-023 Queries matched: 16 ketol-acid reductoisomerase [Catharanthus roseus]**  Observed Mr(expt) Mr(calc) Delta Start End Miss Ions Peptide  823.3240 822.3168 822.3871 -0.0704 305 - 311 0 --- K.SDIFGER.G  1046.4471 1045.4398 1045.5266 -0.0868 417 - 426 0 --- K.EGLPAFPMGK.I  1062.4431 1061.4358 1061.5215 -0.0857 417 - 426 0 --- K.EGLPAFPMGK.I + Oxidation (M)  1399.6487 1398.6414 1398.6561 -0.0147 49 - 63 0 --- R.AASFTTSCGSAVAAR.M  1680.8557 1679.8484 1679.9722 -0.1238 312 - 327 0 98 R.GILLGAVHGIVESLFR.R  1680.8557 1679.8484 1679.9722 -0.1238 312 - 327 0 --- R.GILLGAVHGIVESLFR.R  1695.7249 1694.7176 1694.8548 -0.1372 359 - 373 1 --- K.GMLAVYNALTEEEKK.E  1695.7249 1694.7176 1694.8548 -0.1372 359 - 373 1 --- K.GMLAVYNALTEEEKK.E  1711.7229 1710.7156 1710.8498 -0.1341 359 - 373 1 --- K.GMLAVYNALTEEEKK.E + Oxidation (M)  1711.7229 1710.7156 1710.8498 -0.1341 359 - 373 1 --- K.GMLAVYNALTEEEKK.E + Oxidation (M)  1735.7372 1734.7299 1734.8822 -0.1522 1 - 19 0 --- -.MAAAAATSISVSTAPAATK.T + Oxidation (M)  1836.9391 1835.9318 1836.0733 -0.1415 312 - 328 1 --- R.GILLGAVHGIVESLFRR.Y  2085.8611 2084.8538 2084.9875 -0.1336 258 - 277 0 159 K.EINGAGINSSFAVHQDVDGR.A  2085.8611 2084.8538 2084.9875 -0.1336 258 - 277 0 --- K.EINGAGINSSFAVHQDVDGR.A  2931.2461 2930.2388 2930.3868 -0.1480 474 - 499 0 --- K.GHSYSEIINESVIESVDSLNPFMHAR.G  2947.2346 2946.2273 2946.3817 -0.1544 474 - 499 0 --- K.GHSYSEIINESVIESVDSLNPFMHAR.G + Oxidation (M)  No match to: 842.4349, 899.3835, 960.3886, 998.4464, 1198.5635, 1358.5580, 1365.6042, 1390.5872, 1445.5618, 1464.6536, 1483.5695, 1496.5760, 1496.5760, 1515.6313, 1531.6063, 1543.6960, 1544.6093, 1544.6093, 1545.5939, 1547.5879, 1560.5691, 1560.5691, 1576.6190, 1601.6896, 1602.6926, 1619.7266, 1619.7266, 1626.7620, 1638.7358, 1639.6667, 1647.7103, 1663.7211, 1671.7813, 1677.6886, 1678.6962, 1687.6705, 1693.7286, 1694.7594, 1703.6658, 1708.8044, 1718.7379, 1731.7227, 1737.8134, 1747.7588, 1763.6649, 1788.7838, 1819.8201, 1827.8041, 1953.9252, 2067.8506, 2067.8506, 2068.8464, 2075.8826, 2116.9131, 2122.9651, 2139.9514, 2147.8240, 2185.9746, 2187.9651, 2203.9717, 2210.9636, 2238.9851, 2374.1118, 2392.1482, 2454.9800, 2483.1638, 2557.1045, 2591.1636, 2689.1235, 2692.1086, 2720.1042, 2737.1606, 2748.1155, 2753.1509, 2807.1216, 2883.2026, 2883.2026, 2920.3193, 2929.2659, 2929.2659, 2932.2368, 2933.2446, 2961.2678, 2961.2678, 2984.3887, 3011.2988, 3034.3777, 3039.3672, 3050.3467, 3066.3447, 3074.2739, 3162.1687, 3210.1538, 3226.2092, 3527.5762, 3555.5620 |
| **G 28 gi|295291644 Mass: 63583 Score: 332 Expect: 1.2e-027 Queries matched: 16 ketol-acid reductoisomerase [Catharanthus roseus]**  Observed Mr(expt) Mr(calc) Delta Start End Miss Ions Peptide  701.3785 700.3712 700.4231 -0.0519 405 - 411 0 --- R.SVVLAGR.R  703.3072 702.3000 702.3483 -0.0483 244 - 250 0 --- K.GMGPSVR.R  719.3013 718.2941 718.3432 -0.0491 244 - 250 0 --- K.GMGPSVR.R + Oxidation (M)  742.3420 741.3348 741.3809 -0.0462 412 - 416 1 --- R.RFYEK.E  823.3392 822.3320 822.3871 -0.0552 305 - 311 0 --- K.SDIFGER.G  1046.4714 1045.4641 1045.5266 -0.0625 417 - 426 0 --- K.EGLPAFPMGK.I  1062.4642 1061.4569 1061.5215 -0.0646 417 - 426 0 --- K.EGLPAFPMGK.I + Oxidation (M)  1680.8867 1679.8794 1679.9722 -0.0928 312 - 327 0 75 R.GILLGAVHGIVESLFR.R  1680.8867 1679.8794 1679.9722 -0.0928 312 - 327 0 --- R.GILLGAVHGIVESLFR.R  2085.9126 2084.9053 2084.9875 -0.0821 258 - 277 0 159 K.EINGAGINSSFAVHQDVDGR.A  2085.9126 2084.9053 2084.9875 -0.0821 258 - 277 0 --- K.EINGAGINSSFAVHQDVDGR.A  2931.2935 2930.2862 2930.3868 -0.1006 474 - 499 0 62 K.GHSYSEIINESVIESVDSLNPFMHAR.G  2931.2935 2930.2862 2930.3868 -0.1006 474 - 499 0 --- K.GHSYSEIINESVIESVDSLNPFMHAR.G  2947.3286 2946.3213 2946.3817 -0.0604 474 - 499 0 (1) K.GHSYSEIINESVIESVDSLNPFMHAR.G + Oxidation (M)  2947.3286 2946.3213 2946.3817 -0.0604 474 - 499 0 --- K.GHSYSEIINESVIESVDSLNPFMHAR.G + Oxidation (M)  3075.4292 3074.4219 3074.4767 -0.0547 473 - 499 1 --- K.KGHSYSEIINESVIESVDSLNPFMHAR.G + Oxidation (M)  No match to: 713.3685, 734.4430, 738.4180, 768.4840, 778.3572, 802.3926, 842.4531, 870.4824, 882.5076, 899.4042, 920.4556, 954.4517, 960.4083, 998.4644, 1045.4896, 1100.5459, 1120.5133, 1198.5695, 1223.5651, 1226.5487, 1344.6294, 1347.6232, 1390.6125, 1464.6787, 1496.5978, 1496.5978, 1504.6807, 1543.7321, 1544.6086, 1544.6086, 1548.6161, 1560.6072, 1560.6072, 1577.7051, 1601.7552, 1601.7552, 1602.7462, 1619.7654, 1619.7654, 1641.7523, 1660.6812, 1660.6812, 1671.8126, 1676.6855, 1704.7839, 1747.8379, 1869.7954, 2059.9368, 2067.9060, 2067.9060, 2068.9001, 2107.9028, 2123.0110, 2147.8601, 2188.0210, 2204.0186, 2211.0210, 2264.0640, 2392.2119, 2737.2180, 2883.3525, 2883.3525, 2886.2551, 3034.4626, 3038.4634, 3039.4634, 3050.4507, 3060.3865, 3066.4478, 3162.3438, 3165.2305, 3210.2544, 3226.3103 |
| **G 9 gi|332193639 Mass: 30175 Score: 483 Expect: 9.3e-043 Queries matched: 22 14-3-3-like protein GF14 phi [Arabidopsis thaliana]**  Observed Mr(expt) Mr(calc) Delta Start End Miss Ions Peptide  770.3700 769.3627 769.4010 -0.0383 140 - 145 0 --- R.YLAEFK.T  816.3790 815.3718 815.4137 -0.0419 20 - 26 0 --- K.LAEQAER.Y  907.4764 906.4691 906.5174 -0.0483 52 - 59 0 --- R.NLLSVAYK.N  917.4813 916.4740 916.5229 -0.0489 71 - 78 0 --- R.IISSIEQK.E  922.3738 921.3666 921.4127 -0.0461 133 - 139 1 --- K.MKGDYHR.Y + Oxidation (M)  1045.5165 1044.5092 1044.5022 0.0070 1 - 11 0 --- -.MAAPPASSSAR.E  1127.4950 1126.4877 1126.5367 -0.0490 83 - 92 0 14 R.GNDDHVTTIR.D  1127.4950 1126.4877 1126.5367 -0.0490 83 - 92 0 --- R.GNDDHVTTIR.D  1189.6145 1188.6072 1188.6536 -0.0464 225 - 234 0 66 K.DSTLIMQLLR.D  1189.6145 1188.6072 1188.6536 -0.0464 225 - 234 0 --- K.DSTLIMQLLR.D  1205.6094 1204.6021 1204.6485 -0.0464 225 - 234 0 (42) K.DSTLIMQLLR.D + Oxidation (M)  1205.6094 1204.6021 1204.6485 -0.0464 225 - 234 0 --- K.DSTLIMQLLR.D + Oxidation (M)  1334.5154 1333.5081 1333.5570 -0.0488 27 - 36 0 --- R.YEEMVEFMEK.V  1350.5095 1349.5022 1349.5519 -0.0497 27 - 36 0 --- R.YEEMVEFMEK.V + Oxidation (M)  1366.5054 1365.4981 1365.5468 -0.0487 27 - 36 0 --- R.YEEMVEFMEK.V + 2 Oxidation (M)  1418.6675 1417.6602 1417.7412 -0.0810 71 - 82 1 --- R.IISSIEQKEESR.G  1517.8073 1516.8000 1516.8725 -0.0725 52 - 65 1 --- R.NLLSVAYKNVIGAR.R  1787.8853 1786.8780 1786.9326 -0.0545 163 - 179 0 120 K.AAQDIANAELAPTHPIR.L  1787.8853 1786.8780 1786.9326 -0.0545 163 - 179 0 --- K.AAQDIANAELAPTHPIR.L  2128.9778 2127.9705 2127.9847 -0.0142 206 - 224 0 --- K.QAFDEAIAELDTLGEESYK.D  2331.1331 2330.1258 2330.1946 -0.0688 180 - 199 0 190 R.LGLALNFSVFYYEILNSPDR.A  2331.1331 2330.1258 2330.1946 -0.0688 180 - 199 0 --- R.LGLALNFSVFYYEILNSPDR.A  No match to: 712.2636, 713.3805, 728.4047, 742.4097, 832.2645, 838.5057, 842.4651, 870.4961, 935.3954, 937.4052, 948.3770, 1010.4814, 1081.5175, 1081.5175, 1141.2677, 1141.2677, 1141.6050, 1157.6008, 1187.5946, 1197.6125, 1202.5769, 1221.5946, 1254.5946, 1254.5946, 1265.5577, 1302.5046, 1323.5526, 1324.5758, 1498.7727, 1564.7235, 1573.8114, 1577.7593, 1732.7756, 1743.8616, 1748.9128, 1750.7903, 1750.7903, 1769.8776, 1770.8779, 1772.8588, 1792.8966, 1801.8816, 1802.8990, 1809.8486, 1825.8160, 1844.8787, 1871.9097, 1885.9313, 1918.8993, 1928.8594, 1957.9785, 2051.1396, 2110.9751, 2111.9478, 2113.9797, 2118.9185, 2122.9600, 2125.0012, 2125.0012, 2150.9153, 2166.9104, 2182.9116, 2186.9292, 2201.9822, 2211.0498, 2214.9104, 2315.1045, 2315.1045, 2379.1138, 2440.0139, 2440.0139, 2445.0122, 2658.1934, 2691.2083, 2807.2300, 3350.5405, 3354.6777 |
| **G 10 gi|332193639 Mass: 30175 Score: 436 Expect: 4.6e-038 Queries matched: 21 14-3-3-like protein GF14 phi [Arabidopsis thaliana]**  Observed Mr(expt) Mr(calc) Delta Start End Miss Ions Peptide  770.3621 769.3548 769.4010 -0.0462 140 - 145 0 --- R.YLAEFK.T  816.3709 815.3636 815.4137 -0.0501 20 - 26 0 --- K.LAEQAER.Y  906.3708 905.3635 905.4177 -0.0542 133 - 139 1 --- K.MKGDYHR.Y  917.4701 916.4628 916.5229 -0.0601 71 - 78 0 --- R.IISSIEQK.E  922.3674 921.3601 921.4127 -0.0526 133 - 139 1 --- K.MKGDYHR.Y + Oxidation (M)  1127.4841 1126.4768 1126.5367 -0.0599 83 - 92 0 8 R.GNDDHVTTIR.D  1127.4841 1126.4768 1126.5367 -0.0599 83 - 92 0 --- R.GNDDHVTTIR.D  1189.5964 1188.5891 1188.6536 -0.0645 225 - 234 0 72 K.DSTLIMQLLR.D  1189.5964 1188.5891 1188.6536 -0.0645 225 - 234 0 --- K.DSTLIMQLLR.D  1205.5920 1204.5847 1204.6485 -0.0638 225 - 234 0 (37) K.DSTLIMQLLR.D + Oxidation (M)  1205.5920 1204.5847 1204.6485 -0.0638 225 - 234 0 --- K.DSTLIMQLLR.D + Oxidation (M)  1334.5021 1333.4948 1333.5570 -0.0621 27 - 36 0 --- R.YEEMVEFMEK.V  1350.4962 1349.4889 1349.5519 -0.0630 27 - 36 0 --- R.YEEMVEFMEK.V + Oxidation (M)  1366.4994 1365.4921 1365.5468 -0.0547 27 - 36 0 --- R.YEEMVEFMEK.V + 2 Oxidation (M)  1517.7915 1516.7842 1516.8725 -0.0883 52 - 65 1 --- R.NLLSVAYKNVIGAR.R  1787.8531 1786.8458 1786.9326 -0.0867 163 - 179 0 119 K.AAQDIANAELAPTHPIR.L  1787.8531 1786.8458 1786.9326 -0.0867 163 - 179 0 --- K.AAQDIANAELAPTHPIR.L  1893.9181 1892.9108 1892.9632 -0.0523 2 - 19 1 --- M.AAPPASSSAREEFVYLAK.L  2128.9478 2127.9405 2127.9847 -0.0442 206 - 224 0 --- K.QAFDEAIAELDTLGEESYK.D  2331.0959 2330.0886 2330.1946 -0.1060 180 - 199 0 155 R.LGLALNFSVFYYEILNSPDR.A  2331.0959 2330.0886 2330.1946 -0.1060 180 - 199 0 --- R.LGLALNFSVFYYEILNSPDR.A  No match to: 713.3678, 742.4007, 838.4940, 842.4576, 870.4900, 876.9878, 882.5094, 907.4310, 1010.4677, 1081.5038, 1081.5038, 1108.4673, 1108.4673, 1118.5859, 1141.2345, 1141.2345, 1141.5929, 1163.5265, 1179.5344, 1197.5968, 1202.5654, 1211.5786, 1227.5630, 1254.5800, 1254.5800, 1294.5878, 1324.5751, 1358.5441, 1367.4988, 1548.6289, 1577.7440, 1582.6080, 1661.7369, 1679.8267, 1732.7515, 1743.8375, 1750.7681, 1750.7681, 1769.8629, 1770.8551, 1792.8755, 1802.8696, 1803.8563, 1809.8456, 1820.7822, 1825.8097, 1844.8427, 1871.8815, 1957.9557, 1993.8743, 2111.9238, 2118.8840, 2124.9722, 2124.9722, 2150.8931, 2166.8872, 2182.8906, 2211.0273, 2214.8853, 2237.9636, 2237.9636, 2308.9199, 2324.9109, 2379.0828, 2439.9937, 2658.1609, 2691.1606, 2807.1924, 3166.4131 |
| **G 6 gi|255584432 Mass: 25994 Score: 191 Expect: 1.5e-013 Queries matched: 11 proteasome subunit alpha type, putative [Ricinus communis]**  Observed Mr(expt) Mr(calc) Delta Start End Miss Ions Peptide  825.4193 824.4120 824.4504 -0.0384 87 - 93 0 --- R.TLVEHAR.V  883.3979 882.3906 882.4308 -0.0401 94 - 100 0 --- R.VETQNHR.F  1063.4755 1062.4682 1062.5094 -0.0411 11 - 20 0 65 R.GVNTFSPEGR.L  1063.4755 1062.4682 1062.5094 -0.0411 11 - 20 0 --- R.GVNTFSPEGR.L  1098.5499 1097.5426 1097.5829 -0.0403 208 - 217 0 --- K.VTPNNVDIAR.V  1399.7266 1398.7193 1398.7606 -0.0413 54 - 66 0 --- R.ITSPLLEPSSVEK.V  1423.7460 1422.7387 1422.7758 -0.0371 21 - 32 0 83 R.LFQVEYAIEAIK.L  1423.7460 1422.7387 1422.7758 -0.0371 21 - 32 0 --- R.LFQVEYAIEAIK.L  1940.8617 1939.8544 1939.8759 -0.0214 167 - 185 0 --- K.AIGSGSEGADSSLQEQYNK.D  2487.1206 2486.1133 2486.3553 -0.2419 186 - 207 1 --- K.DITLLEAETIALSILKQVMEEK.V  2503.1257 2502.1184 2502.3502 -0.2318 186 - 207 1 --- K.DITLLEAETIALSILKQVMEEK.V + Oxidation (M)  No match to: 709.3345, 725.3290, 842.4697, 1045.4894, 1195.4413, 1259.4436, 1439.2841, 1439.5546, 1439.5546, 1445.7206, 1461.6934, 1485.5901, 1487.5680, 1490.7379, 1503.5680, 1503.5680, 1602.7417, 1603.8080, 1649.8036, 1677.8024, 1757.9581, 1779.9310, 1795.9097, 1829.8364, 1972.8713, 1986.9602, 2078.9656, 2086.0273, 2105.9773, 2115.0452, 2127.9795, 2141.0679, 2142.0742, 2152.9001, 2153.9871, 2158.0864, 2159.0745, 2159.0745, 2170.0256, 2170.0256, 2174.0637, 2181.0591, 2186.0154, 2197.0759, 2201.9910, 2211.0820, 2216.0815, 2218.0239, 2218.0239, 2225.0828, 2228.1155, 2231.1624, 2234.0225, 2234.0225, 2239.0879, 2250.0676, 2254.0806, 2256.1326, 2272.1501, 2272.1501, 2273.6814, 2283.1257, 2288.1265, 2294.0972, 2310.0928, 2313.1128, 2329.1482, 2350.0879, 2397.1794, 2400.0881, 2414.1458, 2423.0994, 2439.1567, 2441.1265, 2441.1265, 2449.1270, 2455.1541, 2459.1406, 2489.1309, 2489.1309, 2505.1506, 2505.1506, 2521.1094, 2527.1382, 2532.1108, 2546.1384, 2662.3018, 2691.2317, 2695.2224, 2709.2205, 2720.2493, 2721.2290, 2724.2446, 2748.2520, 2764.2615, 2807.2568, 2823.2686, 2914.4712, 3290.7251, 3338.6860, 3346.6304 |
| **G 13 gi|32700048 Mass: 30683 Score: 218 Expect: 2.9e-016 Queries matched: 9 26S proteasome subunit RPN12 [Arabidopsis thaliana]**  Observed Mr(expt) Mr(calc) Delta Start End Miss Ions Peptide  702.3661 701.3588 701.4072 -0.0484 57 - 62 0 --- K.ELTIAR.D  742.4116 741.4043 741.4497 -0.0453 116 - 121 0 --- R.LLVQNR.I  1707.8126 1706.8053 1706.7933 0.0120 1 - 14 0 --- -.MDPQLTEVSQQFER.F  1845.9280 1844.9207 1844.9996 -0.0788 246 - 261 0 86 K.EIPSLQLINQTLSYAR.E  1845.9280 1844.9207 1844.9996 -0.0788 246 - 261 0 --- K.EIPSLQLINQTLSYAR.E  1880.7753 1879.7680 1879.8522 -0.0842 144 - 159 0 102 K.HAVELEQSFMEGAYNR.V  1880.7753 1879.7680 1879.8522 -0.0842 144 - 159 0 --- K.HAVELEQSFMEGAYNR.V  1896.7827 1895.7754 1895.8471 -0.0717 144 - 159 0 --- K.HAVELEQSFMEGAYNR.V + Oxidation (M)  2313.1072 2312.0999 2312.1874 -0.0875 165 - 184 0 --- R.QTAPDATYVYFMDLLAKPIR.D  No match to: 713.3680, 739.4026, 744.4288, 764.2881, 768.4821, 791.3886, 805.4381, 809.3770, 842.4525, 846.3776, 870.4842, 882.5070, 914.3673, 972.4430, 987.4310, 987.4310, 1022.4661, 1057.4686, 1062.5262, 1094.5669, 1101.5243, 1108.4252, 1108.4252, 1179.5334, 1204.5286, 1222.5376, 1222.5376, 1226.5510, 1230.4438, 1262.4318, 1273.5348, 1277.5316, 1289.5316, 1305.5272, 1350.6666, 1358.7096, 1364.5751, 1386.6895, 1458.6621, 1475.6718, 1551.7316, 1569.7483, 1569.7483, 1583.7115, 1591.7339, 1692.7551, 1692.7551, 1711.7948, 1714.7550, 1716.7637, 1737.8196, 1737.8196, 1761.8038, 1809.8330, 1825.8480, 1827.8951, 1832.7422, 1832.7422, 1848.3293, 1850.8601, 1869.8824, 1878.7845, 1879.7932, 1884.7937, 1891.9196, 1891.9196, 1907.9491, 1912.7720, 1937.9120, 1939.9712, 1939.9712, 1955.9828, 1955.9828, 1971.9528, 1993.8767, 2002.8979, 2018.8813, 2152.0071, 2211.0325, 2216.0203, 2225.0298, 2257.0452, 2383.8577, 2458.1143, 2515.2004, 2705.0410, 3346.5720 |
| **G 19 gi|289365 Mass: 57657 Score: 435 Expect: 5.9e-038 Queries matched: 26 60-kDa chaperonin-60 alpha-polypeptide precursor, partial [Brassica napus]**  Observed Mr(expt) Mr(calc) Delta Start End Miss Ions Peptide  731.3212 730.3139 730.3609 -0.0471 344 - 349 0 --- K.DELQAR.I  733.3174 732.3102 732.3554 -0.0453 282 - 288 0 --- K.APGFGER.R  844.4343 843.4271 843.4814 -0.0543 398 - 404 1 --- K.LRIEDAK.N  898.5048 897.4975 897.5647 -0.0672 273 - 281 0 --- R.GVLNVVAVK.A  940.5002 939.4929 939.5641 -0.0712 236 - 243 0 --- K.DIIPILEK.T  1043.5135 1042.5062 1042.5771 -0.0708 53 - 62 0 --- K.VVNDGVTIAR.A  1090.5240 1089.5167 1089.5818 -0.0651 215 - 223 0 56 K.LLVEFENAR.V  1090.5240 1089.5167 1089.5818 -0.0651 215 - 223 0 --- K.LLVEFENAR.V  1204.5414 1203.5341 1203.6135 -0.0794 42 - 52 0 --- R.NVVLDEFGSPK.V  1290.5354 1289.5281 1289.6099 -0.0818 385 - 396 0 --- K.VGAATETELEDR.K  1418.6335 1417.6262 1417.7048 -0.0786 385 - 397 1 --- K.VGAATETELEDRK.L  1479.6588 1478.6515 1478.7405 -0.0890 202 - 214 0 57 R.GYISPQFVTNPEK.L  1479.6588 1478.6515 1478.7405 -0.0890 202 - 214 0 --- R.GYISPQFVTNPEK.L  1501.6451 1500.6378 1500.6467 -0.0089 356 - 368 0 --- K.ELSETDSVYDSEK.L  1636.6776 1635.6703 1635.7700 -0.0997 85 - 101 0 37 K.TNDSAGDGTTTASVLAR.E  1636.6776 1635.6703 1635.7700 -0.0997 85 - 101 0 --- K.TNDSAGDGTTTASVLAR.E  1754.8113 1753.8040 1753.9032 -0.0992 63 - 79 0 64 R.AIELPDAMENAGAALIR.E  1754.8113 1753.8040 1753.9032 -0.0992 63 - 79 0 --- R.AIELPDAMENAGAALIR.E  1770.8129 1769.8056 1769.8981 -0.0925 63 - 79 0 (49) R.AIELPDAMENAGAALIR.E + Oxidation (M)  1770.8129 1769.8056 1769.8981 -0.0925 63 - 79 0 --- R.AIELPDAMENAGAALIR.E + Oxidation (M)  1904.8429 1903.8356 1903.9487 -0.1130 332 - 349 1 118 K.DSTTLIADAASKDELQAR.I  1904.8429 1903.8356 1903.9487 -0.1130 332 - 349 1 --- K.DSTTLIADAASKDELQAR.I  2291.0403 2290.0330 2290.1362 -0.1031 150 - 172 0 --- K.AVATISAGNDELVGTMIADAIDK.V + Oxidation (M)  2519.3069 2518.2996 2518.4733 -0.1737 249 - 272 1 --- R.APLLIIAEDVTGEALATLVVNKLR.G  3098.2925 3097.2852 3097.4284 -0.1432 173 - 201 0 --- K.VGPDGVLSIESSSSFETTVEVEEGMEIDR.G  3114.3186 3113.3113 3113.4233 -0.1120 173 - 201 0 --- K.VGPDGVLSIESSSSFETTVEVEEGMEIDR.G + Oxidation (M)  No match to: 713.3662, 798.4019, 842.4497, 856.4611, 864.3640, 870.4788, 882.4998, 1025.4823, 1036.4731, 1045.5062, 1059.4680, 1066.4818, 1075.4761, 1077.4672, 1112.4930, 1121.4945, 1132.5569, 1139.6132, 1146.5143, 1177.5771, 1179.5314, 1180.5635, 1198.6017, 1198.6017, 1215.5079, 1231.5013, 1232.5139, 1253.5908, 1255.6062, 1270.6125, 1274.5934, 1280.6782, 1286.5684, 1294.5803, 1334.6497, 1342.5555, 1367.6525, 1409.6843, 1409.6843, 1431.5547, 1449.6818, 1472.5895, 1475.6609, 1516.6530, 1517.6438, 1555.8022, 1564.6838, 1596.7554, 1606.7754, 1649.6698, 1700.7822, 1706.8043, 1706.8043, 1752.8058, 1776.7941, 1778.8081, 1786.7922, 1791.6649, 1834.8705, 1857.8441, 1956.8705, 1972.8798, 1993.8783, 2043.9526, 2150.0623, 2156.8918, 2156.8918, 2206.9460, 2210.9983, 2225.0017, 2227.0269, 2230.0549, 2239.0144, 2246.0786, 2273.1384, 2273.1384, 2295.0679, 2383.8235, 2705.0205, 2716.9543, 2954.4321, 2954.4321, 3050.2886 |
| **G 48 gi|257196367 Mass: 16620 Score: 438 Expect: 2.9e-038 Queries matched: 22 ubiquitin-conjugating enzyme variant [Citrus sinensis]**  Observed Mr(expt) Mr(calc) Delta Start End Miss Ions Peptide  901.4517 900.4444 900.4916 -0.0472 18 - 24 0 39 R.LLEELER.G  901.4517 900.4444 900.4916 -0.0472 18 - 24 0 --- R.LLEELER.G  941.3792 940.3719 940.4185 -0.0465 128 - 135 0 --- K.EMAAPHNR.K + Oxidation (M)  1132.5762 1131.5689 1131.6189 -0.0499 106 - 114 0 60 K.FGLLVNWQR.E  1132.5762 1131.5689 1131.6189 -0.0499 106 - 114 0 --- K.FGLLVNWQR.E  1187.5491 1186.5418 1186.5982 -0.0564 75 - 84 0 --- K.DYPEKPPSVR.F  1215.6145 1214.6072 1214.6618 -0.0546 2 - 14 0 76 M.TLGSGGSSVVVPR.N  1215.6145 1214.6072 1214.6506 -0.0434 18 - 27 1 --- R.LLEELERGEK.G  1260.6642 1259.6569 1259.7138 -0.0569 105 - 114 1 --- K.KFGLLVNWQR.E  1278.6232 1277.6159 1277.6655 -0.0496 136 - 146 1 55 R.KLVQPPEGTYF.-  1278.6232 1277.6159 1277.6655 -0.0496 136 - 146 1 --- R.KLVQPPEGTYF.-  1483.6848 1482.6775 1482.7276 -0.0500 115 - 126 0 --- R.EYTMEDILTQLK.K  1499.6798 1498.6725 1498.7225 -0.0500 115 - 126 0 --- R.EYTMEDILTQLK.K + Oxidation (M)  1611.7770 1610.7697 1610.8225 -0.0528 115 - 127 1 --- R.EYTMEDILTQLKK.E  1627.7750 1626.7677 1626.8174 -0.0497 115 - 127 1 --- R.EYTMEDILTQLKK.E + Oxidation (M)  1793.8267 1792.8194 1792.8818 -0.0623 70 - 84 1 --- K.LFCDKDYPEKPPSVR.F  1861.8748 1860.8675 1860.9231 -0.0555 48 - 64 0 60 R.SWTGTIIGPHNTVHEGR.I  1861.8748 1860.8675 1860.9231 -0.0555 48 - 64 0 --- R.SWTGTIIGPHNTVHEGR.I  2136.8286 2135.8213 2135.8775 -0.0562 28 - 47 0 --- K.GIGDGTVSYGMDDGDDIYMR.S  2152.8462 2151.8389 2151.8724 -0.0335 28 - 47 0 --- K.GIGDGTVSYGMDDGDDIYMR.S + Oxidation (M)  2168.8423 2167.8350 2167.8674 -0.0323 28 - 47 0 42 K.GIGDGTVSYGMDDGDDIYMR.S + 2 Oxidation (M)  2168.8423 2167.8350 2167.8674 -0.0323 28 - 47 0 --- K.GIGDGTVSYGMDDGDDIYMR.S + 2 Oxidation (M)  No match to: 713.3781, 768.4943, 810.3261, 815.4070, 819.4022, 831.4026, 832.2659, 842.4643, 847.3959, 856.4756, 861.0222, 870.4947, 876.9991, 880.3857, 882.5190, 1045.5067, 1072.5310, 1130.5637, 1136.5706, 1146.5564, 1148.5676, 1152.5618, 1154.5598, 1160.5630, 1162.5627, 1164.5619, 1164.5619, 1170.5500, 1172.5212, 1180.5547, 1254.6071, 1264.6575, 1273.6233, 1276.6511, 1292.6487, 1300.6023, 1316.6335, 1531.6591, 1759.8375, 1817.8589, 1827.8401, 1831.8345, 1832.8448, 1833.8536, 1843.8564, 1844.8494, 1847.8560, 1848.8573, 1850.8649, 1850.8649, 1853.2712, 1855.8738, 1859.8654, 1865.8711, 1872.8558, 1875.8486, 1877.8521, 1877.8521, 1889.8379, 1890.8511, 1891.8621, 1893.8407, 1893.8407, 1918.8649, 1922.8627, 1983.8948, 2040.8505, 2040.8505, 2056.9590, 2088.8374, 2104.8442, 2104.8442, 2113.0239, 2120.8452, 2211.0442, 2225.0566, 2246.1521, 2299.1179, 2691.1843, 2695.1968, 3346.6272 |
| **G 53 gi|332198044 Mass: 17181 Score: 387 Expect: 3.7e-033 Queries matched: 14 ubiquitin-conjugating enzyme E2 35 [Arabidopsis thaliana]**  Observed Mr(expt) Mr(calc) Delta Start End Miss Ions Peptide  970.4812 969.4739 969.5396 -0.0657 97 - 104 0 38 K.WSPALQIR.T  970.4812 969.4739 969.5396 -0.0657 97 - 104 0 --- K.WSPALQIR.T  999.4565 998.4492 998.5185 -0.0693 77 - 84 0 --- K.IYHPNIDK.L  1213.5958 1212.5885 1212.6615 -0.0730 95 - 104 1 53 K.DKWSPALQIR.T  1213.5958 1212.5885 1212.6615 -0.0730 95 - 104 1 --- K.DKWSPALQIR.T  1747.8073 1746.8000 1746.8902 -0.0902 56 - 70 0 --- K.LELFLPEEYPMAAPK.V  1763.8148 1762.8075 1762.8851 -0.0776 56 - 70 0 60 K.LELFLPEEYPMAAPK.V + Oxidation (M)  1763.8148 1762.8075 1762.8851 -0.0776 56 - 70 0 --- K.LELFLPEEYPMAAPK.V + Oxidation (M)  1970.8628 1969.8555 1969.9415 -0.0859 17 - 35 0 115 R.LLSEPAPGISASPSEDNMR.Y  1970.8628 1969.8555 1969.9415 -0.0859 17 - 35 0 --- R.LLSEPAPGISASPSEDNMR.Y  1986.8718 1985.8645 1985.9364 -0.0718 17 - 35 0 (15) R.LLSEPAPGISASPSEDNMR.Y + Oxidation (M)  1986.8718 1985.8645 1985.9364 -0.0718 17 - 35 0 --- R.LLSEPAPGISASPSEDNMR.Y + Oxidation (M)  2263.0366 2262.0293 2262.1030 -0.0737 36 - 55 0 75 R.YFNVMILGPTQSPYEGGVFK.L + Oxidation (M)  2263.0366 2262.0293 2262.1030 -0.0737 36 - 55 0 --- R.YFNVMILGPTQSPYEGGVFK.L + Oxidation (M)  No match to: 713.3632, 734.4407, 768.4769, 813.3610, 839.4087, 840.4120, 842.4458, 850.4731, 854.9859, 861.0050, 870.4739, 874.4383, 876.9790, 882.5013, 896.3961, 930.4164, 953.4507, 955.4367, 957.4437, 974.4754, 985.4416, 986.4575, 989.4416, 1002.4650, 1016.4716, 1051.6367, 1071.3854, 1165.6711, 1176.4954, 1211.5808, 1212.5779, 1217.5898, 1227.5763, 1229.5869, 1229.5869, 1235.5773, 1241.5785, 1243.5790, 1245.5800, 1245.5800, 1285.6040, 1306.6062, 1335.6232, 1393.6674, 1431.7424, 1473.6104, 1516.7659, 1699.8115, 1699.8115, 1785.7935, 1842.6987, 1878.8546, 1922.8516, 1922.8516, 1968.8519, 2002.8392, 2002.8392, 2199.0281, 2199.0281, 2211.0251, 2225.0305, 2246.0996, 2285.0298, 2299.0737, 2691.1587, 2695.1631, 2724.1599, 2752.1882, 2807.1797 |
| **G 29 gi|284927592 Mass: 11985 Score: 389 Expect: 2.3e-033 Queries matched: 11 polyubiquitin, partial [Citrus sinensis]**  Observed Mr(expt) Mr(calc) Delta Start End Miss Ions Peptide  717.3001 716.2928 716.3453 -0.0525 81 - 86 0 --- K.QLEDGR.T  1039.4482 1038.4409 1038.5094 -0.0685 66 - 74 0 51 K.EGIPPDQQR.L  1039.4482 1038.4409 1038.5094 -0.0685 66 - 74 0 --- K.EGIPPDQQR.L  1065.4883 1064.4810 1064.5502 -0.0692 87 - 95 0 --- R.TLADYNIQK.E  1067.5487 1066.5414 1066.6135 -0.0720 20 - 28 0 57 K.ESTLHLVLR.L  1067.5487 1066.5414 1066.6135 -0.0720 96 - 104 0 --- K.ESTLHLVLR.L  1523.6931 1522.6858 1522.7739 -0.0881 62 - 74 1 95 K.IQDKEGIPPDQQR.L  1523.6931 1522.6858 1522.7739 -0.0881 62 - 74 1 --- K.IQDKEGIPPDQQR.L  1763.8024 1762.7951 1762.8836 -0.0885 44 - 59 0 --- K.TITLEVESSDTIDNVK.A  2114.0457 2113.0384 2113.1531 -0.1147 11 - 28 1 96 R.TLADYNIQKESTLHLVLR.L  2114.0457 2113.0384 2113.1531 -0.1147 87 - 104 1 --- R.TLADYNIQKESTLHLVLR.L  No match to: 700.2725, 713.3658, 734.4420, 750.4695, 765.3756, 768.4781, 781.3721, 842.4504, 850.4750, 856.4662, 861.0045, 864.4488, 868.4838, 870.4822, 876.9799, 882.5019, 927.5168, 996.5278, 1021.4396, 1021.4396, 1045.4976, 1045.4976, 1049.5391, 1051.6357, 1061.4325, 1089.5208, 1126.4949, 1165.6642, 1327.6183, 1403.6714, 1460.6927, 1580.7075, 1580.7075, 2002.9833, 2211.0137, 2225.0222, 2225.0222, 2230.0840, 2233.0220, 2239.0342, 2246.0940, 2246.0940, 2249.0359, 2283.0520, 2283.0520, 2291.9316, 2299.0747, 2299.0747, 2313.0867, 2691.1428, 2748.1689, 2748.1689, 3346.5659 |
| **G 30 gi|19702 Mass: 17353 Score: 120 Expect: 1.9e-006 Queries matched: 10 eukaryotic initiation factor 5A (2) [Nicotiana plumbaginifolia]**  Observed Mr(expt) Mr(calc) Delta Start End Miss Ions Peptide  1134.6472 1133.6399 1133.5829 0.0570 19 - 28 0 54 K.TYPQQAGTIR.K  1134.6472 1133.6399 1133.6292 0.0108 42 - 52 1 --- K.VVEVSTSKTGK.H  1262.7498 1261.7425 1261.6779 0.0647 19 - 29 1 --- K.TYPQQAGTIRK.N  1370.8374 1369.8301 1369.7453 0.0848 117 - 128 0 --- R.LPTDDNLLTQIK.D  1389.7500 1388.7427 1388.7445 -0.0018 37 - 49 1 --- K.GRPCKVVEVSTSK.T  1870.1130 1869.1057 1868.9843 0.1214 113 - 128 1 --- K.DDLRLPTDDNLLTQIK.D  2075.1399 2074.1326 2074.0582 0.0744 117 - 135 1 --- R.LPTDDNLLTQIKDGFAEGK.D  2075.1399 2074.1326 2074.0582 0.0744 117 - 135 1 --- R.LPTDDNLLTQIKDGFAEGK.D  2159.2200 2158.2127 2158.0589 0.1538 70 - 88 1 --- K.KLEDIVPSSHNCDVPHVNR.T  2672.5430 2671.5357 2671.2864 0.2493 89 - 112 0 --- R.TDYQLIDISEDGFVSLLTENGNTK.D  No match to: 712.2780, 842.5418, 870.5757, 882.6033, 1074.4152, 1074.6158, 1090.3567, 1090.5769, 1100.6427, 1116.6367, 1118.6543, 1118.6543, 1140.6396, 1156.6304, 1246.7513, 1286.5988, 1322.7297, 1361.7533, 1362.7372, 1371.8501, 1379.7649, 1379.7649, 1384.7738, 1388.7654, 1401.7474, 1405.7560, 1406.7749, 1406.7749, 1417.7302, 1428.7603, 1444.7393, 1490.8331, 1715.9420, 1724.9595, 1788.9731, 1868.0408, 1871.1245, 1984.1104, 1997.1139, 2013.1387, 2015.1274, 2015.1274, 2029.1077, 2047.1031, 2047.1031, 2056.1926, 2058.1563, 2070.1521, 2088.1372, 2088.1372, 2092.1650, 2093.1929, 2104.1782, 2112.1875, 2120.2166, 2125.2161, 2140.2034, 2142.2031, 2143.2375, 2143.2375, 2156.2151, 2170.1997, 2175.2092, 2175.2092, 2181.2202, 2182.2319, 2185.2268, 2186.2375, 2193.2271, 2196.2444, 2198.2339, 2199.2192, 2200.2324, 2203.2473, 2203.2473, 2211.2766, 2215.2664, 2216.2375, 2216.2375, 2225.2869, 2230.3230, 2233.2888, 2239.2766, 2244.2661, 2246.3477, 2299.3601, 2313.3682, 2655.4854, 2659.5103, 2663.5149, 2678.5239, 2691.4834, 2694.4966, 2695.4822, 2710.4756, 2720.4622, 2723.4644, 2748.4954, 2752.4937, 2807.5027, 2839.5225, 3346.9773 |
| **G 11 gi|217038830 Mass: 17387 Score: 239 Expect: 2.3e-018 Queries matched: 13 eukaryotic translation initiation factor 5A1 [Glycine max]**  Observed Mr(expt) Mr(calc) Delta Start End Miss Ions Peptide  723.3312 722.3239 722.3235 0.0004 129 - 135 0 --- K.DGFAEGK.D  806.4236 805.4163 805.4697 -0.0534 30 - 36 0 --- K.NGYIVIK.N  1118.5474 1117.5401 1117.5880 -0.0479 19 - 28 0 57 K.TFPQQAGTIR.K  1118.5474 1117.5401 1117.5880 -0.0479 19 - 28 0 --- K.TFPQQAGTIR.K  1134.5339 1133.5266 1133.6292 -0.1025 42 - 52 1 --- K.VVEVSTSKTGK.H  1134.5339 1133.5266 1133.6292 -0.1025 42 - 52 1 --- K.VVEVSTSKTGK.H  1246.6277 1245.6204 1245.6829 -0.0625 19 - 29 1 --- K.TFPQQAGTIRK.N  1370.7000 1369.6927 1369.7453 -0.0525 117 - 128 0 73 R.LPTDENLLSQIK.D  1370.7000 1369.6927 1369.7453 -0.0525 117 - 128 0 --- R.LPTDENLLSQIK.D  1869.9199 1868.9126 1868.9843 -0.0717 113 - 128 1 47 K.DDLRLPTDENLLSQIK.D  1869.9199 1868.9126 1868.9843 -0.0717 113 - 128 1 --- K.DDLRLPTDENLLSQIK.D  2074.9250 2073.9177 2074.0582 -0.1405 117 - 135 1 --- R.LPTDENLLSQIKDGFAEGK.D  2074.9250 2073.9177 2074.0582 -0.1405 117 - 135 1 --- R.LPTDENLLSQIKDGFAEGK.D  No match to: 713.3760, 768.4877, 811.3339, 823.4216, 842.4648, 856.4753, 870.4923, 882.5137, 924.4949, 929.3763, 1045.5061, 1074.4551, 1100.5387, 1101.5342, 1103.3436, 1103.5315, 1116.5394, 1120.3619, 1140.5201, 1197.5928, 1197.5928, 1201.5873, 1213.5933, 1229.5851, 1286.4722, 1327.7125, 1365.5957, 1379.6279, 1384.6533, 1384.6533, 1388.6467, 1389.6146, 1406.6326, 1406.6326, 1428.6051, 1444.5886, 1490.6799, 1526.7402, 1529.7422, 1590.7068, 1654.7448, 1658.7366, 1670.7379, 1686.7384, 1699.7666, 1724.7820, 1803.7611, 2014.9244, 2046.8975, 2046.8975, 2057.9595, 2111.9761, 2120.0022, 2143.0137, 2143.0137, 2169.9690, 2174.9893, 2174.9893, 2186.0027, 2203.0134, 2203.0134, 2211.0378, 2225.0459, 2239.0552, 2246.1279, 2299.0901, 2617.1567, 2665.1531, 2672.2627, 2691.2048, 2695.1936, 2723.1902, 2807.2134, 3346.6255, 3353.6406 |
| **G 40 gi|33325129 Mass: 17461 Score: 121 Expect: 1.5e-006 Queries matched: 10 eukaryotic translation initiation factor 5A isoform VII [Hevea brasiliensis]**  Observed Mr(expt) Mr(calc) Delta Start End Miss Ions Peptide  723.3271 722.3199 722.3235 -0.0036 129 - 135 0 --- K.DGFAEGK.D  806.4299 805.4226 805.4697 -0.0471 30 - 36 0 --- K.NGYIVIK.N  1134.5404 1133.5331 1133.5829 -0.0498 19 - 28 0 55 K.TYPQQAGTIR.K  1134.5404 1133.5331 1133.5829 -0.0498 19 - 28 0 --- K.TYPQQAGTIR.K  1262.6294 1261.6221 1261.6779 -0.0557 19 - 29 1 --- K.TYPQQAGTIRK.N  1370.7040 1369.6967 1369.7453 -0.0485 117 - 128 0 --- R.LPTDENLLSQIK.D  1869.9224 1868.9151 1868.9843 -0.0692 113 - 128 1 --- K.DDLRLPTDENLLSQIK.D  2074.9387 2073.9314 2074.0582 -0.1268 117 - 135 1 --- R.LPTDENLLSQIKDGFAEGK.D  2074.9387 2073.9314 2074.0582 -0.1268 117 - 135 1 --- R.LPTDENLLSQIKDGFAEGK.D  2672.2869 2671.2796 2671.2864 -0.0068 89 - 112 0 --- R.TDYQLIDISEDGFVSLLTENGNTK.D  No match to: 713.3800, 721.3770, 734.4564, 750.4860, 768.4931, 823.4226, 836.4130, 842.4653, 850.4903, 868.4991, 870.4933, 882.5203, 996.5521, 1045.5126, 1051.6584, 1090.4840, 1116.5359, 1117.5413, 1118.5491, 1118.5491, 1156.5128, 1179.5461, 1197.5951, 1197.5951, 1201.5908, 1213.6017, 1229.5887, 1286.4778, 1286.4778, 1322.6045, 1362.6088, 1371.7249, 1371.7249, 1379.6334, 1379.6334, 1383.6472, 1384.6555, 1389.6292, 1401.5994, 1406.6387, 1417.5715, 1475.6962, 1490.6874, 1526.7445, 1590.7074, 1638.7468, 1654.7515, 1658.7478, 1716.7891, 1724.7794, 1867.8439, 1870.9459, 1870.9459, 2014.9340, 2014.9340, 2046.9095, 2125.0249, 2126.0190, 2143.0342, 2143.0342, 2156.0110, 2174.9915, 2174.9915, 2200.0371, 2203.0183, 2203.0183, 2211.0493, 2225.0601, 2239.0671, 2246.1292, 2299.1172, 2383.8889, 2617.1621, 2691.2236, 2717.0313 |
| **G 41 gi|217038830 Mass: 17387 Score: 236 Expect: 4.6e-018 Queries matched: 10 eukaryotic translation initiation factor 5A1 [Glycine max]**  Observed Mr(expt) Mr(calc) Delta Start End Miss Ions Peptide  723.3274 722.3201 722.3235 -0.0034 129 - 135 0 --- K.DGFAEGK.D  1118.5223 1117.5150 1117.5880 -0.0730 19 - 28 0 57 K.TFPQQAGTIR.K  1118.5223 1117.5150 1117.5880 -0.0730 19 - 28 0 --- K.TFPQQAGTIR.K  1246.6132 1245.6059 1245.6829 -0.0770 19 - 29 1 --- K.TFPQQAGTIRK.N  1370.6777 1369.6704 1369.7453 -0.0748 117 - 128 0 47 R.LPTDENLLSQIK.D  1370.6777 1369.6704 1369.7453 -0.0748 117 - 128 0 --- R.LPTDENLLSQIK.D  1869.8851 1868.8778 1868.9843 -0.1065 113 - 128 1 82 K.DDLRLPTDENLLSQIK.D  1869.8851 1868.8778 1868.9843 -0.1065 113 - 128 1 --- K.DDLRLPTDENLLSQIK.D  2074.8809 2073.8736 2074.0582 -0.1846 117 - 135 1 --- R.LPTDENLLSQIKDGFAEGK.D  2074.8809 2073.8736 2074.0582 -0.1846 117 - 135 1 --- R.LPTDENLLSQIKDGFAEGK.D  No match to: 713.3626, 768.4763, 842.4574, 870.4911, 882.4990, 1015.4751, 1045.5055, 1074.4375, 1100.5198, 1116.5161, 1134.5144, 1134.5144, 1140.5226, 1156.5106, 1179.4703, 1212.6216, 1286.4589, 1362.5751, 1379.5988, 1379.5988, 1385.6401, 1389.5836, 1401.5911, 1406.6036, 1406.6036, 1409.6143, 1428.5946, 1444.5934, 1475.6647, 1490.6515, 1724.7477, 1731.7521, 1821.7700, 1993.8599, 2014.8822, 2014.8822, 2046.8536, 2046.8536, 2103.9536, 2119.9521, 2125.9504, 2142.9712, 2142.9712, 2157.9417, 2174.9395, 2174.9395, 2202.9666, 2202.9666, 2210.9893, 2225.0022, 2230.0623, 2239.0134, 2672.1975, 2691.1592, 2695.1440, 2807.1602 |
| **G 7 gi|332645889 Mass: 26465 Score: 246 Expect: 4.6e-019 Queries matched: 6 Translation initiation factor IF6 [Arabidopsis thaliana]**  Observed Mr(expt) Mr(calc) Delta Start End Miss Ions Peptide  1254.5608 1253.5535 1253.6728 -0.1192 86 - 96 0 85 R.NSLPDQVVVQR.I  1254.5608 1253.5535 1253.6728 -0.1192 86 - 96 0 --- R.NSLPDQVVVQR.I  1361.5123 1360.5050 1360.6292 -0.1242 226 - 237 0 --- R.EAQPSSIVDEMR.K  1489.5972 1488.5899 1488.7242 -0.1343 226 - 238 1 --- R.EAQPSSIVDEMRK.S  1818.7910 1817.7837 1817.9410 -0.1573 124 - 139 0 143 K.ETEEIIADVLGVEVFR.Q  1818.7910 1817.7837 1817.9410 -0.1573 124 - 139 0 --- K.ETEEIIADVLGVEVFR.Q  No match to: 704.2899, 713.3400, 768.4524, 833.3273, 842.4187, 849.3252, 850.4141, 854.9622, 856.4314, 860.9739, 868.4537, 870.4488, 876.9522, 882.4719, 903.3940, 950.3716, 996.4965, 1045.4528, 1051.6035, 1069.4114, 1071.4706, 1126.4515, 1140.5093, 1165.6310, 1237.5353, 1276.5359, 1292.5090, 1294.6487, 1322.5822, 1324.5724, 1341.5492, 1341.5492, 1345.5182, 1358.6581, 1372.5292, 1388.5200, 1402.5442, 1425.5917, 1452.6031, 1499.6327, 1516.6030, 1573.5938, 1598.6078, 1662.6136, 1737.7418, 1737.7418, 1788.7142, 1788.7142, 1800.7786, 1836.7423, 1852.7444, 1852.7444, 1969.7772, 1969.7772, 1972.7889, 1980.7340, 1986.7986, 1986.7986, 1993.7306, 2010.7384, 2010.7384, 2068.8970, 2085.9275, 2085.9275, 2142.9131, 2210.9365, 2224.9453, 2246.0286, 2299.0005, 2313.0171, 2328.9956, 2366.8940, 2474.9780, 2536.0017, 2536.0017, 2547.9939, 2566.0034, 2566.0034, 2583.1499, 2691.0596, 2695.0513, 2839.1345, 3280.4856, 3346.4507 |
| **G 33 gi|222861722 Mass: 43186 Score: 598 Expect: 2.9e-054 Queries matched: 18 s-adenosylmethionine synthetase 1 family protein [Populus trichocarpa]**  Observed Mr(expt) Mr(calc) Delta Start End Miss Ions Peptide  765.4051 764.3978 764.4180 -0.0202 281 - 287 0 --- R.SGAYIVR.Q  842.4872 841.4800 841.5636 -0.0837 332 - 338 1 --- K.EILKIVK.E  900.5014 899.4941 899.5188 -0.0247 292 - 300 0 --- K.SIVANGLAR.R  955.5088 954.5015 954.5750 -0.0734 328 - 335 1 --- K.IPDKEILK.I  1141.5868 1140.5795 1140.6040 -0.0244 228 - 237 0 48 K.TIFHLNPSGR.F  1141.5868 1140.5795 1140.6040 -0.0244 228 - 237 0 --- K.TIFHLNPSGR.F  1453.7283 1452.7210 1452.7473 -0.0263 238 - 252 0 111 R.FVIGGPHGDAGLTGR.K  1453.7283 1452.7210 1452.7473 -0.0263 238 - 252 0 --- R.FVIGGPHGDAGLTGR.K  1963.9647 1962.9574 1962.9587 -0.0013 254 - 273 0 98 K.IIIDTYGGWGAHGGGAFSGK.D  1963.9647 1962.9574 1962.9587 -0.0013 254 - 273 0 --- K.IIIDTYGGWGAHGGGAFSGK.D  2055.9758 2054.9685 2054.9731 -0.0045 170 - 187 0 133 K.TQVTVEYYNDNGAMVPVR.V  2055.9758 2054.9685 2054.9731 -0.0045 170 - 187 0 --- K.TQVTVEYYNDNGAMVPVR.V  2068.0298 2067.0225 2066.9255 0.0971 1 - 18 0 --- -.METFLFTSESVNEGHPDK.L  2071.9841 2070.9768 2070.9680 0.0088 170 - 187 0 (13) K.TQVTVEYYNDNGAMVPVR.V + Oxidation (M)  2071.9841 2070.9768 2070.9680 0.0088 170 - 187 0 --- K.TQVTVEYYNDNGAMVPVR.V + Oxidation (M)  2417.2715 2416.2642 2416.2498 0.0144 92 - 113 0 141 K.VLVNIEQQSPDIAQGVHGHFTK.R  2417.2715 2416.2642 2416.2498 0.0144 92 - 113 0 --- K.VLVNIEQQSPDIAQGVHGHFTK.R  2649.3699 2648.3626 2648.3293 0.0333 188 - 211 0 --- R.VHTVLISTQHDETVTNDEIAADLK.E  No match to: 754.4049, 856.5362, 870.5154, 873.4735, 906.4386, 911.4058, 996.4443, 1198.6064, 1205.5472, 1228.6473, 1232.4875, 1248.4819, 1269.5314, 1276.6548, 1281.6312, 1285.5292, 1292.6537, 1294.6519, 1311.6465, 1317.5414, 1324.6129, 1331.6152, 1333.5422, 1346.7482, 1349.5348, 1373.6122, 1390.6313, 1390.6313, 1400.6522, 1406.6350, 1407.6268, 1409.6426, 1422.6378, 1435.7205, 1436.7212, 1438.6980, 1484.7010, 1501.6976, 1510.7429, 1518.7189, 1519.7183, 1530.7644, 1739.9031, 1756.9026, 1756.9026, 1772.9006, 1772.9006, 1834.8333, 1966.9658, 1967.9667, 2007.9663, 2007.9663, 2061.9624, 2109.9561, 2116.0293, 2116.0293, 2125.9585, 2132.0505, 2194.0588, 2194.0588, 2253.1182, 2272.1096, 2406.1375, 2439.2129, 2472.2244, 2536.3201, 2622.3154, 2641.2622, 2941.4968 |
| **G 43 gi|222861722 Mass: 43186 Score: 677 Expect: 3.7e-062 Queries matched: 17 s-adenosylmethionine synthetase 1 family protein [Populus trichocarpa]**  Observed Mr(expt) Mr(calc) Delta Start End Miss Ions Peptide  765.3637 764.3564 764.4180 -0.0617 281 - 287 0 --- R.SGAYIVR.Q  900.4530 899.4457 899.5188 -0.0731 292 - 300 0 --- K.SIVANGLAR.R  1141.5270 1140.5197 1140.6040 -0.0842 228 - 237 0 57 K.TIFHLNPSGR.F  1141.5270 1140.5197 1140.6040 -0.0842 228 - 237 0 --- K.TIFHLNPSGR.F  1288.6682 1287.6609 1287.7550 -0.0941 212 - 222 0 --- K.EHVIKPVIPEK.Y  1453.6598 1452.6525 1452.7473 -0.0948 238 - 252 0 128 R.FVIGGPHGDAGLTGR.K  1453.6598 1452.6525 1452.7473 -0.0948 238 - 252 0 --- R.FVIGGPHGDAGLTGR.K  1963.8799 1962.8726 1962.9587 -0.0861 254 - 273 0 --- K.IIIDTYGGWGAHGGGAFSGK.D  2053.8694 2052.8621 2052.9422 -0.0801 71 - 89 1 --- R.DTCRNIGFISDDVGLDADK.C  2055.8706 2054.8633 2054.9731 -0.1097 170 - 187 0 139 K.TQVTVEYYNDNGAMVPVR.V  2055.8706 2054.8633 2054.9731 -0.1097 170 - 187 0 --- K.TQVTVEYYNDNGAMVPVR.V  2071.8743 2070.8670 2070.9680 -0.1010 170 - 187 0 (17) K.TQVTVEYYNDNGAMVPVR.V + Oxidation (M)  2071.8743 2070.8670 2070.9680 -0.1010 170 - 187 0 --- K.TQVTVEYYNDNGAMVPVR.V + Oxidation (M)  2417.1521 2416.1448 2416.2498 -0.1050 92 - 113 0 136 K.VLVNIEQQSPDIAQGVHGHFTK.R  2417.1521 2416.1448 2416.2498 -0.1050 92 - 113 0 --- K.VLVNIEQQSPDIAQGVHGHFTK.R  2649.2383 2648.2310 2648.3293 -0.0983 188 - 211 0 152 R.VHTVLISTQHDETVTNDEIAADLK.E  2649.2383 2648.2310 2648.3023 -0.0712 364 - 386 1 --- K.TAAYGHFGRDDPDFTWEVVKPLK.W  No match to: 800.2907, 804.2103, 832.2414, 834.2432, 842.4385, 870.4659, 873.4414, 911.3575, 983.5089, 1097.4940, 1163.5046, 1205.4824, 1216.4241, 1225.5756, 1232.4181, 1251.4802, 1332.5358, 1373.5365, 1388.5640, 1390.5624, 1390.5624, 1400.5789, 1400.5789, 1401.5762, 1404.5802, 1406.5525, 1409.5702, 1422.5616, 1435.6423, 1436.6456, 1438.6243, 1475.6339, 1495.6781, 1501.6031, 1518.6356, 1519.6316, 1537.6820, 1569.5983, 1749.6600, 1834.7352, 1965.8734, 1965.8734, 1981.8658, 1985.8501, 1992.9104, 1995.8867, 2007.8571, 2007.8571, 2012.8589, 2013.8754, 2021.8744, 2029.8856, 2029.8856, 2038.8623, 2045.8824, 2061.8503, 2069.8711, 2085.8689, 2109.8242, 2121.9443, 2125.8333, 2131.8364, 2164.9961, 2169.9541, 2185.9666, 2201.9470, 2406.0259, 2439.1101, 2455.0671, 2531.0999, 2612.1953, 2671.1860, 2941.3601, 2941.3601, 3205.2600 |
| **G 44 gi|14600072 Mass: 43157 Score: 490 Expect: 1.9e-043 Queries matched: 23 S-adenosylmethionine synthetase [Brassica juncea]**  Observed Mr(expt) Mr(calc) Delta Start End Miss Ions Peptide  765.4111 764.4039 764.4180 -0.0142 281 - 287 0 --- R.SGAYIVR.Q  979.4612 978.4540 978.4671 -0.0132 365 - 373 0 39 K.TAAYGHFGR.D  979.4612 978.4540 978.4671 -0.0132 365 - 373 0 --- K.TAAYGHFGR.D  1141.5977 1140.5904 1140.6040 -0.0135 228 - 237 0 46 K.TIFHLNPSGR.F  1141.5977 1140.5904 1140.6040 -0.0135 228 - 237 0 --- K.TIFHLNPSGR.F  1453.7465 1452.7392 1452.7473 -0.0081 238 - 252 0 92 R.FVIGGPHGDAGLTGR.K  1453.7465 1452.7392 1452.7473 -0.0081 238 - 252 0 --- R.FVIGGPHGDAGLTGR.K  1688.8635 1687.8562 1687.8457 0.0105 374 - 387 0 --- R.DDPDFTWEVVKPLK.W  1963.9982 1962.9909 1962.9587 0.0322 254 - 273 0 --- K.IIIDTYGGWGAHGGGAFSGK.D  2013.9905 2012.9832 2012.9699 0.0133 339 - 355 0 --- K.ESFDFRPGMMTINLDLK.R  2030.0129 2029.0056 2028.9648 0.0408 339 - 355 0 --- K.ESFDFRPGMMTINLDLK.R + Oxidation (M)  2030.0129 2029.0056 2028.9648 0.0408 339 - 355 0 --- K.ESFDFRPGMMTINLDLK.R + Oxidation (M)  2046.0198 2045.0125 2044.9597 0.0528 339 - 355 0 --- K.ESFDFRPGMMTINLDLK.R + 2 Oxidation (M)  2055.9937 2054.9864 2054.9731 0.0134 170 - 187 0 116 K.TQVTVEYYNDNGAMVPVR.V  2055.9937 2054.9864 2054.9731 0.0134 170 - 187 0 --- K.TQVTVEYYNDNGAMVPVR.V  2072.0029 2070.9956 2070.9680 0.0276 170 - 187 0 (25) K.TQVTVEYYNDNGAMVPVR.V + Oxidation (M)  2072.0029 2070.9956 2070.9680 0.0276 170 - 187 0 --- K.TQVTVEYYNDNGAMVPVR.V + Oxidation (M)  2170.0969 2169.0896 2169.0710 0.0186 339 - 356 1 --- K.ESFDFRPGMMTINLDLKR.G  2186.1152 2185.1079 2185.0659 0.0420 339 - 356 1 --- K.ESFDFRPGMMTINLDLKR.G + Oxidation (M)  2202.1040 2201.0967 2201.0609 0.0359 339 - 356 1 --- K.ESFDFRPGMMTINLDLKR.G + 2 Oxidation (M)  2417.3013 2416.2940 2416.2498 0.0442 92 - 113 0 131 K.VLVNIEQQSPDIAQGVHGHFTK.R  2417.3013 2416.2940 2416.2498 0.0442 92 - 113 0 --- K.VLVNIEQQSPDIAQGVHGHFTK.R  2649.3672 2648.3599 2648.3023 0.0577 365 - 387 1 --- K.TAAYGHFGRDDPDFTWEVVKPLK.W  No match to: 832.2963, 870.4305, 883.4319, 900.5090, 911.4164, 1011.4760, 1047.4950, 1205.5601, 1216.5011, 1232.4995, 1331.6168, 1373.6206, 1377.7424, 1390.6448, 1390.6448, 1404.7338, 1407.6479, 1409.6525, 1422.6378, 1433.7238, 1433.7238, 1435.7302, 1436.7345, 1437.7239, 1438.7159, 1449.7205, 1465.7120, 1537.7788, 1599.8241, 1711.9399, 1738.8699, 1749.7699, 1767.8008, 1784.8231, 1838.9851, 1919.9635, 1965.9883, 1965.9883, 1972.9952, 1981.9877, 1985.9763, 1995.9858, 2001.9468, 2003.9849, 2007.0271, 2007.9690, 2007.9690, 2012.9653, 2034.9989, 2037.9760, 2038.9707, 2052.9929, 2058.4524, 2061.9707, 2069.9907, 2071.0020, 2109.9600, 2122.0703, 2125.9822, 2126.9795, 2153.0715, 2217.1040, 2439.2449, 2455.2346, 2461.1685, 2491.1775, 2622.3376, 2941.5356, 2941.5356, 3205.4553 |
| **G 36 gi|226528292 Mass: 45117 Score: 434 Expect: 7.4e-038 Queries matched: 24 spliceosome RNA helicase BAT1 [Zea mays]**  Observed Mr(expt) Mr(calc) Delta Start End Miss Ions Peptide  702.3445 701.3372 701.3708 -0.0336 352 - 357 0 --- R.GIDIER.V  878.4247 877.4175 877.4545 -0.0370 212 - 218 0 --- R.DVQEIFK.M  946.5051 945.4978 945.4879 0.0099 271 - 278 1 --- K.LSEAEKNR.K  994.4310 993.4238 993.4623 -0.0386 203 - 210 0 --- K.MLESLDMR.R  1010.4272 1009.4199 1009.4572 -0.0373 203 - 210 0 --- K.MLESLDMR.R + Oxidation (M)  1034.5214 1033.5141 1033.5556 -0.0415 211 - 218 1 --- R.RDVQEIFK.M  1056.6018 1055.5945 1055.6338 -0.0393 342 - 351 0 76 K.ILVATDLVGR.G  1056.6018 1055.5945 1055.6338 -0.0393 342 - 351 0 --- K.ILVATDLVGR.G  1114.6414 1113.6341 1113.6070 0.0272 138 - 146 1 --- R.FSKYLTEVK.V  1119.5786 1118.5713 1118.5066 0.0647 194 - 202 0 --- R.HFILDECDK.M  1166.6174 1165.6101 1165.5583 0.0518 203 - 211 1 --- K.MLESLDMRR.D + Oxidation (M)  1179.5542 1178.5469 1178.5832 -0.0363 40 - 50 0 49 K.GYVGIHSSGFR.D  1179.5542 1178.5469 1178.5832 -0.0363 40 - 50 0 --- K.GYVGIHSSGFR.D  1243.7059 1242.6986 1242.7336 -0.0349 51 - 60 0 52 R.DFLLKPELLR.A  1243.7059 1242.6986 1242.7336 -0.0349 51 - 60 0 --- R.DFLLKPELLR.A  1421.7900 1420.7827 1420.8190 -0.0363 259 - 270 0 --- K.LTLHGLVQHYIK.L  1537.6753 1536.6680 1536.7031 -0.0350 126 - 137 0 --- R.ELAYQICHEFER.F  1846.7604 1845.7531 1845.7801 -0.0269 244 - 258 0 --- K.FMQDPMEIYVDDEAK.L + Oxidation (M)  2236.0471 2235.0398 2235.0630 -0.0231 358 - 376 0 150 R.VNIVINYDMPDSADTYLHR.V  2236.0471 2235.0398 2235.0630 -0.0231 358 - 376 0 --- R.VNIVINYDMPDSADTYLHR.V  2252.0627 2251.0554 2251.0579 -0.0025 358 - 376 0 (65) R.VNIVINYDMPDSADTYLHR.V + Oxidation (M)  2252.0627 2251.0554 2251.0579 -0.0025 358 - 376 0 --- R.VNIVINYDMPDSADTYLHR.V + Oxidation (M)  2404.2554 2403.2481 2403.3062 -0.0581 40 - 60 1 --- K.GYVGIHSSGFRDFLLKPELLR.A  3036.5774 3035.5701 3035.3972 0.1729 302 - 328 1 --- R.AAELNKLLCECNFPSICIHSGMTQEER.L  No match to: 713.3890, 717.3397, 768.5029, 780.3727, 796.3655, 842.4739, 870.5036, 882.5203, 891.4501, 901.4564, 962.4373, 976.5178, 997.4683, 1083.5338, 1142.5442, 1161.5446, 1172.4958, 1176.4968, 1201.5321, 1263.5999, 1265.6552, 1332.5952, 1360.6088, 1426.6715, 1426.6715, 1453.6970, 1502.8356, 1562.6835, 1576.6914, 1594.6947, 1594.6947, 1601.7549, 1607.7764, 1616.6847, 1621.7168, 1635.7598, 1689.7924, 1691.8185, 1701.8551, 1711.8851, 1827.8898, 1868.8458, 1895.9384, 2045.0242, 2061.0439, 2188.0417, 2188.0417, 2274.0994, 2292.1350, 2292.1350, 2458.1431, 2458.1431, 2488.1184, 2506.1169, 2506.1169, 2522.1428, 2522.1428, 2888.2473, 2911.4124, 3080.5925, 3123.5400, 3578.7502 |
| **G 35 gi|283049402 Mass: 46906 Score: 735 Expect: 5.9e-068 Queries matched: 33 DEAD-box RNA helicase-like protein [Prunus persica]**  Observed Mr(expt) Mr(calc) Delta Start End Miss Ions Peptide  780.3704 779.3631 779.4000 -0.0369 169 - 174 0 --- R.VFDMLR.R  796.3621 795.3548 795.3949 -0.0401 169 - 174 0 --- R.VFDMLR.R + Oxidation (M)  891.4457 890.4384 890.4796 -0.0412 234 - 240 0 --- K.FMNKPVR.I  907.4381 906.4308 906.4745 -0.0437 234 - 240 0 --- K.FMNKPVR.I + Oxidation (M)  976.5150 975.5077 975.5501 -0.0424 377 - 385 0 46 K.GVAINFVTR.D  976.5150 975.5077 975.5501 -0.0424 377 - 385 0 --- K.GVAINFVTR.D  1104.6006 1103.5933 1103.6451 -0.0517 376 - 385 1 --- R.KGVAINFVTR.D  1114.6384 1113.6311 1113.6757 -0.0446 332 - 341 0 --- R.VLITTDLLAR.G  1119.5731 1118.5658 1118.6084 -0.0426 176 - 184 0 30 R.QSLRPDYIK.M  1119.5731 1118.5658 1118.6084 -0.0426 176 - 184 0 --- R.QSLRPDYIK.M  1173.6008 1172.5935 1172.6401 -0.0465 245 - 254 1 --- K.RDELTLEGIK.Q  1360.6064 1359.5991 1359.7180 -0.1189 119 - 129 1 --- R.ELAQQIEKVMR.A + Oxidation (M)  1360.6064 1359.5991 1359.7180 -0.1189 119 - 129 1 --- R.ELAQQIEKVMR.A + Oxidation (M)  1401.6864 1400.6791 1400.7259 -0.0468 77 - 90 0 --- K.GLDVIQQAQSGTGK.T  1502.8350 1501.8277 1501.8728 -0.0451 154 - 168 0 97 R.ILQAGVHVVVGTPGR.V  1502.8350 1501.8277 1501.8728 -0.0451 154 - 168 0 --- R.ILQAGVHVVVGTPGR.V  1555.6646 1554.6573 1554.7058 -0.0484 185 - 197 0 --- K.MFVLDEADEMLSR.G  1571.6672 1570.6599 1570.7007 -0.0408 185 - 197 0 27 K.MFVLDEADEMLSR.G + Oxidation (M)  1571.6672 1570.6599 1570.7007 -0.0408 185 - 197 0 --- K.MFVLDEADEMLSR.G + Oxidation (M)  1587.6670 1586.6597 1586.6956 -0.0359 185 - 197 0 --- K.MFVLDEADEMLSR.G + 2 Oxidation (M)  1784.7280 1783.7207 1783.7544 -0.0336 303 - 318 0 --- R.DHTVSATHGDMDQNTR.D  1800.7302 1799.7229 1799.7493 -0.0264 303 - 318 0 --- R.DHTVSATHGDMDQNTR.D + Oxidation (M)  1827.8916 1826.8843 1826.9315 -0.0471 54 - 69 0 104 R.GIYAYGFEKPSAIQQR.G  1827.8916 1826.8843 1826.9315 -0.0471 54 - 69 0 --- R.GIYAYGFEKPSAIQQR.G  2031.0598 2030.0525 2030.1020 -0.0495 150 - 168 1 --- R.EDQRILQAGVHVVVGTPGR.V  2045.0265 2044.0192 2044.0662 -0.0470 214 - 232 0 108 K.VQVGVFSATMPPEALEITR.K  2045.0265 2044.0192 2044.0662 -0.0470 214 - 232 0 --- K.VQVGVFSATMPPEALEITR.K  2059.0227 2058.0154 2057.9364 0.0791 1 - 19 1 --- -.MAGVAPEGSQFDAKQYDTK.M + Oxidation (M)  2061.0356 2060.0283 2060.0612 -0.0328 214 - 232 0 (37) K.VQVGVFSATMPPEALEITR.K + Oxidation (M)  2061.0356 2060.0283 2060.0612 -0.0328 214 - 232 0 --- K.VQVGVFSATMPPEALEITR.K + Oxidation (M)  2413.1558 2412.1485 2412.0910 0.0575 303 - 323 1 --- R.DHTVSATHGDMDQNTRDIIMR.E  2911.4492 2910.4419 2910.4875 -0.0456 342 - 366 0 191 R.GIDVQQVSLVINYDLPTQPENYLHR.I  2911.4492 2910.4419 2910.4875 -0.0456 342 - 366 0 --- R.GIDVQQVSLVINYDLPTQPENYLHR.I  No match to: 710.3613, 782.3696, 799.3920, 842.4683, 843.4510, 846.4289, 882.5252, 894.4364, 910.4259, 926.4517, 947.4572, 996.4267, 1023.5086, 1025.5187, 1026.4978, 1036.4901, 1047.4797, 1062.4700, 1102.5472, 1142.5278, 1241.6281, 1260.6207, 1304.6198, 1384.6632, 1415.6727, 1432.6753, 1446.7014, 1459.6613, 1494.7175, 1507.6582, 1523.6589, 1563.8049, 1598.7419, 1629.8153, 1633.7699, 1637.7689, 1639.8245, 1657.7976, 1665.7635, 1720.7845, 1809.8751, 1810.8745, 1830.2874, 1849.8784, 1895.9896, 1997.0142, 1997.0142, 2077.0090, 2373.1362, 2378.0491, 2456.1077, 2516.1707, 2516.1707, 2543.3062, 2648.2661, 2699.3372, 2699.3372, 2704.3323, 3032.5491, 3123.5413 |
| **G 21 gi|7024451 Mass: 16839 Score: 227 Expect: 3.7e-017 Queries matched: 10 glycine-rich RNA-binding protein [Citrus unshiu]**  Observed Mr(expt) Mr(calc) Delta Start End Miss Ions Peptide  930.3957 929.3884 929.4759 -0.0875 50 - 57 0 57 R.GFGFVTFR.D  930.3957 929.3884 929.4759 -0.0875 50 - 57 0 --- R.GFGFVTFR.D  1025.3387 1024.3314 1024.4321 -0.1007 88 - 100 0 --- R.GSGGGGGGGYGSR.G  1131.4683 1130.4610 1130.5680 -0.1069 78 - 87 0 43 R.NITVNEAQSR.G  1131.4683 1130.4610 1130.5680 -0.1069 78 - 87 0 --- R.NITVNEAQSR.G  1302.5280 1301.5207 1301.6404 -0.1196 50 - 60 1 48 R.GFGFVTFRDEK.S  1302.5280 1301.5207 1301.6404 -0.1196 50 - 60 1 --- R.GFGFVTFRDEK.S  1489.5383 1488.5310 1488.6626 -0.1316 64 - 77 0 --- R.DAIEGMNGQNLDGR.N  1505.5352 1504.5279 1504.6576 -0.1296 64 - 77 0 52 R.DAIEGMNGQNLDGR.N + Oxidation (M)  1505.5352 1504.5279 1504.6576 -0.1296 64 - 77 0 --- R.DAIEGMNGQNLDGR.N + Oxidation (M)  No match to: 794.2703, 842.4211, 870.4511, 912.3802, 913.3760, 915.3705, 922.3350, 922.3350, 928.3693, 929.3654, 936.3480, 936.3480, 944.4041, 952.3661, 968.3414, 978.3853, 1039.3541, 1096.4734, 1165.4747, 1179.4863, 1184.5023, 1197.5255, 1213.5188, 1234.5612, 1277.5880, 1284.5255, 1285.5208, 1320.4647, 1340.6090, 1365.5176, 1368.5533, 1383.5615, 1400.5515, 1434.6394, 1441.5234, 1441.5234, 1445.4995, 1475.6190, 1479.6156, 1487.5415, 1491.5483, 1493.5970, 1521.5270, 1657.6510, 1672.5634, 1707.6277, 1716.7042, 1733.6492, 1744.6875, 1758.6620, 1783.5757, 1783.5757, 1788.7147, 1797.6772, 1810.6816, 1811.6449, 1814.6466, 1814.6466, 1818.7310, 1825.7360, 1828.6592, 1828.6592, 1835.7891, 1839.7433, 1842.7030, 1850.6688, 1853.6996, 1856.7002, 1876.7676, 1890.7993, 1923.7241, 1929.7687, 1946.7396, 1950.7184, 1953.7290, 1967.7178, 1967.7178, 1993.8096, 1998.7687, 2012.7822, 2012.7822, 2025.7919, 2034.7776, 2103.8770, 2210.9348, 2224.9504, 2229.9910, 2232.9424, 2240.9712, 2245.9929, 2298.9958, 2367.0586, 2383.7673, 2420.9668, 2692.0654, 2720.0916, 2782.2004, 2825.0923, 3102.2451, 3132.2087, 3164.2402, 3346.4556 |
| **G 54 gi|192913008 Mass: 24885 Score: 86 Expect: 0.0051 Queries matched: 8 GTP-binding nuclear protein Ran-A1 [Elaeis guineensis]**  Observed Mr(expt) Mr(calc) Delta Start End Miss Ions Peptide  909.3911 908.3838 908.4617 -0.0778 103 - 109 0 --- K.NVPTWHR.D  1005.4918 1004.4845 1004.5225 -0.0380 136 - 143 1 --- K.AKMVTFHR.K + Oxidation (M)  1005.4918 1004.4845 1004.5225 -0.0380 136 - 143 1 --- K.AKMVTFHR.K + Oxidation (M)  1116.5438 1115.5365 1115.5723 -0.0358 32 - 40 1 --- K.RHLTGEFEK.K  1228.5358 1227.5285 1227.6135 -0.0850 146 - 155 0 --- K.NLQYYEISAK.S  1667.6810 1666.6737 1666.8024 -0.1287 1 - 15 0 --- -.MALPGQQAVDYPSFK.L + Oxidation (M)  1761.7743 1760.7670 1760.8885 -0.1215 156 - 169 0 57 K.SNYNFEKPFLYLAR.K  1761.7743 1760.7670 1760.8885 -0.1215 156 - 169 0 --- K.SNYNFEKPFLYLAR.K  No match to: 713.3523, 768.4675, 787.3527, 800.3543, 832.2377, 836.3894, 842.4358, 848.4125, 851.4445, 870.4642, 879.4337, 882.4894, 913.3866, 925.3874, 941.3790, 965.4094, 966.4460, 966.4460, 1007.4828, 1012.4584, 1051.4447, 1052.4178, 1052.4178, 1093.4905, 1114.5406, 1115.5530, 1128.4576, 1133.5795, 1133.5795, 1138.5824, 1190.5031, 1195.5122, 1211.5114, 1230.5986, 1274.5864, 1309.5436, 1309.5436, 1359.6350, 1361.6097, 1370.5820, 1375.6212, 1391.6156, 1397.6489, 1404.4968, 1407.6130, 1463.5942, 1475.6525, 1522.7041, 1538.6953, 1619.6931, 1638.7382, 1643.6715, 1649.7148, 1679.7761, 1683.6849, 1741.7567, 1747.7662, 1747.7662, 1795.7676, 1795.7676, 1809.8186, 1811.7775, 1811.7775, 1862.7720, 1864.7651, 1880.7800, 1912.7617, 1928.7726, 1944.7719, 1944.7719, 1960.7692, 1969.9302, 1976.7811, 1992.7775, 2008.7810, 2017.9209, 2033.9208, 2033.9208, 2055.8633, 2093.8899, 2210.9875, 2224.9951, 2229.9954, 2229.9954, 2239.0081, 2245.9932, 2322.9805, 2556.1221, 2691.0991, 2695.1079, 2702.0947, 2720.0598, 2748.1143, 2807.1252, 3346.5364 |
| **G 18 gi|4519264 Mass: 54329 Score: 641 Expect: 1.5e-058 Queries matched: 35 vacuolar H+-ATPase B subunit [Citrus unshiu]**  Observed Mr(expt) Mr(calc) Delta Start End Miss Ions Peptide  842.4846 841.4773 841.4406 0.0368 293 - 299 1 --- R.EEVPGRR.G  1045.4753 1044.4680 1044.4876 -0.0196 477 - 484 0 --- K.TLDQYYSR.D  1069.5494 1068.5421 1068.5603 -0.0182 42 - 49 0 50 K.YYEIVNIR.L  1069.5494 1068.5421 1068.5603 -0.0182 42 - 49 0 --- K.YYEIVNIR.L  1088.5573 1087.5500 1087.5695 -0.0195 95 - 104 0 --- K.TPVSLDMLGR.I  1104.5536 1103.5463 1103.5645 -0.0181 95 - 104 0 --- K.TPVSLDMLGR.I + Oxidation (M)  1113.5149 1112.5076 1112.5250 -0.0174 440 - 449 0 64 K.FVAQGAYDSR.N  1113.5149 1112.5076 1112.5250 -0.0174 440 - 449 0 --- K.FVAQGAYDSR.N  1213.4641 1212.4568 1212.4717 -0.0148 225 - 234 0 --- R.DFEENGSMER.V  1229.4592 1228.4519 1228.4666 -0.0147 225 - 234 0 --- R.DFEENGSMER.V + Oxidation (M)  1241.6111 1240.6038 1240.6200 -0.0162 439 - 449 1 --- R.KFVAQGAYDSR.N  1369.7268 1368.7195 1368.7289 -0.0093 83 - 94 0 --- K.FTTVQFTGEVLK.T  1563.8002 1562.7929 1562.7940 -0.0010 68 - 82 0 --- K.AVVQVFEGTSGIDNK.F  1596.8995 1595.8922 1595.9035 -0.0113 365 - 378 0 87 R.QIYPPINVLPSLSR.L  1596.8995 1595.8922 1595.9035 -0.0113 365 - 378 0 --- R.QIYPPINVLPSLSR.L  1715.9260 1714.9187 1714.9253 -0.0066 235 - 249 0 93 R.VTLFLNLANDPTIER.I  1715.9260 1714.9187 1714.9253 -0.0066 235 - 249 0 --- R.VTLFLNLANDPTIER.I  1903.9893 1902.9820 1902.9873 -0.0053 270 - 286 0 --- K.HVLVILTDMSSYADALR.E  1919.9999 1918.9926 1918.9822 0.0104 270 - 286 0 48 K.HVLVILTDMSSYADALR.E + Oxidation (M)  1919.9999 1918.9926 1918.9822 0.0104 270 - 286 0 --- K.HVLVILTDMSSYADALR.E + Oxidation (M)  1939.8809 1938.8736 1938.8821 -0.0085 300 - 315 0 85 R.GYPGYMYTDLAQIYER.A  1939.8809 1938.8736 1938.8821 -0.0085 300 - 315 0 --- R.GYPGYMYTDLAQIYER.A  1955.8944 1954.8871 1954.8771 0.0101 300 - 315 0 (49) R.GYPGYMYTDLAQIYER.A + Oxidation (M)  1955.8944 1954.8871 1954.8771 0.0101 300 - 315 0 --- R.GYPGYMYTDLAQIYER.A + Oxidation (M)  2050.9856 2049.9783 2049.9868 -0.0084 391 - 408 1 89 R.RDHSDVSNQLYANYAIGK.D  2050.9856 2049.9783 2049.9868 -0.0084 391 - 408 1 --- R.RDHSDVSNQLYANYAIGK.D  2340.1504 2339.1431 2339.1783 -0.0352 415 - 435 0 --- K.AVVGEEALSSEDLLYLEFLDK.F  2355.0598 2354.0525 2354.0518 0.0008 2 - 22 0 --- M.GVAQNNVDMEEGTLEVAMEYR.T  2371.0830 2370.0757 2370.0467 0.0290 2 - 22 0 --- M.GVAQNNVDMEEGTLEVAMEYR.T + Oxidation (M)  2387.0779 2386.0706 2386.0416 0.0290 2 - 22 0 --- M.GVAQNNVDMEEGTLEVAMEYR.T + 2 Oxidation (M)  2469.2036 2468.1963 2468.1927 0.0037 138 - 159 0 --- R.TYPEEMIQTGISTIDVMNSIAR.G  2485.2195 2484.2122 2484.1876 0.0246 138 - 159 0 --- R.TYPEEMIQTGISTIDVMNSIAR.G + Oxidation (M)  2501.2302 2500.2229 2500.1825 0.0404 138 - 159 0 --- R.TYPEEMIQTGISTIDVMNSIAR.G + 2 Oxidation (M)  2772.3481 2771.3408 2771.3904 -0.0496 415 - 438 1 --- K.AVVGEEALSSEDLLYLEFLDKFER.K  3454.7603 3453.7530 3453.7415 0.0115 105 - 137 0 --- R.IFNGSGKPIDNGPPILPEAYLDISGSSINPSER.T  No match to: 1139.6731, 1146.5718, 1197.6414, 1215.5651, 1231.5636, 1274.6676, 1408.6525, 1421.8077, 1431.6204, 1445.6388, 1486.6506, 1574.7855, 1578.8835, 1579.8717, 1579.8717, 1591.8478, 1638.8376, 1671.8590, 1693.9106, 1700.8856, 1702.8534, 1721.9089, 1834.9601, 1854.9229, 1855.9478, 1871.9624, 1887.9257, 1891.8723, 1891.8723, 1907.9019, 1916.8856, 1935.9390, 1937.9150, 1938.9086, 1972.9637, 1993.9478, 2066.9089, 2086.6245, 2087.0994, 2089.0503, 2148.1584, 2160.1375, 2178.1450, 2178.1450, 2179.6565, 2200.1262, 2207.0566, 2211.1057, 2219.1594, 2225.1130, 2230.1836, 2235.1511, 2259.0627, 2267.0222, 2269.0139, 2283.0283, 2306.1013, 2307.0603, 2323.0752, 2353.0955, 2373.1038, 2403.1711, 2419.1636, 2421.1880, 2437.2129, 2658.2820, 2695.1987, 2705.1848, 2733.3708, 2734.3679, 2751.3889, 2751.3889, 2800.3127, 2828.3513, 2832.3420, 2844.3274, 2859.2686 |
| **G 20 gi|4519264 Mass: 54329 Score: 121 Expect: 1.5e-006 Queries matched: 13 vacuolar H+-ATPase B subunit [Citrus unshiu]**  Observed Mr(expt) Mr(calc) Delta Start End Miss Ions Peptide  842.4755 841.4682 841.4406 0.0276 293 - 299 1 --- R.EEVPGRR.G  1045.4647 1044.4574 1044.4876 -0.0302 477 - 484 0 --- K.TLDQYYSR.D  1069.5319 1068.5246 1068.5603 -0.0357 42 - 49 0 --- K.YYEIVNIR.L  1104.5341 1103.5268 1103.5645 -0.0376 95 - 104 0 --- K.TPVSLDMLGR.I + Oxidation (M)  1113.4987 1112.4914 1112.5250 -0.0336 440 - 449 0 --- K.FVAQGAYDSR.N  1369.6763 1368.6690 1368.7289 -0.0598 83 - 94 0 --- K.FTTVQFTGEVLK.T  1596.8679 1595.8606 1595.9035 -0.0429 365 - 378 0 80 R.QIYPPINVLPSLSR.L  1596.8679 1595.8606 1595.9035 -0.0429 365 - 378 0 --- R.QIYPPINVLPSLSR.L  1715.8903 1714.8830 1714.9253 -0.0423 235 - 249 0 2 R.VTLFLNLANDPTIER.I  1715.8903 1714.8830 1714.9253 -0.0423 235 - 249 0 --- R.VTLFLNLANDPTIER.I  1939.8517 1938.8444 1938.8821 -0.0377 300 - 315 0 --- R.GYPGYMYTDLAQIYER.A  1955.8547 1954.8474 1954.8771 -0.0296 300 - 315 0 --- R.GYPGYMYTDLAQIYER.A + Oxidation (M)  2050.9565 2049.9492 2049.9868 -0.0375 391 - 408 1 --- R.RDHSDVSNQLYANYAIGK.D  No match to: 713.3865, 733.3376, 768.5025, 832.2690, 864.4308, 870.5084, 882.5306, 908.3616, 973.4913, 991.5510, 1060.5183, 1090.5493, 1139.6526, 1144.4779, 1146.5494, 1157.5525, 1179.5637, 1199.6007, 1205.5625, 1208.6023, 1231.5464, 1232.5529, 1235.5222, 1270.6624, 1270.6624, 1274.6549, 1277.6642, 1280.7299, 1280.7299, 1294.6329, 1307.6339, 1308.6266, 1320.5454, 1323.6361, 1365.6085, 1367.6941, 1383.6532, 1390.6271, 1418.6829, 1431.5973, 1461.6980, 1472.6422, 1475.7141, 1480.6550, 1480.6550, 1505.6844, 1579.8392, 1649.7186, 1657.7582, 1700.8608, 1700.8608, 1707.7412, 1744.8466, 1776.8605, 1791.7239, 1792.7615, 1792.7615, 1834.9537, 1834.9537, 1840.8185, 1856.8351, 1856.8351, 1872.8391, 1888.8251, 1890.8512, 1891.8436, 1900.0096, 1904.8361, 1920.8298, 1920.8298, 1993.9192, 2178.1028, 2178.1028, 2211.0894, 2225.0928, 2268.9797, 2283.0046, 2383.9160, 2383.9160, 2415.1123, 2430.2625, 2464.1438, 2695.1763, 2705.1050, 2717.0432, 2751.3223, 2775.4260, 2807.2637, 3346.6782 |
| **G 2 gi|296088008 Mass: 17163 Score: 503 Expect: 9.3e-045 Queries matched: 13 unnamed protein product, partial [Vitis vinifera]**  Observed Mr(expt) Mr(calc) Delta Start End Miss Ions Peptide  863.4052 862.3979 862.4548 -0.0570 115 - 121 0 --- K.LAEQFQK.Q  991.4959 990.4886 990.5498 -0.0612 114 - 121 1 13 R.KLAEQFQK.Q  991.4959 990.4886 990.5498 -0.0612 114 - 121 1 --- R.KLAEQFQK.Q  1287.6134 1286.6061 1286.6870 -0.0809 62 - 72 0 18 K.DDVVIQFLNPK.V  1287.6134 1286.6061 1286.6870 -0.0809 62 - 72 0 --- K.DDVVIQFLNPK.V  1726.8801 1725.8728 1725.9664 -0.0936 46 - 61 0 53 R.IGVNAIPAIEEVNIFK.D  1726.8801 1725.8728 1725.9664 -0.0936 46 - 61 0 --- R.IGVNAIPAIEEVNIFK.D  2331.1587 2330.1514 2330.2594 -0.1079 93 - 113 0 158 K.LQDILPGIINQLGPDNLDNLR.K  2331.1587 2330.1514 2330.2594 -0.1079 93 - 113 0 --- K.LQDILPGIINQLGPDNLDNLR.K  2459.2292 2458.2219 2458.3543 -0.1324 92 - 113 1 96 K.KLQDILPGIINQLGPDNLDNLR.K  2459.2292 2458.2219 2458.3543 -0.1324 93 - 114 1 --- K.LQDILPGIINQLGPDNLDNLRK.L  2995.5269 2994.5196 2994.6429 -0.1233 46 - 72 1 70 R.IGVNAIPAIEEVNIFKDDVVIQFLNPK.V  2995.5269 2994.5196 2994.6429 -0.1233 46 - 72 1 --- R.IGVNAIPAIEEVNIFKDDVVIQFLNPK.V  No match to: 744.3622, 787.3870, 842.4543, 864.4076, 876.9825, 1013.4387, 1277.6343, 1277.6343, 1690.7218, 1690.7218, 1748.8278, 1748.8278, 1927.9327, 1927.9327, 1931.9274, 1949.8956, 1959.9192, 1959.9192, 1965.8732, 1981.8800, 2210.9968, 2313.1438, 2313.1438, 2333.7136, 2353.1260, 2369.0991, 3292.3613 |
| **G 39 gi|296088008 Mass: 17163 Score: 709 Expect: 2.3e-065 Queries matched: 17 unnamed protein product, partial [Vitis vinifera]**  Observed Mr(expt) Mr(calc) Delta Start End Miss Ions Peptide  863.3895 862.3823 862.4548 -0.0726 115 - 121 0 --- K.LAEQFQK.Q  937.3793 936.3720 936.4512 -0.0793 31 - 38 1 32 K.TTTTDDKR.L  937.3793 936.3720 936.4512 -0.0793 31 - 38 1 --- K.TTTTDDKR.L  991.4756 990.4684 990.5498 -0.0814 114 - 121 1 46 R.KLAEQFQK.Q  991.4756 990.4684 990.5498 -0.0814 114 - 121 1 --- R.KLAEQFQK.Q  1287.5930 1286.5857 1286.6870 -0.1013 62 - 72 0 79 K.DDVVIQFLNPK.V  1287.5930 1286.5857 1286.6870 -0.1013 62 - 72 0 --- K.DDVVIQFLNPK.V  1726.8523 1725.8450 1725.9664 -0.1214 46 - 61 0 66 R.IGVNAIPAIEEVNIFK.D  1726.8523 1725.8450 1725.9664 -0.1214 46 - 61 0 --- R.IGVNAIPAIEEVNIFK.D  1943.8943 1942.8870 1943.0112 -0.1242 73 - 91 0 37 K.VQASIAANTWVVSGSPQTK.K  1943.8943 1942.8870 1943.0112 -0.1242 73 - 91 0 --- K.VQASIAANTWVVSGSPQTK.K  2331.1318 2330.1245 2330.2594 -0.1348 93 - 113 0 172 K.LQDILPGIINQLGPDNLDNLR.K  2331.1318 2330.1245 2330.2594 -0.1348 93 - 113 0 --- K.LQDILPGIINQLGPDNLDNLR.K  2459.1987 2458.1914 2458.3543 -0.1629 93 - 114 1 100 K.LQDILPGIINQLGPDNLDNLRK.L  2459.1987 2458.1914 2458.3543 -0.1629 93 - 114 1 --- K.LQDILPGIINQLGPDNLDNLRK.L  2995.4922 2994.4849 2994.6429 -0.1580 46 - 72 1 115 R.IGVNAIPAIEEVNIFKDDVVIQFLNPK.V  2995.4922 2994.4849 2994.6429 -0.1580 46 - 72 1 --- R.IGVNAIPAIEEVNIFKDDVVIQFLNPK.V  No match to: 711.3436, 713.3571, 744.3445, 768.4654, 842.4390, 870.4695, 876.9780, 882.4896, 885.3726, 901.3443, 1012.3897, 1013.4352, 1045.4789, 1309.5730, 1325.5515, 1343.6616, 1475.6315, 1657.6523, 1679.7693, 1690.7058, 1748.8167, 1764.7972, 1909.8503, 1927.9033, 1927.9033, 1931.9011, 1949.8613, 1953.8595, 1959.8888, 1959.8888, 1965.8414, 1969.8297, 1974.9382, 1981.8408, 1993.8188, 1997.8281, 2143.9683, 2210.9778, 2224.9832, 2230.0608, 2233.0059, 2239.0068, 2247.0388, 2269.0110, 2272.0598, 2283.0564, 2285.0994, 2287.1384, 2296.0845, 2300.0742, 2313.1340, 2313.1340, 2314.1270, 2327.1055, 2329.1348, 2330.1531, 2338.0828, 2343.1050, 2345.1069, 2347.0906, 2351.0476, 2353.1003, 2358.0691, 2369.0469, 2369.0469, 2375.0574, 2391.0186, 2429.0234, 2435.0586, 2440.1055, 2441.1594, 2442.1846, 2456.2251, 2473.1743, 2475.1599, 2481.1829, 2497.1738, 2499.1819, 2503.1313, 2540.2661, 2612.2832, 2662.2065, 2677.1089, 2691.1064, 2720.0991, 2807.1169, 2811.1113, 2825.1140, 2914.3269, 3017.4478, 3033.4084, 3292.3508, 3337.5024, 3348.4319, 3353.4922 |

**Table S5 |** Specific primer pairs used for qRT-PCR expression analysis in citrus roots

| *Genes* | *Proteins* | *Forward primers (5´→3´)* | *Reverse primers (5´→3´)* |
| --- | --- | --- | --- |
| S27 | Adenosine kinase 2, partial | TGTTGTGGGTGGTGAAA | GGAGTCTGGAGATACGGT |
| S47 | Malate dehydrogenase , partial | GGTATTGTTGCTACTACGGAT | GTTCTTCGCAGGGATTG |
| S31 | Ketol-acid reductoisomerase | ATTGGCGTTATTGGTTG | GTCCGCTCCTGTGGTTA |
| S12 | Elongation factor 1-delta 1 | CTTTCTAAACCACCGTCA | CATCATTATCATCGTCAGC |
| S36 | Eukaryotic translation initiation factor 5A1 | TCAAAGACTGGCAAGCA | CAGATTTTCATCGGTAGG |
| S39 | 5-methyltetrahy dropteroyltriglutamate-homocysteine methyltransferase, putative | TTGCCAAATCTTCCTAC | AACCCACCCATTCACAG |
| S43 | Alanine aminotransferase 2 | TTACAAACTAACCCTGGCTCT | TCCGCACTGAACAAACCT |
| S41 | S-adenosyl-L-methionine synthetase 2 | AACGAGGGTTATCCAGA | CAAGCCAACTTCCTATCT |
| S21 | Glycine-rich RNA-binding protein | TCACTGAGGCTACAATAAA | AAAATAACTCGTCCATCC |
| S20 | Vacuolar H^+^-ATPase B subunit | AGGTCTTCTGGGGTCTG | AGCCTTTGTCCTGATGT |
| G32 | Cu/Zn superoxide dismutase, partial | GAGGGTATTGCTACTTTTG | TCTGGACTGGGACTTGA |
| G26 | Lactoylglutathione lyase, putative | ATGCCTTTCTTGGATTT | GTGGTTCAGGAGTTGGA |
| G16 | Heat shock protein 83 | AAGGAGTTTATGGAGGCT | TCGTCATTGTGCTTTGTA |
| G46 | ATPase alpha subunit , partial (mitochondrion) | CGGTAGATAGTCTCGTTCC | GTTCATTTCCTTTTGGTTT |
| G22 | Triosephosphate isomerase | GAGTCTGTTGCGGTCTG | CTGATTCTCGCTGTTCC |
| G28 | Ketol-acid reductoisomerase | ATTGGCGTTATTGGTTG | GTCCGCTCCTGTGGTTA |
| G11 | Eukaryotic translation initiation factor 5A1 | TCAAAGACTGGCAAGCA | CAGATTTTCATCGGTAGG |
| G43 | S-adenosylmethionine synthetase 1 family protein | CCATTGGATTTGTTTCTG | CTTCCTAACCTCCGTGA |
| G21 | Glycine-rich RNA-binding protein | TCACTGAGGCTACAATAAA | AAAATAACTCGTCCATCC |
| G20 | Vacuolar H^+^-ATPase B subunit | AGGTCTTCTGGGGTCTG | AGCCTTTGTCCTGATGT |
|  |  |  |  |
| gi|985455672 | GAPDH | GGAATCAGTAGCCGACAC | CCTAATAACTGGCAGAACA |
| gi|985473508 | RPII | AATCCTGATGCTGTTAGAC | GCAAAAGGGAAGAGTGT |


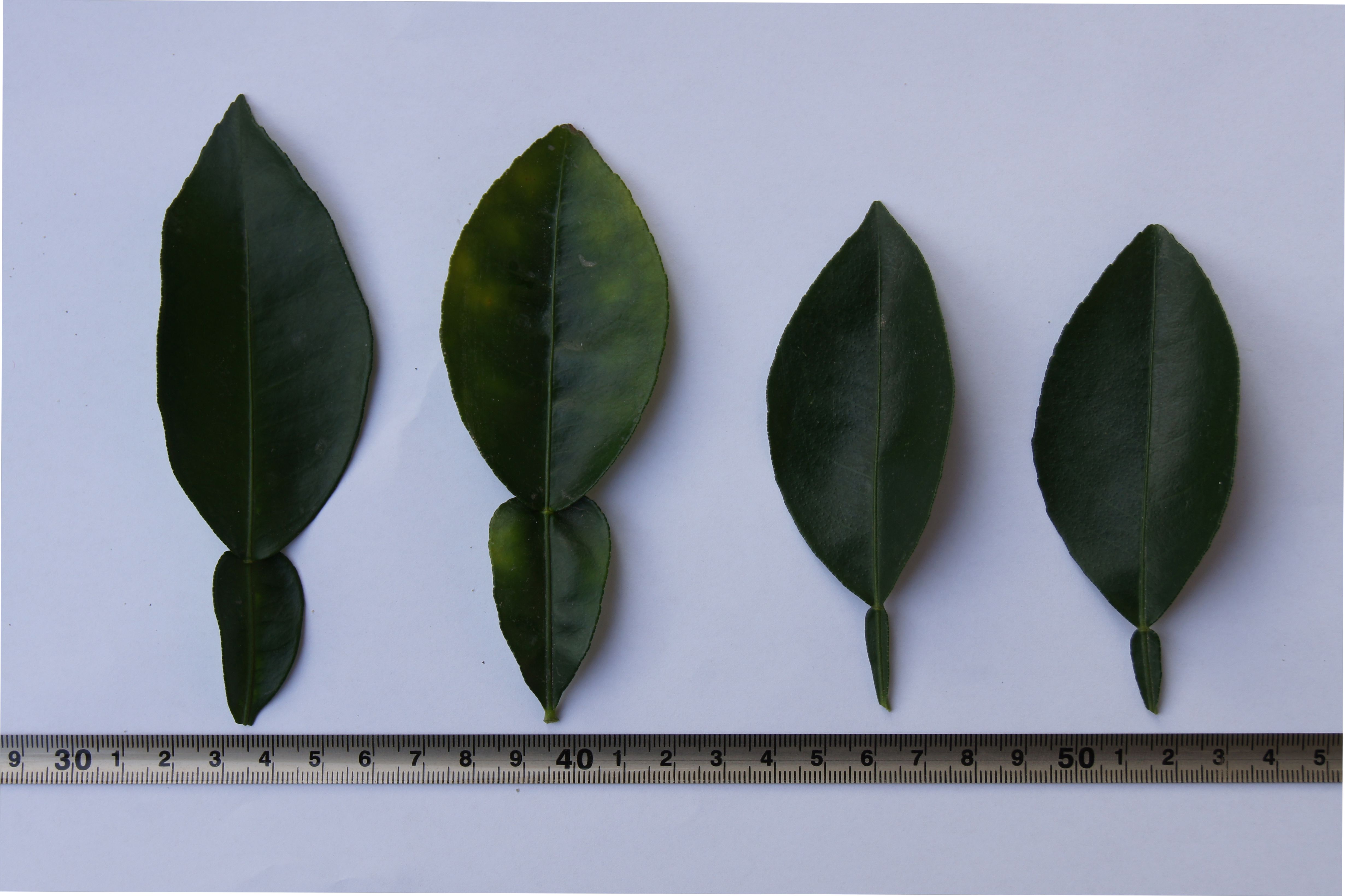


**1 2 3 4**


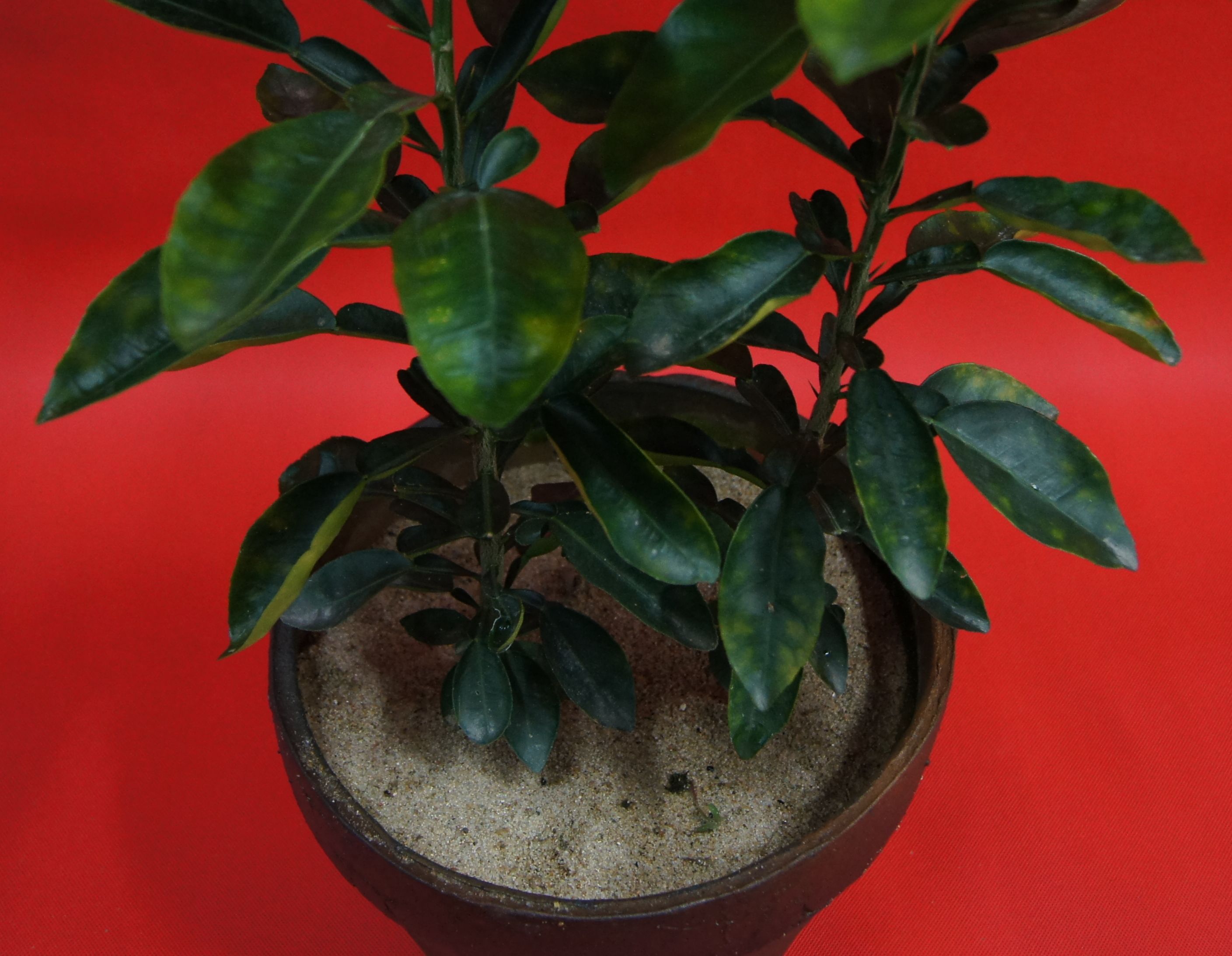


**A**

**B**

**Figure S1 | Boron (B)-toxic symptoms on *Citrus grandis* plants (A), *C. grandis* and *Citrus sinensis* leaves (B) treated with 10 μM (control) or 400 μM (B-toxic) H_3_BO_3_ for 15 weeks.** 1, Control leaves of *C. grandis*; 2, B-toxic leaves of *C. grandis*; 3, Control leaves of *C. sinensis*; 4, B-toxic leaves of *C. sinensis*.

pH 4 ———————— pH 7 pH 4 ———————— pH 7

SDS-PAGE

**A_1_**

SDS-PAGE

**C_1_**

SDS-PAGE

**A_2_**

SDS-PAGE

**C_2_**

**B_1_**

**D_1_**

**B_2_**

**D_2_**


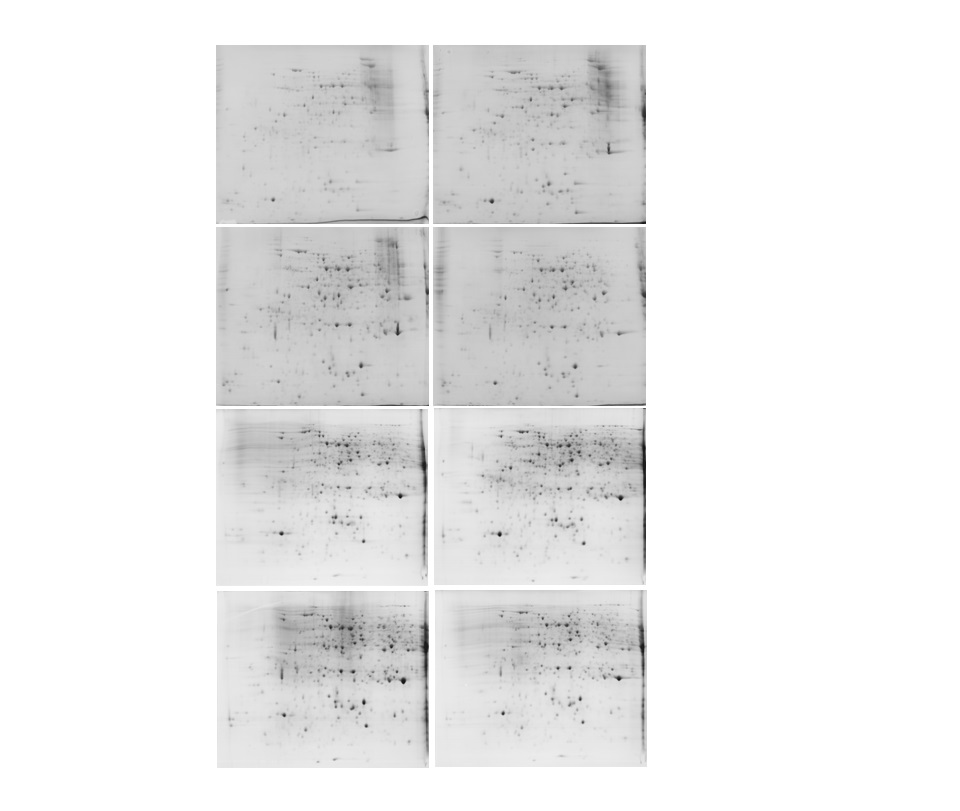


**Figure S2 | 2-DE images of proteins extracted from control (A and C) and B-toxic (B and D) roots for the other two replicates.** **(A_1_** and **A_2_)** Control roots of *Citrus sinensis*, **(B_1_** and **B_2_)** B-toxic root of *C. sinensis*, **(C_1_** and **C_2_)** Control roots of *Citrus grandis*, **(D_1_** and **D_2_)** B-toxic roots of *C. grandis.*
